# Supplementary material for: Providing ambulatory healthcare for people aged 80 and over: Views and perspectives of physicians and dentists from a qualitative survey
Source: PLoS One. 2022 Aug 15;17(8):e0272866. doi: 10.1371/journal.pone.0272866 (PMC9377615; doi:10.1371/journal.pone.0272866)
Supplement: S1 Appendix — (PDF) [file pone.0272866.s001.pdf]

Providing ambulatory healthcare for people aged 80 and over: views and perspectives of physicians and dentists from a qualitative survey. PLOS ONE.

S1 Appendix: Survey development, pretest report and final survey

Round 1 2

Round 2 16

Round 3 (final survey) 29

The German original version of the items and comments is provided in brackets.

The final survey is presented within round 3.

Round 1

- Period: 19/04/2021- 25/04/2021
- Participants: n = 5 colleagues, professionals in the fields of health sciences, sociology, gerontology, speech therapy
- Goal: general comprehensibility and arrangement of items, comments from a specialized perspective
- General findings:
    - Clear wording – when is relation to health services explicitly and important, when not?
    - uniform gendering with gender star
    - Split some items into two items to achieve a clearer focus or to ensure that, for example, the request for an explanation is not overread

**Original item**	**Answer options**	**Comments**	**Summary based on provided answers and comments**	**New item**
**Please specify your age.**<br><br>**[Bitte geben Sie Ihr Alter an.]**	<29 years<br>[< 29 Jahre]	none	works as intended	remains unchanged
	20-29 years<br>[20-29 Jahre]			
	30-39 years<br>[30-39 Jahre]			
	40-49 years[<br>40-49 Jahre]			
	50-59 years<br>[50-59 Jahre]			
	60-69 years<br>[60-69 Jahre]			
	70-79 years<br>[70-79 Jahre]			
	>79 years<br>[> 79 Jahre]			
**Please specify your gender.**<br><br>**[Bitte geben Sie Ihr Geschlecht an.]**	male<br>[Männlich]	none	works as intended	remains unchanged
	female<br>[Weiblich]			
	diverse<br>[divers]			
	no specification			

Original item	Answer options	Comments	Summary based on provided answers and comments	New item
	[Keine Angabe]			
**Do you practice in employment or in your own practice?**<br><br>**[Praktizieren Sie in Anstellung oder in eigener Praxis?]**	In employment [In Anstellung]	none	works as intended	remains unchanged
	In my own practice [In eigener Praxis]			
	Other (text field) [Sonstiges (Textfeld)]			
**How long have you been working in ambulatory healthcare?**<br>Please enter an integer.<br><br>**[Wie lange praktizieren Sie bereits in der ambulanten Versorgung?**<br>Bitte geben Sie eine ganze Zahl an.**]**	Numeric field<br><br>[Zahlenfeld]	none	works as intended	remains unchanged
**Please enter the first two digits of the postal code of the practice where you (mainly) work.**<br><br>**[Bitte geben Sie die ersten zwei Ziffern der Postleitzahl der Praxis an, in der Sie (hauptsächlich) arbeiten.]**	Numeric field<br><br>[Zahlenfeld]	As a participating person, one might ask what this is important for. Perhaps a small note could be inserted - to ensure complete anonymity.<br><br>[„Als teilnehmende Person könnte man sich die Frage stellen, wofür das wichtig ist. Vielleicht kann man eine kleine Notiz einblenden – zur Sicherung der vollständigen Anonymität.“]	works as intended, anonymity addresses within the info text on the first page	remains unchanged
**What is the proportion of privately insured patients you treat?**<br>Please give an estimate.	< 10%	none	works as intended, uniform gendering with gender star	**What is the proportion of private patients you treat?**<br>Please give an estimate.
	11-25%			
	26-40%			
	41-65%			
	66-80%			

Original item	Answer options	Comments	Summary based on provided answers and comments	New item
**[Wie hoch ist der Anteil der von Ihnen behandelten Privatpatient/innen?**<br>Bitte geben Sie eine Schätzung an.**]**	>80%			**[Wie hoch ist der Anteil der von Ihnen behandelten Privatpatient*innen?**<br>Bitte geben Sie eine Schätzung an.]
**In which area of healthcare are you mainly active?**<br><br>**[In welchem Bereich sind Sie hauptsächlich tätig?]**	Selection list<br><br>[Auswahlliste]	none	works as intended	remains unchanged
**Do you have a dental focus of activity or do you work in a dental specialist practice?**<br><br>**[Haben Sie einen zahnmedizinischen Tätigkeitsschwerpunkt oder arbeiten Sie in einer Zahnmedizinischen Facharztpraxis?]**	No<br>[Nein]<br>Yes → „Please indicate the focus of acitivity“, text field<br><br>[Ja → „Bitte geben Sie den Schwerpunkt an“, Textfeld]	none	works as intended	remains unchanged
**In your opinion, how much time was spent during your academic education and training to discuss the care of patients over 80 years of age?**<br>(visual scale)<br><br>**[Wie viel Zeit wurde Ihrer Einschätzung nach während Studium und Facharztweiterbildung aufgewendet, um die Versorgung über 80-jähriger Patient/-innen zu thematisieren?**<br>(visuelle Skala)**]**	Little<br><br>[wenig]<br>Rather little<br><br>[eher wenig]<br>Rather much<br><br>[eher viel]<br>Much<br><br>[viel]	„actually you only use one gender star…“<br><br>[„eig. Macht man ja nun ein Gendersternchen…“]<br><br>„perhaps a colour other than black? I would also write 'during your studies and further training as a specialist'. And does this age specification make sense here? As a study content, I rather imagine high age in general and less specifically the exact age limit. And is the question about specialist	Adaptation of wording for a better understanding of the age phase,<br><br>Normal selection scale instead of visual scale<br><br>uniform gendering with gender star<br><br>Divide into one item on study and one item on specialist training	**1) In your opinion, how much time was spent during your academic education to discuss the care of over 80-year-old or very old patients?**<br><br>**[1) Wie viel Zeit wurde Ihrer Einschätzung nach während des Studiums aufgewendet, um die Versorgung über 80-jähriger bzw. hochaltriger Patient*innen zu thematisieren?]**

Original item	Answer options	Comments	Summary based on provided answers and comments	New item
		care or general care and supply structures?“<br><br>[„vielleicht eine andere Farbe als schwarz? Würde zudem ‚während des Studiums und Ihrer Facharztweiterbildung‘ schreiben. Und macht hier diese Altersangabe Sinn? Als Studieninhalt stelle ich mir eher Hochaltrigkeit allgemein vor und weniger konkret die genaue Altersgrenze. Und geht es dir in der Frage um fachärztliche Versorgung oder allgemeine Versorgungs- und Angebotsstrukturen?“]<br><br>„KO01 for me it would not need the layout, the scale answers are clear and easy to grasp:“<br><br>[„KO01 für mich bräuchte es nicht das Layout, die Skalenantworten sind klar und einfach zu erfassen:“]	as there are different phases	**2) In your opinion, how much time was spent during specialist training to address the issue of care for over 80-year-old or very old patients?**<br><br>**[2) Wie viel Zeit wurde Ihrer Einschätzung nach während der Facharztweiterbildung aufgewendet, um die Versorgung über 80-jähriger bzw. hochaltriger Patient*innen zu thematisieren?]**
**How many further training courses have you attended in the last three years that dealt with the care of patients aged 80 and over?**<br><br>**[Wie viele Fortbildungen haben Sie darüber hinaus in den letzten drei Jahren besucht, die die Versorgung über 80-jähriger Patient/-innen thematisierten?]**	< 3<br>3-6<br>7-10<br>> 10	„KO02 highlight ‚three years‘?“<br><br>[„KO02 ‚drei Jahre‘ hervorheben?“]	works as intended, uniform gendering with gender star<br><br>Since the item is already shown in bold in SoSciSurvey, no further emphasis needed	**How many further training courses have you attended in the last three years that dealt with the care of patients aged 80 and over?**<br><br>**[Wie viele Fortbildungen haben Sie darüber hinaus in den letzten drei Jahren besucht, die die Versorgung**

Original item	Answer options	Comments	Summary based on provided answers and comments	New item
				**über 80-jähriger Patient*innen thematisierten?]**
**In your opinion, are there enough training courses offered for you that deal with the care of patients aged 80 and over?**<br><br>**[Werden Ihrer Meinung nach ausreichend Fortbildungen für Sie angeboten, die die Versorgung über 80-jähriger Patient/-innen thematisieren?]**	Yes<br><br>[Ja]<br>No<br><br>[Nein]	KO03 Are you also interested in what further training they would like to receive? If so, this question could be well connected here<br><br>[„KO03 ist es für dich auch interessant, welche Fortbildungen sie sich wünschen würden? Falls ja, könnte diese Frage hier gut angeschlossen werden."]	works as intended	**In your opinion, are there enough training courses offered for you that deal with the care of patients aged 80 and over?**<br><br>**[Werden Ihrer Meinung nach ausreichend Fortbildungen für Sie angeboten, die die Versorgung über 80-jähriger Patient*innen thematisieren?]**
**How high is the proportion of people aged 80 and over in your daily work?** Please provide an estimate.<br><br>**[Wie hoch schätzen Sie den Anteil der über 80-Jährigen in Ihrer täglichen Arbeit ein?** Bitte geben Sie eine Schätzung an.**]**	< 10%<br>11-25%<br>26-40%<br>41-65%<br>66-80%<br>>80%	"KO04 maybe another question, is this recorded accurately (through insurance scheme statements for example)?"<br><br>[„KO04 vielleicht noch eine Frage, ob das genau erfasst wird (durch KK Abrechnungen zum Beispiel)?"]	works as intended<br><br>Since this should only serve as an analysis aid for a rough assessment or subdivision into subgroups, the recording by the insurance scheme is not relevant	remains unchanged
In the following, we would like to gain an insight into your work with patients aged 80 and over. For this purpose, we will now ask you a few open questions, which you can answer via a free text field. Please note three things:<br>1) You are free to answer the questions in bullet points or to describe them in more detail. However, whenever you can and would like to, we welcome brief explanations of your thoughts and reasons. This helps us to better understand your views.<br>2) "Care" does not only include medical aspects, but everything that is part of your daily or regular services.		"KO05 'what you consider part of the care provided on a daily basis' sounds a bit bumpy; maybe leave out the 'to'. I think the explanation is generally important, and could perhaps also be mentioned in the introduction."<br><br>[„KO05 ‚was für Sie zur in der täglich erbrachten versorgung	Adaptation of wording for a better understanding of the age phase, einheitliches Gendern mit Genderstern,	In the following, we would like to gain an insight into your work with patients aged 80 and over. For this purpose, we will now ask you a few open questions, which you can answer via a free text field. Please keep three things in mind:

**Original item**	**Answer options**	**Comments**	**Summary based on provided answers and comments**	**New item**
3) Please avoid giving personal data such as names.<br><br>[Im Folgenden möchten wir einen Einblick in Ihre Arbeit mit über 80-jährigen Patient/-innen gewinnen. Dazu stellen wir Ihnen nun ein paar offene Fragen, die Sie über ein Freitextfeld beantworten können. Bitte beachten Sie dabei drei Dinge:<br>1) Es steht Ihnen frei, die Fragen stichpunktartig zu beantworten oder etwas ausführlicher zu beschreiben. Wann immer Sie können und möchten, freuen wir uns jedoch über kurze Ausführungen Ihrer Gedanken und Begründungen. Das hilft uns, Ihre Ansichten besser zu verstehen.<br>2) Unter „Versorgung" werden nicht nur medizinische Aspekte, sondern alles, was für Sie zu Ihren täglich oder regelmäßig erbrachten Leistungen dazugehört.<br>3) Bitte vermeiden Sie die Angabe personenbezogener Daten wie bspw. Namen.]		dazugehört' klingt etwas holprig; vielleicht das ‚zur' weglassen. Finde die Erläuterung generell wichtig, könnte vielleicht auch schon in der Einleitung erwähnt werden."]<br><br>"KO05 I would write the second point more as a fact, i.e. without "we": e.g. "care" here does not only refer to medical aspects, but to everything that is part of the care provided for you on a daily basis."<br><br>[„KO05 ich würde den zweiten Punkt eher als ein Fakt schreiben, also ohne „wir": z.B. „Versorgung" bezieht sich hier nicht nur auf medizinische Aspekte, sondern auf alles, was für Sie zur in der täglich erbrachten Versorgung dazugehört."]		1) You are free to answer the questions in bullet points or to describe them in more detail. However, whenever you can and would like to, we welcome brief explanations of your thoughts and reasons. This helps us to better understand your views.<br>2) "Care" is not only understood to mean medical aspects, but everything that is part of the services you provide on a daily or regular basis.<br>3) Please avoid giving personal data such as names.<br><br><br>[Im Folgenden möchten wir einen Einblick in Ihre Arbeit mit über 80-jährigen Patient*innen gewinnen. Dazu stellen wir Ihnen nun ein paar offene Fragen, die Sie über ein Freitextfeld beantworten können. Bitte beachten Sie dabei drei Dinge:<br>1) Es steht Ihnen frei, die Fragen stichpunktartig zu beantworten oder etwas ausführlicher zu beschreiben. Wann immer Sie können und möchten, freuen wir uns jedoch über kurze Ausführungen Ihrer

Original item	Answer options	Comments	Summary based on provided answers and comments	New item
				Gedanken und Begründungen. Das hilft uns, Ihre Ansichten besser zu verstehen.<br>2) Unter „Versorgung“ werden nicht nur medizinische Aspekte verstanden, sondern alles, was für Sie zu Ihren täglich oder regelmäßig erbrachten Leistungen dazugehört.<br>3) Bitte vermeiden Sie die Angabe personenbezogener Daten wie bspw. Namen.]
**From your perspective, what are the special characteristics of patients aged 80 and over?**<br>Think of typical situations, for example.<br><br>**[Welche besonderen Charakteristika kennzeichnen aus Ihrer Perspektive die über 80-jährigen Patient/-innen?**<br>Denken Sie hierbei zum Beispiel an typische Situationen.**]**	text field<br><br>[Textfeld]	none	works as intended, uniform gendering with gender star	remains unchanged
**What is special about your work with patients aged 80 and over?**<br>Think, for example, of the special features that differ from working with younger patients.<br><br>**[Welche Besonderheiten gibt es in Ihrer Arbeit mit den über 80-jährigen Patient*innen?**<br>Denken Sie hierbei zum Beispiel an Besonderheiten im Unterschied zur Arbeit mit jüngeren Patient*innen.]	text field<br><br>[Textfeld]	„CV02: I find the question slightly suggestive; it could perhaps be worded more openly: In your experience, are there any special features in the work with/care of patients over 80 years of age? If so, what are they?“<br><br>[„CV02: Finde die Frage leicht suggestiv; könnte man evtl. offener formulieren: Gibt es Ihrer Erfahrung nach Besonderheiten in	works as intended, uniform gendering with gender star<br><br>In order to keep the intended focus, first test with the real target group in the next round, then adjust if necessary - if necessary merge	remains unchanged

Original item	Answer options	Comments	Summary based on provided answers and comments	New item
		der Arbeit mit/Versorgung über 80-jähriger Patient/-innen? Wenn ja, welche sind das?“]<br><br>"CV02 In the survey, you can go back to question 12 and think more about "typical" characteristics. If several of them already make references to younger patients in typical situations, you could think about how the characteristic "typical" can be emphasised even more.“<br><br>[„CV02 man kann dann ja in der Befragung nochmal zu Frage 12 zurückgehen und mehr an „typische“ Merkmale denken. Falls aus mehreren so geht, dass sie schon in den typischen Situationen Referenzen zu jüngeren Patient*innen stellen, könntest du nochmal überlegen, wie sich das Merkmal „typisch“ noch besser hervorheben lässt.“]		
**What is the first thing you think of regarding good care for patients aged 80 and over and why do you think it is important?**<br><br>**[Woran denken Sie als Erstes, wenn es um eine gute Versorgung über 80-jähriger Patient/-innen geht und warum finden Sie das wichtig?]**	text field<br><br>[Textfeld]	"Is the intention here to give only one answer? Sounds like it to me. I would repeat myself here now and describe one of the things that came up in the previous question: so, for example, time."<br><br>[„Ist hier beabsichtigt, nur eine Antwort zu geben? Klingt für mich	works as intended<br><br>Comment: since associations are involved, duplication is fine<br><br>Adaptation for leaner wording	**What is the first thing you think of regarding good care for people aged 80 and over and why do you think it is important?**<br><br>**[Woran denken Sie als Erstes, wenn es um eine gute Versorgung über 80-Jähriger**

Original item	Answer options	Comments	Summary based on provided answers and comments	New item
		so. Ich würde mich jetzt hier wiederholen und eines der Dinge beschreiben, die in der vorherigen Frage aufkam: also z.B. Zeit.“]		**geht und warum finden Sie das wichtig?]**
**Which three aspects do you find challenging in caring for people aged 80 and over and why?**<br>Please write your short justification right after the mentioned aspect in the same line.<br><br>**[Welche drei Aspekte empfinden Sie als herausfordernd in der Versorgung über 80-Jähriger und warum?**<br>Bitte schreiben Sie Ihre kurze Begründung gleich hinter den genannten Aspekt in dieselbe Zeile.]	3x text field<br><br>[3x Textfeld]	„One quickly overlooks the second part of the question: the rationale“<br><br>[„Man überliest schnell den zweiten Teil der Frage: die Begründung“]<br><br>"HC02 Perhaps it would be interesting to have the framing, i.e. the aspect and the justification, separate? That way there might be more justification and not just a keyword given for aspect three. At this point (half of the questionnaire) it is important that respondents 'stick to it'."<br><br>[„HC02 vielleicht wäre es interessant das Framing, also den Aspekt und die Begründung getrennt zu haben? So wird evtl. mehr begründet und bei Aspekt drei nicht nur ein Stichwort gegeben. An dieser Stelle (Hälfte des Fragebogens) ist es wichtig, dass die Befragten ‚an der Stange bleiben‘“]	Separation of mention and justification in an extra item so that the justification is not overlooked and is given separately;<br>also bypassing the concept of care so that the personal level can also be addressed if necessary	**1) Which three aspects do you find challenging in working with people aged 80 and over?**<br><br>**[1) Welche drei Aspekte empfinden Sie als herausfordernd in der Arbeit mit über 80-Jährigen?]**<br><br>**2) Why do you find these aspects challenging?**<br><br>**[2) Warum empfinden Sie diese Aspekte als herausfordernd?]**
Which three aspects do you find enriching in caring for people aged 80	3x text field<br><br>[3x Textfeld]	„HC03: rather 'in dealing with patients over 80'? enriching care' - what is that supposed to mean in	Separation of mention and justification in an extra item so that the	**1) Which three aspects do you find enriching in working with people aged 80 and over?**

Original item	Answer options	Comments	Summary based on provided answers and comments	New item
and over or what potential do you see and why?<br>Please write your short justification right after the mentioned aspect in the same line.<br><br>[Welche drei Aspekte empfinden Sie als bereichernd in der Versorgung über 80-Jähriger bzw. welche Potenziale sehen Sie und warum?<br>Bitte schreiben Sie Ihre kurze Begründung gleich hinter den genannten Aspekt in dieselbe Zeile.]		concrete terms? And 'potentials' also goes in another direction for me - more in the direction of potentials for improving care."<br><br>[„HC03: eher ‚im Umgang mit über 80-jährigen Patient*innen'? ‚bereichernd in der Versorgung' – auf was soll das konkret abzielen? Und ‚Potenziale' geht für mich auch nochmal in eine andere Richtung – eher Richtung Potenziale zur Verbesserung der Versorgung."]<br><br><br>„"HC03 ' Which three aspects do you find enriching in the care of over 80-year-olds or which potentials do you see and why?' -> I would not ask for two different dimensions in one question"<br><br>[„HC03 ‚ Welche drei Aspekte empfinden Sie als bereichernd in der Versorgung über 80-Jähriger bzw. welche Potenziale sehen Sie und warum?' -> ich würde nicht zwei doch unterschiedliche Dimensionen in einer Frage erheben"]	justification is not overlooked and is given separately;<br>also bypassing the concept of care so that the personal level can also be addressed if necessary;<br>Deletion of the term "potential" because a different concept is being addressed here	 **[1) Welche drei Aspekte empfinden Sie als bereichernd in der Arbeit mit über 80-Jährigen?]**<br><br>**2) Why do you find these aspects enriching?**<br><br>**[2) Warum empfinden Sie diese Aspekte als bereichernd?]**
What is going (rather) well or (rather) badly in your work with people aged 80 and over and why?	Text field: This is going (rather) well:	„HC06: I find the question quite „general; is it specifically about the respective specialist care, is it	The order is intended so that the following item (desired	**1) What is going (rather) well in your work with people aged 80 and over and why?**

Original item	Answer options	Comments	Summary based on provided answers and comments	New item
[Was läuft in Ihrer Arbeit mit den über 80-Jährigen aktuell (eher) gut oder (eher) schlecht und warum?]	[Textfeld: Das läuft (eher) gut:]<br>Text field: This is going (rather) badly:<br><br>[Textfeld: Das läuft (eher) schlecht:]	about everyday practice? Perhaps one could also swap the order of the questions: These first and then go to challenges and potentials. Then you would have the concrete first and then the abstract.“<br><br>[„HC06: Finde die Frage recht allgemein; geht es konkret um die jeweilige fachärztliche Versorgung, geht es um den Praxisalltag? Vielleicht könnte man die Reihenfolge der Fragen auch tauschen: Diese zuerst und dann zu Herausforderungen und Potenzialen gehen. Dann hätte man das Konkrete zuerst und folgend die Abstraktion.“]	improvement measures) can be drawn from experience: Separation into two items for better understanding	**[1) Was läuft in Ihrer Arbeit mit den über 80-Jährigen (eher) gut und warum?]**<br><br>**1) What is going (rather) bad in your work with people aged 80 and over and why?**<br><br>**[2) Was läuft in Ihrer Arbeit mit den über 80-Jährigen (eher) schlecht und warum?]**
**What measures would you like to see in order to improve care for people aged 80 and over and why?**<br>This does not only have to address the patients directly, but also think about what would be helpful for you in your work and role as a medical professional.<br><br>**[Welche Maßnahmen würden Sie sich zur Verbesserung der Versorgung über 80-Jähriger wünschen und warum?**	text field<br><br>[Textfeld]	„MV01 I like the question!”<br><br>[„MV01 Die Frage finde ich gut!“]<br><br>"MV01 ' What measures would you like to see to improve care for the over 80s and why?' Perhaps better: What measures would you like to see..."“<br><br>[„MV01 , Welche Maßnahmen würden Sie sich zur Verbesserung der Versorgung über 80-Jähriger wünschen und warum?‘ Vllt	works as intended, uniform gendering with gender star, Adaptation of wording for a more direct focus	**What measures do you wish for to improve care for people aged 80 and over and why?**<br>This does not only have to address the patients directly, but also think about what would be helpful for you in your work and role as a medical professional.<br><br>**[Welche Maßnahmen wünschen Sie sich zur Verbesserung der Versorgung über 80-Jähriger und warum?**

Original item	Answer options	Comments	Summary based on provided answers and comments	New item
Dabei muss es nicht nur direkt um die Patient/-innen gehen, denken Sie auch daran, was für Sie in Ihrer Arbeit und Rolle als Mediziner/-in hilfreich wäre.]		besser: Welche Maßnahmen wünschen Sie sich…“]		Dabei muss es nicht nur direkt um die Patient*innen gehen, denken Sie auch daran, was für Sie in Ihrer Arbeit und Rolle als Mediziner*in hilfreich wäre.]
**Do you feel well educated or trained to care for patients aged 80 and over?**<br><br>**[Fühlen Sie sich gut für die Versorgung über 80-jähriger Patienten aus- bzw. fortgebildet?]**	No<br>[Nein]	„AP01 or AP02: patient(s)“<br><br>[„AP01 bzw. AP02: Patient/Innen“]	works as intended, uniform gendering with gender star	**Do you feel well educated or trained to care for patients aged 80 and over?**<br><br>**[Fühlen Sie sich gut für die Versorgung über 80-jähriger Patient*innen aus- bzw. fortgebildet?]**
	rather no<br>[eher nein]			
	rather yes<br>[eher ja]			
	yes<br>[ja]			
**In how many cases can you care for patients aged 80 and over in a way that you personally think is right and important?**<br>Please provide an estimate.<br><br>**[In wie vielen Fällen können sie über 80-jährige Patienten so versorgen, wie Sie persönlich es für richtig und wichtig halten?**<br>Bitte geben Sie eine Schätzung an.]	< 25%	" AP02: 'Can you' and do you gender patients otherwise?"<br><br>[„AP02: ‚können Sie“ und genderst du Patient*innen sonst?“]<br><br>" AP02: What is most often lacking in care?"<br><br>[„AP02: Woran fehlt es am häufigsten in der Versorgung?“]	Works as intended, uniform gendering with gender star Additional item to be able to disclose reasons for subjectively insufficient care	**In how many cases can you care for patients aged 80 and over in a way that you personally think is right and important?**<br>Please provide an estimate.<br><br>**[1) In wie vielen Fällen können sie über 80-jährige Patient*innen so versorgen, wie Sie persönlich es für richtig und wichtig halten?**<br>Bitte geben Sie eine Schätzung an.]<br><br>**2) If you cannot care for patients aged 80 and over in the way you personally think is right and important – what is the reason?**<br>text field
	25-49%			
	50-75%			
	> 75%			

Original item	Answer options	Comments	Summary based on provided answers and comments	New item
				**[2) Wenn Sie über 80-jährige Patient*innen nicht so versorgen können, wie Sie persönlich es für richtig und wichtig halten – woran liegt das?**<br>(Textfeld)]
In previous studies, we have found that three basic motives shape the care wishes of people aged 80 and over: to feel safe, to feel like a meaningful human being, and to maintain autonomy and independence.<br>**What is the first thing you think of with regard to ensuring that patients aged 80 and over...**<br><br>[In vorangegangenen Untersuchungen haben wir herausgefunden, dass drei grundlegende Motive die Versorgungswünsche über 80-Jähriger prägen: sich sicher zu fühlen, als bedeutsame Menschen wahrgenommen zu werden sowie Autonomie und Unabhängigkeit zu bewahren.<br>**Woran denken Sie als Erstes, wenn es darum geht zu gewährleisten, dass über 80-jährige Patient/-innen…]**	… to feel safe?<br><br>[… sich sicher fühlen?]<br><br>… feel recognized as meaningful human being?<br><br>[… sich als bedeutsame Menschen wahrgenommen fühlen?]<br><br>… be able to maintain autonomy and independence?<br><br>[… Autonomie und Unabhängigkeit bewahren können?]	"AW01 to AW04: Find it a big jump from the results of the survey to the question of ensuring these three aspects in the context of care. Would at least make the reference 'to be ensured in the context of care' even clearer. The three aspects otherwise sound very general and could also apply independently of care"<br><br>[„AW01 bis AW04: Finde es einen großen Sprung von den Ergebnissen der Untersuchung zur Frage der Gewährleistung dieser drei Aspekte im Rahmen von Versorgung. Würde zumindest den Bezug noch deutlicher machen ‚im Rahmen der Versorgung zu gewährleisten'. Die drei Aspekte klingen sonst sehr allgemein und könnten auch Versorgungs-unabhängig gelten"]<br><br>"I just noticed that the font size is relatively small. Is it my resolution	works as intended, uniform gendering with gender star<br><br>specific reference to care included in the item<br><br>Jump may be due to the fact that concepts are now "imported" from the Systematic Review<br><br>Unfortunately, font size cannot be changed in this item mode	In previous studies, we found that three basic motives shape the care wishes of people aged 80 and over: to feel safe, to feel like a meaningful human being, and to maintain autonomy and independence.<br>**What is the first thing you think of with regard to ensuring in care that patients aged 80 and over…**<br><br>[In vorangegangenen Untersuchungen haben wir herausgefunden, dass drei grundlegende Motive die Versorgungswünsche über 80-Jähriger prägen: sich sicher zu fühlen, als bedeutsame Menschen wahrgenommen zu werden sowie Autonomie und Unabhängigkeit zu bewahren.<br>**Woran denken Sie als Erstes, wenn es darum geht in der Versorgung zu gewährleisten,**

Original item	Answer options	Comments	Summary based on provided answers and comments	New item
		or can it be increased automatically?"<br><br>[„Mir fällt gerade auf, dass die Schriftgröße relativ klein ist. Liegt es an meiner Auflösung oder lässt sich diese automatisch erhöhen?“]		**dass über 80-jährige Patient*innen…]**
The survey is now almost over. **Reflecting briefly on your previous answers, has the corona pandemic changed your views and if so, how?**<br><br>[Die Befragung ist nun fast beendet. **Wenn Sie nochmal kurz über Ihre vorangegangenen Antworten nachdenken: Hat die Coronapandemie etwas an Ihrer Sichtweise geändert und wenn ja, was?]**	text field<br><br>[Textfeld]	none	works as intended	remains unchanged
**Is there anything else that you think is important for the care of patients aged 80 and over or that you would like to add?**<br><br>**[Gibt es noch etwas, dass Sie für die Versorgung über 80-jähriger Patient/-innen wichtig finden oder ergänzen möchten?]**	text field<br><br>[Textfeld]	none	works as intended, uniform gendering with gender star	**Is there anything else that you think is important for the care of patients aged 80 and over or that you would like to add?**<br><br>**[Gibt es noch etwas, dass Sie für die Versorgung über 80-jähriger Patient*innen wichtig finden oder ergänzen möchten?]**

Round 2

- Period: 15/09/2021-05/10/2021
- Participants: n = 6 (additionally two drop-outs), real-world sample, recruited via private/professional contacts, n = 3 of them from dentistry, 1x general/internal medicine, 1x otorhinolaryngology, 1x internal medicine
- 20-29 year old: n = 2, 30-39 years old: n = 4, 40-49 years old: n = 2; male: n = 1, female: n = 7
- Goal: Comprehensibility, applicability and acceptance in the real-world sample
- General findings:
    - Shorten or merge, also in the selection questions; there were some duplications, in addition, feedback came from two to three doctor networks that completion time is the critical factor
    - Separate reasons in a separate field not favourable, instead ask for description, encourage more writing
    - Shorten info text before open questions, do not encourage too many short answers
    - Particularly in the case of challenges with multiple enumeration, etc.: rather summarise and ask for description
    - Question about "what is (not) going well" does not work well and is sometimes perceived as a duplication; instead, integrate it into challenges
    - In the formulations, pay attention to the target group 80+ compared to younger groups
    - Instead of "care" use "practice, treatment" or similar.
    - Guide the participants through the questionnaire with "first part, second part, last part" so that they can keep going until the end

**Original item**	**Answer options**	**Comments**	**Summary based on provided answers and comments**	**New item**
**Please specify your age.**<br><br>**[Bitte geben Sie Ihr Alter an.]**	<29 years<br>[< 29 Jahre]	-	works as intended	remains unchanged
	20-29 years<br>[20-29 Jahre]			
	30-39 years<br>[30-39 Jahre]			
	40-49 years<br>[40-49 Jahre]			
	50-59 years<br>[50-59 Jahre]			
	60-69 years<br>[60-69 Jahre]			
	70-79 years<br>[70-79 Jahre]			
	>79 years<br>[> 79 Jahre]			

Original item	Answer options	Comments	Summary based on provided answers and comments	New item
**Please specify your gender.**<br><br>**[Bitte geben Sie Ihr Geschlecht an.]**	male<br>[Männlich]<br>female<br>[Weiblich]<br>diverse<br>[divers]<br>no specification<br>[Keine Angabe]	-	works as intended	remains unchanged
**Do you practice in employment or in your own practice?**<br><br>**[Praktizieren Sie in Anstellung oder in eigener Praxis?]**	In employment<br>[In Anstellung]<br>In my own practice<br>[In eigener Praxis]<br>Other (text field)<br>[Sonstiges (Textfeld)]	„You can't really call it really practice yet, but otherwise I wouldn't be the target group?"<br><br>[„So richtig praktizieren kann man das ja noch nicht nennen, aber sonst wäre ich auch nicht Zielgruppe?"]	Value of insight? Can be deleted for brevity, especially since there are some discrepancies or non-completion	Deleted
**How long have you been working in ambulatory healthcare?**<br>Please enter an integer.<br><br>**[Wie lange praktizieren Sie bereits in der ambulanten Versorgung?**<br>Bitte geben Sie eine ganze Zahl an.**]**	Numeric field<br><br>[Zahlenfeld]	-	works as intended	remains unchanged
**Please enter the first two digits of the postal code of the practice where you (mainly) work.**<br><br>**[Bitte geben Sie die ersten zwei Ziffern der Postleitzahl der Praxis an, in der Sie (hauptsächlich) arbeiten.]**	Numeric field<br><br>[Zahlenfeld]	-	works as intended	remains unchanged

Original item	Answer options	Comments	Summary based on provided answers and comments	New item
**What is the proportion of privately insured patients you treat?**<br>Please give an estimate.<br><br>**[Wie hoch ist der Anteil der von Ihnen behandelten Privatpatient*innen?**<br>Bitte geben Sie eine Schätzung an.]	< 10%<br>11-25%<br>26-40%<br>41-65%<br>66-80%<br>>80%	-	works as intended	remains unchanged
**In which area of healthcare are you mainly active?**<br><br>**[In welchem Bereich sind Sie hauptsächlich tätig?]**	Selection list<br><br>[Auswahlliste]	--	works as intended	remains unchanged
**Do you have a dental focus of activity or do you work in a dental specialist practice?**<br><br>**[Haben Sie einen zahnmedizinischen Tätigkeitsschwerpunkt oder arbeiten Sie in einer Zahnmedizinischen Facharztpraxis?]**	No<br><br>[Nein]<br>Yes → „Please indicate the focus of activity“, text field<br><br>[Ja → „Bitte geben Sie den Schwerpunkt an“, Textfeld]	-	works as intended	remains unchanged
**1) In your opinion, how much time was spent during your academic education to discuss the care of over 80-year-old or very old patients?**<br><br>**[1) Wie viel Zeit wurde Ihrer Einschätzung nach während des**	Little<br><br>[wenig]<br>Rather little<br><br>[eher wenig]<br>Rather much	-	Value of insight? Can be deleted for brevity, especially as there are some inconsistencies or omissions in the following similar question	Deleted, later more global question

Original item	Answer options	Comments	Summary based on provided answers and comments	New item
**Studiums aufgewendet, um die Versorgung über 80-jähriger bzw. hochaltriger Patient*innen zu thematisieren?]**	[eher viel]			
	Much<br><br>[viel]			
**2) In your opinion, how much time was spent during specialist training to address the issue of care for over 80-year-old or very old patients?<br><br>[2) Wie viel Zeit wurde Ihrer Einschätzung nach während der Facharztweiterbildung aufgewendet, um die Versorgung über 80-jähriger bzw. hochaltriger Patient*innen zu thematisieren?]**	Little<br><br>[wenig]	"Dentists do not necessarily have a specialist or study the specialist directly..."<br><br>[„Zahnmediziner haben ja nicht unbedingt einen Facharzt bzw. studieren ja direkt den Facharzt…“]<br><br>"I just related the last question to my situation, I'm not in further education..."<br><br>[„Die letzte Frage habe ich jetzt einfach auf meine Situation bezogen, ich bin ja nicht in der Weiterbildung…“]	Value of insight? Can be deleted for brevity, especially since there are some discrepancies or non-completion	Deleted, later more global question
	Rather little<br><br>[eher wenig]			
	Rather much<br><br>[eher viel]			
	Much<br><br>[viel]			
**How many further training courses have you attended in the last three years that dealt with the care of patients aged 80 and over?<br><br>[Wie viele Fortbildungen haben Sie darüber hinaus in den letzten drei Jahren besucht, die die Versorgung über 80-jähriger Patient*innen thematisierten?]**	< 3	"The KO02 training courses do not have "older people" as the main topic, but e.g. prosthetics. The topic then relates specifically to older people.“<br><br>[„Die Fortbildungen KO02 haben nicht als Hauptthema „Ältere“, aber z.B. Prothetik. Das Thema betrifft dann speziell Ältere.“]	Value of insight? Can be deleted for brevity	Deleted
	3-6			
	7-10			
	> 10			

**Original item**	**Answer options**	**Comments**	**Summary based on provided answers and comments**	**New item**
**In your opinion, are there enough training courses offered for you that deal with the care of patients aged 80 and over? [Werden Ihrer Meinung nach ausreichend Fortbildungen für Sie angeboten, die die Versorgung über 80-jähriger Patient/-innen thematisieren?]**	Yes<br><br>[Ja]<br>No<br><br>[Nein]	-	Value of insight? Can be deleted for of brevity, especially since there are some non-completions	Deleted, later more global question
**How high is the proportion of people aged 80 and over in your daily work?** Please provide an estimate.<br><br>**[Wie hoch schätzen Sie den Anteil der über 80-Jährigen in Ihrer täglichen Arbeit ein?** Bitte geben Sie eine Schätzung an.**]**	< 10%<br>11-25%<br>26-40%<br>41-65%<br>66-80%<br>>80%	"Actually, that's just a rough estimate, you don't always have that exact age in mind. You would have to look through the patient files."<br><br>[„Tatsächlich ist das nur eine grobe Schätzung, so genau hat man das Alter nicht immer im Kopf. Da müsste man die Patientenakten durchsehen."]	works as intended	remains unchanged
In the following, we would like to gain an insight into your work with patients aged 80 and over. For this purpose, we will now ask you a few open questions, which you can answer via a free text field. Please keep three things in mind:<br>1) You are free to answer the questions in bullet points or to describe them in more detail. However, whenever you can and would like to, we welcome brief explanations of your thoughts and reasons. This helps us to better understand your views.<br>2) "Care" is not only understood to mean medical aspects, but everything that is part of the services you provide on a daily or regular basis.<br>3) Please avoid giving personal data such as names.		-	Shorten and rather motivate to provide a a description; so far rather a suggestion to summarize	**In the following second part of the survey, we would like to gain an insight into your work with patients aged 80 and over.**<br>Please note: We would be pleased if you could describe your personal perception in a few sentences. Your explanations will help us to better understand your daily work and your views.

Original item	Answer options	Comments	Summary based on provided answers and comments	New item
[Im Folgenden möchten wir einen Einblick in Ihre Arbeit mit über 80-jährigen Patient*innen gewinnen. Dazu stellen wir Ihnen nun ein paar offene Fragen, die Sie über ein Freitextfeld beantworten können. Bitte beachten Sie dabei drei Dinge:<br>1) Es steht Ihnen frei, die Fragen stichpunktartig zu beantworten oder etwas ausführlicher zu beschreiben. Wann immer Sie können und möchten, freuen wir uns jedoch über kurze Ausführungen Ihrer Gedanken und Begründungen. Das hilft uns, Ihre Ansichten besser zu verstehen.<br>2) Unter „Versorgung“ werden nicht nur medizinische Aspekte verstanden, sondern alles, was für Sie zu Ihren täglich oder regelmäßig erbrachten Leistungen dazugehört.<br>3) Bitte vermeiden Sie die Angabe personenbezogener Daten wie bspw. Namen.]				Please avoid giving personal data (e.g., names) to ensure anonymity.<br><br>**[Im folgenden zweiten Teil der Befragung möchten wir einen Einblick in Ihre Arbeit mit über 80-jährigen Patient*innen gewinnen.**<br>Bitte beachten Sie: Wir freuen uns, wenn Sie uns Ihre persönliche Wahrnehmung in einigen Sätzen beschreiben. Ihre Erläuterungen helfen uns, Ihren Arbeitsalltag und Ihre Ansichten besser zu verstehen.<br>Bitte vermeiden Sie die Angabe personenbezogener Daten (bspw. Namen), um die Anonymität zu gewährleisten.]
**From your perspective, what are the special characteristics of patients over the age of 80?**<br>Think of typical situations, for example.<br><br>**[Welche besonderen Charakteristika kennzeichnen aus Ihrer Perspektive die über 80-jährigen Patient/-innen?**<br>Denken Sie hierbei zum Beispiel an typische Situationen.**]**	text field<br><br>[Textfeld]	**-**	Basically works as intended; rephrase and encourage more general description, integrating the following item	**How would you characterise your work with patients aged 80 and over?**<br>Please describe perceived particularities of this patient group or typical situations in contrast to working with younger patients.<br><br>**[Wie würden Sie Ihre Arbeit mit über 80-jährigen Patient*innen charakterisieren?**<br>Bitte beschreiben Sie wahrgenommene

**Original item**	**Answer options**	**Comments**	**Summary based on provided answers and comments**	**New item**
				Besonderheiten dieser Patientengruppe oder typische Situationen im Unterschied zur Arbeit mit jüngeren Patient*innen.]
**What is special about your work with patients aged 80 and over?**<br>Think, for example, of the special features that differ from working with younger patients.<br><br>**[Welche Besonderheiten gibt es in Ihrer Arbeit mit den über 80-jährigen Patient*innen?**<br>Denken Sie hierbei zum Beispiel an Besonderheiten im Unterschied zur Arbeit mit jüngeren Patient*innen.]	text field<br><br>[Textfeld]	„CV02 Unterschied zur Vorfrage?"<br>„Doppelt sich die Frage nicht mit der davor?"	Value of insight? Can be deleted for brevity, especially as there are some areas that have not been filled in and difficulties in understanding	Deleted/merged with previous question
**What is the first thing you think of regarding good care for people aged 80 and over and why do you think it is important?**<br><br>**[Woran denken Sie als Erstes, wenn es um eine gute Versorgung über 80-Jähriger geht und warum finden Sie das wichtig?]**	text field<br><br>[Textfeld]	-	Basically works as intended; rephrase and encourage more general description	**Please describe what, in your view, constitutes good healthcare in older age.**<br><br>**[Bitte beschreiben Sie, was aus Ihrer Sicht eine gute Gesundheitsversorgung im hohen Alter ausmacht.]**
**Which three aspects do you find challenging in working with people aged 80 and over?**	3x text field<br><br>[3x Textfeld]	"HC02 I don't understand the questions now."<br><br>[„HC02 Die Fragen verstehe ich jetzt nicht."]	Partial non-completion and difficulties in understanding; rewording to more global question, integrating subsequent items (duplicates); rephrasing to	**Please describe the challenges you encounter in working with people aged 80 and over.**

Original item	Answer options	Comments	Summary based on provided answers and comments	New item
**[Welche drei Aspekte empfinden Sie als herausfordernd in der Arbeit mit über 80-Jährigen?]**			encourage description (perhaps naming a number of aspects is both too challenging and too inviting to answer very succinctly)	**[Bitte beschreiben Sie, welche Herausforderungen Ihnen in der Arbeit mit über 80-Jährigen begegnen.]**
**Why do you find these aspects challenging?**<br><br>**[Warum empfinden Sie diese Aspekte als herausfordernd?]**	text field<br><br>[Textfeld]	-	See above, instead a global question about challenges	Deleted/merged with previous question
**Which three aspects do you find enriching in working with people aged 80 and over?**<br><br>**[Welche drei Aspekte empfinden Sie als bereichernd in der Arbeit mit über 80-Jährigen?]**	3x text field<br><br>[3x Textfeld]	"I really don't understand these questions. All patients are special..."<br><br>[„Diese Fragen verstehe ich echt nicht. Alle Patienten sind speziell…“]<br><br>"I can't think of anything right now. Of course, all this is not just bad and difficult with the older ones, but I wouldn't call it "enriching" either.“<br><br>[„Hier fällt mir jetzt so schnell nichts ein. Natürlich ist das alles nicht nur schlecht und schwierig mit den Älteren, aber „bereichernd“ würde ich das jetzt auch nicht nennen.“]	This question works very poorly; delete for brevity and instead ask for supporting factors in the specific question about factors that prevent good care	Deleted, instead an additional item about support factors later

Original item	Answer options	Comments	Summary based on provided answers and comments	New item
**Why do you find these aspects enriching?**<br><br>**[Warum empfinden Sie diese Aspekte als bereichernd?]**	text field<br><br>[Textfeld]	-	This question works poorly; delete for brevity and instead ask for supporting factors in the specific question about factors that prevent good care	Deleted, instead an additional question about support factors later
**What is going (rather) well in your work with people aged 80 and over and why?**<br><br>**[Was läuft in Ihrer Arbeit mit den über 80-Jährigen (eher) gut und warum?]**	text field<br><br>[Textfeld]	"What exactly do you want to know? Do you mean in comparison to other age groups?"<br><br>[„Was wollen Sie konkret wissen? Meinen Sie im Vergleich zu anderen Altersgruppen?"]	This question works poorly; delete for brevity and instead ask for supporting factors in the specific question about factors that prevent good care	Deleted, instead an additional item about support factors later
**What is going (rather) bad in your work with people aged 80 and over and why?**<br><br>**[Was läuft in Ihrer Arbeit mit den über 80-Jährigen (eher) schlecht und warum?]**	text field<br><br>[Textfeld]	"It's not really clear to me what is meant here. What is difficult has already been discussed before."<br><br>[„Hier ist mir nicht so klar, was gemeint ist. Was schwierig ist, wurde ja auch vorher schon thematisiert."]	see above, instead a global question about challenges	deleted/merged with question about challenges
**What measures do you wish for to improve care for people aged 80 and over and why?**<br>This does not only have to address the patients directly, but also think about what would be helpful for you in your work and role as a medical professional.<br><br>**[Welche Maßnahmen wünschen Sie sich zur Verbesserung der**	text field<br><br>[Textfeld]	-	Partially not answered, reason unclear, possibly lack of motivation or no ideas; in general, however, the question seems to be working as intended; adaptation of the wording according to the general results, e.g., concept of care	**What measures would you welcome for the future design or improvement of ambulatory healthcare with regard to older age?**<br>This does not only have to address the patients directly, but also think about what would be helpful for you in your work and role as a medical professional.

Original item	Answer options	Comments	Summary based on provided answers and comments	New item
**Versorgung über 80-Jähriger und warum?**<br>Dabei muss es nicht nur direkt um die Patient*innen gehen, denken Sie auch daran, was für Sie in Ihrer Arbeit und Rolle als Mediziner*in hilfreich wäre.]				**[Welche Maßnahmen würden Sie zur zukünftigen Gestaltung oder Verbesserung der ambulanten Gesundheitsversorgung im Hinblick auf das hohe Alter begrüßen?**<br>Dabei muss es nicht nur direkt um die Patient*innen gehen, denken Sie auch daran, was für Sie in Ihrer Arbeit und Rolle als Mediziner*in hilfreich wäre.]
**Do you feel well educated or trained to care for patients aged 80 and over?**<br><br>**[Fühlen Sie sich gut für die Versorgung über 80-jähriger Patient*innen aus- bzw. fortgebildet?]**	No<br>[Nein]<br>rather no<br>[eher nein]<br>rather yes<br>[eher ja]<br>yes<br>[ja]	-	Works as intended, rephrasing to incorporate previous deleted questions; flip the scale so there is no negative priming	Combined from all education/training questions: **In general, do you feel well and sufficiently educated and trained for working with over 80s?**<br>Turn scale<br><br>[Zusammengeführt aus allen Aus-/Fortbildungsfragen: **Fühlen Sie sich im Allgemeinen gut und ausreichend für die Arbeit mit über 80-Jährigen aus- und fortgebildet?**<br>Skala umdrehen]
**In how many cases can you care for patients aged 80 and over in a way that you personally think is right and important?**<br>Please provide an estimate.	<25%<br>25-49%<br>50-75%<br>> 75 %	-	Partially not answered, reason for this is unclear, possibly declining motivation or no estimate possible, but basically works as intended	remains unchanged

Original item	Answer options	Comments	Summary based on provided answers and comments	New item
**[In wie vielen Fällen können sie über 80-jährige Patient*innen so versorgen, wie Sie persönlich es für richtig und wichtig halten?**<br>Bitte geben Sie eine Schätzung an.]				
**If you cannot care for patients aged 80 and over in the way you personally think is right and important – what is the reason?**<br>text field<br><br>**[Wenn Sie über 80-jährige Patient*innen nicht so versorgen können, wie Sie persönlich es für richtig und wichtig halten – woran liegt das?**<br>(Textfeld)]	text field<br><br>[Textfeld]	-	works as intended	Same, additional question about support instead of "what is going well" above.<br>**What supports you in treating patients aged 80 and over in the way you personally think is right and important?**<br><br>[Gleichbleibend, zusätzlich Frage nach Unterstützung statt „was läuft gut" oben<br>**Was unterstützt Sie dabei, über 80-Jährige so zu behandeln, wie Sie persönlich es für richtig und wichtig halten?]**
In previous studies, we found that three basic motives shape the care wishes of people aged 80 and over: to feel safe, to feel like a meaningful human being, and to maintain autonomy and independence.<br>**What is the first thing you think of with regard to ensuring in**	… to feel safe?<br><br>[… sich sicher fühlen?]	-	Partially not answered, reason is unclear, possibly lack of motivation or no ideas, but basically works as intended	remains unchanged
	… feel recognized as meaningful human being?	-	Partially not answered, reason is unclear, possibly lack of motivation or no ideas, but basically works as intended	

Original item	Answer options	Comments	Summary based on provided answers and comments	New item
**care that patients aged 80 and over…**<br><br>[In vorangegangenen Untersuchungen haben wir herausgefunden, dass drei grundlegende Motive die Versorgungswünsche über 80-Jähriger prägen: sich sicher zu fühlen, als bedeutsame Menschen wahrgenommen zu werden sowie Autonomie und Unabhängigkeit zu bewahren.<br>**Woran denken Sie als Erstes, wenn es darum geht in der Versorgung zu gewährleisten, dass über 80-jährige Patient*innen…]**	[… sich als bedeutsame Menschen wahrgenommen fühlen?]			
	… be able to maintain autonomy and independence?<br><br>[… Autonomie und Unabhängigkeit bewahren können?]	-	Partially not answered, reason is unclear, possibly lack of motivation or no ideas, but basically works as intended	
The survey is now almost over. **Reflecting briefly on your previous answers, has the corona pandemic changed your views and if so, how?**<br><br>[Die Befragung ist nun fast beendet.<br>**Wenn Sie nochmal kurz über Ihre vorangegangenen Antworten nachdenken: Hat die Coronapandemie etwas an Ihrer Sichtweise geändert und wenn ja, was?]**	text field<br><br>[Textfeld]	-	No results that seem to influence the survey, moreover, much more time has now passed with the pandemic, experience from interviews is also that this "control question" is not of much use; therefore, also deleted for brevity	deleted
**Is there anything else that you think is important for the care**	text field<br><br>[Textfeld]	-	Works as intended, shorten the wording a bit and refer to the last item	**Is there anything else you would like to add to this topic?**

Original item	Answer options	Comments	Summary based on provided answers and comments	New item
**of patients over 80 or that you would like to add?**<br><br>**[Gibt es noch etwas, dass Sie für die Versorgung über 80-jähriger Patient*innen wichtig finden oder ergänzen möchten?]**				**[Gibt es noch etwas, dass Sie abschließend zu diesem Thema ergänzen möchten?]**
**Was this survey easy to understand and ran smoothly for you?** If you have any further comments on this survey that you would like us to take into account in the revision, please feel free to enter them here.<br><br>**[War diese Befragung für Sie gut verständlich und reibungslos durchführbar?** Wenn Sie noch weitere Anmerkungen zu dieser Befragung haben, die wir in der Überarbeitung berücksichtigen sollen, können Sie dies gerne hier eintragen.]		"See Notes"<br><br>[„Siehe Anmerkungen“]<br>"Sometimes the questions seem very similar to me, I have to say."<br><br>[„Manchmal kommen mir die Fragen sehr ähnlich vor, muss ich sagen.“]<br><br>"Yes"<br><br>[„ja“]<br><br>"See Notes"<br><br>[„siehe Anmerkungen“]<br>-,<br>-,<br>-,<br>-	Above all, merge and shorten duplicate and similar questions	

Round 3 (final survey)

- Period: 06/10/2021-15/10/2021
- Participants: n = 7, real-world sample (additionally two drop-outs), of which n = 4 dentistry, 1x nuclear medicine, 1x human genetics, 1x dermatology
- 20-29 years old: n = 1, 30-39 years old: n = 4, 40-49 years old: n = 2; male: n = 1, female: n = 6
- Goal: Comprehensibility, applicability and acceptance in the real-world sample
- General findings:
    - The items work as intended
    - There were only a few comments, but after discussion in the research team, they were not relevant (for the aim of the study)
    - The survey draft is thus considered confirmed, no more changes will be made, the survey is ready for data collection

Original item	Answer options	Comments	Summary based on provided answers and comments	New item
**Please specify your age.**<br><br>**[Bitte geben Sie Ihr Alter an.]**	<29 years<br>[< 29 Jahre]	-	works as intended	remains unchanged
	20-29 years<br>[20-29 Jahre]			
	30-39 years<br>[30-39 Jahre]			
	40-49 years<br>[40-49 Jahre]			
	50-59 years<br>[50-59 Jahre]			
	60-69 years<br>[60-69 Jahre]			
	70-79 years<br>[70-79 Jahre]			
	>79 years<br>[> 79 Jahre]			
**Please specify your gender.**<br><br>**[Bitte geben Sie Ihr Geschlecht an.]**	male<br>[Männlich]	-	works as intended	remains unchanged
	female<br>[Weiblich]			
	diverse<br>[divers]			
	no specification			

**Original item**	**Answer options**	**Comments**	**Summary based on provided answers and comments**	**New item**
	[Keine Angabe]			
**How long have you been working in ambulatory healthcare?**<br>Please enter an integer.<br><br>**[Wie lange praktizieren Sie bereits in der ambulanten Versorgung?**<br>Bitte geben Sie eine ganze Zahl an.**]**	Numeric field<br><br>[Zahlenfeld]	"The overall ambulatory activity is longer than the specialized activity. Is that relevant? I can't give any information here about how long I've been working in a "specialized" manner."<br><br>[„Die insgesamt ambulante Tätigkeit ist länger als die spezialisierte Tätigkeit. Ist das relevant? Ich kann hier keine Angaben dazu machen, wie lange ich „spezialisiert" arbeite."]	Works as intended, the annotation is irrelevant	remains unchanged
**Please enter the first two digits of the postal code of the practice where you (mainly) work.**<br><br>**[Bitte geben Sie die ersten zwei Ziffern der Postleitzahl der Praxis an, in der Sie (hauptsächlich) arbeiten.]**	Numeric field<br><br>[Zahlenfeld]	-	works as intended	remains unchanged
**In which area of healthcare are you mainly active?**<br><br>**[In welchem Bereich sind Sie hauptsächlich tätig?]**	Selection list<br><br>[Auswahlliste]	-	works as intended	remains unchanged
**Do you have a dental focus of activity or do you work in a dental specialist practice?**	No<br><br>[Nein]	-	works as intended	remains unchanged

Original item	Answer options	Comments	Summary based on provided answers and comments	New item
**[Haben Sie einen zahnmedizinischen Tätigkeitsschwerpunkt oder arbeiten Sie in einer Zahnmedizinischen Facharztpraxis?]**	Yes → „Please indicate the focus of activity", text field<br><br>[Ja → „Bitte geben Sie den Schwerpunkt an", Textfeld]			
**What is the proportion of privately insured patients you treat?** Please give an estimate.<br><br>**[Wie hoch ist der Anteil der von Ihnen behandelten Privatpatient*innen?** Bitte geben Sie eine Schätzung an.]	< 10%<br>11-25%<br>26-40%<br>41-65%<br>66-80%<br>>80%	-	works as intended	remains unchanged
**How high is the proportion of people aged 80 and over in your daily work?** Please provide an estimate.<br><br>**[Wie hoch schätzen Sie den Anteil der über 80-Jährigen in Ihrer täglichen Arbeit ein?** Bitte geben Sie eine Schätzung an.]	< 10%<br>11-25%<br>26-40%<br>41-65%<br>66-80%<br>>80%	-	works as intended	remains unchanged
**Do you feel well educated or trained to care for over 80-year-old patients?**<br><br>**[Fühlen Sie sich gut für die Versorgung über 80-jähriger Patient*innen aus- bzw. fortgebildet?]**	Yes<br>[Ja]<br>Rather yes<br>[eher ja]<br>Rather no<br>[Eher nein]<br>No<br>[nein]	-	works as intended	remains unchanged

Original item	Answer options	Comments	Summary based on provided answers and comments	New item
**In the following second part of the survey, we would like to gain an insight into your work with patients aged 80 and over.**<br>Please note: We would be pleased if you could describe your personal perception in a few sentences. Your explanations will help us to better understand your daily work and your views.<br>Please avoid giving personal data (e.g., names) to ensure anonymity.<br><br>**[Im folgenden zweiten Teil der Befragung möchten wir einen Einblick in Ihre Arbeit mit über 80-jährigen Patient*innen gewinnen.**<br>Bitte beachten Sie: Wir freuen uns, wenn Sie uns Ihre persönliche Wahrnehmung in einigen Sätzen beschreiben. Ihre Erläuterungen helfen uns, Ihren Arbeitsalltag und Ihre Ansichten besser zu verstehen.<br>Bitte vermeiden Sie die Angabe personenbezogener Daten (bspw. Namen), um die Anonymität zu gewährleisten.]		-	works as intended	remains unchanged
**How would you characterise your work with patients aged 80 and over?**<br>Please describe perceived particularities of this patient group or typical situations in contrast to working with younger patients.<br><br>**[Wie würden Sie Ihre Arbeit mit über 80-jährigen Patient*innen charakterisieren?**<br>Bitte beschreiben Sie wahrgenommene Besonderheiten dieser Patientengruppe oder typische Situationen im	text field<br><br>[Textfeld]	-	works as intended	remains unchanged

Original item	Answer options	Comments	Summary based on provided answers and comments	New item
Unterschied zur Arbeit mit jüngeren Patient*innen.]				
**Please describe what, in your view, constitutes good healthcare in older age.**<br><br>**[Bitte beschreiben Sie, was aus Ihrer Sicht eine gute Gesundheitsversorgung im hohen Alter ausmacht.]**	text field<br><br>[Textfeld]	"CV03 - does the question refer to my field (e.g. human genetics) or to the Germany-wide health system?"<br><br>[„CV03 – bezieht sich die Frage auf meinen Bereich (z.B. Humangenetik) oder auf das deutschlandweite Gesundheitssystem?“]	Works as intended; no more precise specification, because depending on the heterogeneity of the final sample, conclusions would otherwise be too difficult, and this comment is made for the first time	remains unchanged
**Please describe the challenges you encounter in working with people aged 80 and over.**<br><br>**[Bitte beschreiben Sie, welche Herausforderungen Ihnen in der Arbeit mit über 80-Jährigen begegnen.]**	text field<br><br>[Textfeld]	“I already answered these aspects in the penultimate question. For me, special features are also the challenges.”<br><br>[„Diese Aspekte habe ich bereits bei der vorletzten Frage beantwortet. Besonderheiten sind für mich auch die Herausforderungen.“]<br><br>"HO02 should come before CV03 in my opinion" [challenges before ideas of good care]<br><br>[„HO02 sollte meiner Meinung nach vor CV03 kommen“ [Herausforderungen vor	Works as intended; no more precise specification, because depending on the heterogeneity of the final sample, conclusions would otherwise be too difficult, and this comment is made for the first time; conceptually, challenges are to be separated from generally perceived peculiarities; no conversion, otherwise framing of challenges (negative) would be possible	remains unchanged

Original item	Answer options	Comments	Summary based on provided answers and comments	New item
		Vorstellungen von guter Versorgung]]<br><br>"HO02 - does the question relate to my area (e.g. human genetics) or to the German health system?"<br><br>[„HO02 – bezieht sich die Frage auf meinen Bereich (z.B. Humangenetik) oder auf das deutschlandweite Gesundheitssystem?“]		
This is the last part of the questionnaire.<br>**In how many cases can you care for patients aged 80 and over in a way that you personally think is right and important?**<br>Please provide an estimate.<br><br>[Es folgt der letzte Teil der Befragung.<br>**In wie vielen Fällen können Sie über 80-jährige Patient*innen so versorgen, wie Sie persönlich es für richtig und wichtig halten?**<br>Bitte geben Sie eine Schätzung an.]	<25%<br>25-49%<br>50-75%<br>> 75 %	"Wouldn't the question be more whether I can care for the patients in the way THEY think is right and important? Topic: Patient-centered (dental) medicine"<br><br>[„Wäre die Frage nicht eher, ob ich die Patient:innen so versorgen kann, wie DIESE es für richtig und wichtig halten? Thema: Patientenzentrierte (Zahn-)Medizin“]<br><br>"AP02 - does the question relate to my area (e.g. human genetics) or to the German health system?"<br><br>[„AP02 – bezieht sich die Frage auf meinen Bereich	Works as intended; no more precise specification, because depending on the heterogeneity of the final sample, conclusions would otherwise be too difficult, and this comment is made for the first time	remains unchanged

Original item	Answer options	Comments	Summary based on provided answers and comments	New item
		(z.B. Humangenetik) oder auf das deutschlandweite Gesundheitssystem?“]		
**If you cannot care for patients aged 80 and over in the way you personally think is right and important – what is the reason?**<br><br>**[Wenn Sie über 80-jährige Patient*innen nicht so versorgen können, wie Sie persönlich es für richtig und wichtig halten – woran liegt das?]**	text field<br><br>[Textfeld]	"See note on previous question. Regarding AP02 and AP03: The question should rather be whether I treated the patients correctly at their discretion. Topic patient-centered (dental) medicine. The way I think it is right and/or important is rather secondary.”<br><br>[„Siehe Anmerkung zur vorherigen Frage. Zu AP02 und AP03: Die Frage sollte eher lauten, ich die Patient:innen nach ihrem Ermessen richtig behandelt habe. Thema Patientenzentrierte (Zahn-)Medizin. So wie ich es für richtig und oder wichtig halte ist da eher nebensächlich.“]<br><br>"AP03 - does the question relate to my area (e.g. human genetics) or to the German health system?"<br><br>[„AP03 – bezieht sich die Frage auf meinen Bereich (z.B. Humangenetik) oder	Works as intended; no more precise specification, because depending on the heterogeneity of the final sample, conclusions would otherwise be too difficult, and this comment is made for the first time	remains unchanged

Original item	Answer options	Comments	Summary based on provided answers and comments	New item
		auf das deutschlandweite Gesundheitssystem?“]		
**What supports you in treating people aged 80 and over in the way you personally think is right and important?**<br><br>**[Was unterstützt Sie dabei, über 80-Jährige so zu behandeln, wie Sie persönlich es für richtig und wichtig halten?]**	text field<br><br>[Textfeld]	"AP05 - does the question relate to my area (e.g. human genetics) or to the German health system?"<br><br>[„AP05 – bezieht sich die Frage auf meinen Bereich (z.B. Humangenetik) oder auf das deutschlandweite Gesundheitssystem?“]	Works as intended; no more precise specification, because depending on the heterogeneity of the final sample, conclusions would otherwise be too difficult, and this comment is made for the first time	remains unchanged
In previous studies, we found that three basic motives shape the care wishes of people aged 80 and over: to feel safe, to feel like a meaningful human being, and to maintain autonomy and independence.<br>**What is the first thing you think of with regard to ensuring in care that patients aged 80 and over…**<br><br>[In vorangegangenen Untersuchungen haben wir herausgefunden, dass die folgenden drei Aspekte für über 80-Jährige relevant sind.<br>**Wodurch glauben Sie, können Sie gewährleisten, dass über 80-jährige Patient*innen…]**	… to feel safe?<br><br>[… sich sicher fühlen?]<br><br>… feel recognized as meaningful human being?<br><br>[… sich als bedeutsame Menschen wahrgenommen fühlen?]<br><br>… be able to maintain autonomy and independence?<br><br>[… Autonomie und Unabhängigkeit bewahren können?]	"AW01 Does the question relate to my area (e.g. human genetics) or to the German health system?"<br><br>[„AW01 bezieht sich die Frage auf meinen Bereich (z.B. Humangenetik) oder auf das deutschlandweite Gesundheitssystem?“]	Works as intended; no more precise specification, because depending on the heterogeneity of the final sample, conclusions would otherwise be too difficult, and this comment is made for the first time	remains unchanged

Original item	Answer options	Comments	Summary based on provided answers and comments	New item
**What role does relationship-building with patients oaged 80 and over play for you?**<br>Please describe how you perceive your relationship with patients over 80 and what relevance you attribute to the relationship level.<br><br>**[Welche Rolle spielt die Beziehungsgestaltung mit über 80-jährigen Patient*innen für Sie?**<br>Bitte beschreiben Sie, wie Sie Ihre Beziehung zu über 80-jährigen Patient*innen wahrnehmen und welche Relevanz Sie der Beziehungsebene zuschreiben.]	text field<br><br>[Textfeld]	"difficult question AW05, answer options would be better".<br><br>[„schwierige Frage AW05, Antwortmöglichkeiten wären besser“]<br><br>"AW05 - does the question relate to my area (e.g. human genetics) or to the German health system? AW05 puhhh ... you could write a lot here and I'm just losing interest. With such a question, I would prefer options (multiple answers) and an additional point for your own comments""<br><br>[„AW05 – bezieht sich die Frage auf meinen Bereich (z.B. Humangenetik) oder auf das deutschlandweite Gesundheitssystem? AW05 puhhh … hier könnte man viel schreiben und ich verliere gerade die Lust. Bei so einer Frage würde ich Auswahlmöglichkeiten (Mehrfachnennung) bevorzugen und einen zusätzlichen Punkt für eigene Kommentare“]	Was tested for round 3 because the topic became relevant again in the course of the analysis of a parallel interview study<br>3x not answered, criticism of the question (lengthy answer), possibly the motivation tilts at this point → therefore, rather delete, seems too complex or participants hardly inclined to answer this question in writing	Deleted

Original item	Answer options	Comments	Summary based on provided answers and comments	New item
This is the end of the survey. **What measures would you welcome for the future design or improvement of ambulatory healthcare with regard to older age?** This does not only have to address the patients directly, but also think about what would be helpful for you in your work and role as a medical professional.<br><br>[Zum Abschluss: **Welche Maßnahmen würden Sie zur zukünftigen Gestaltung oder Verbesserung der ambulanten Gesundheitsversorgung im Hinblick auf das hohe Alter begrüßen?** Dabei muss es nicht nur direkt um die Patient*innen gehen, denken Sie auch daran, was für Sie in Ihrer Arbeit und Rolle als Mediziner*in hilfreich wäre.]	text field<br><br>[Textfeld]	"MV01 - see also question AW05 ... you could write a lot here and I'm just losing interest. With such a question, I would prefer options (multiple answers) and an additional point for your own comments"<br><br>[„MV01 – siehe auch Frage AW05 … hier könnte man viel schreiben und ich verliere gerade die Lust. Bei so einer Frage würde ich Auswahlmöglichkeiten (Mehrfachnennung) bevorzugen und einen zusätzlichen Punkt für eigene Kommentare“]	Generally works as intended by deleting the previous item maintenance of motivation level	remains unchanged
**Is there anything else you would like to add to this topic?**<br><br>**[Gibt es noch etwas, dass Sie abschließend zu diesem Thema ergänzen möchten?]**	text field<br><br>[Textfeld]	-	works as intended	remains unchanged
**Was this survey easy to understand and ran smoothly for you?** If you have any further		-,<br>-,<br>„yes“	After this round, the survey appears operational, and the pretest is ended	

Original item	Answer options	Comments	Summary based on provided answers and comments	New item
comments on this survey that you would like us to take into account in the revision, please feel free to enter them here.<br><br>**[War diese Befragung für Sie gut verständlich und reibungslos durchführbar?** Wenn Sie noch weitere Anmerkungen zu dieser Befragung haben, die wir in der Überarbeitung berücksichtigen sollen, können Sie dies gerne hier eintragen.]		[„Jipp."]<br><br>"Yes, except for small things, see notes."<br><br>[„Ja bis auf Kleinigkeiten, siehe Anmerkungen."]<br><br>„yes"<br>[„Ja."]<br><br>"see comments/notes but otherwise it was understandable"<br><br>[„siehe Kommentare/Anmerkungen aber ansonsten war es verständlich"]<br>„yes"<br>[„Ja."]		
