# Supplementary material for: Providing ambulatory healthcare for people aged 80 and over: Views and perspectives of physicians and dentists from a qualitative survey
Source: PLoS One. 2022 Aug 15;17(8):e0272866. doi: 10.1371/journal.pone.0272866 (PMC9377615; doi:10.1371/journal.pone.0272866)
Supplement: S2 Appendix — (PDF) [file pone.0272866.s002.pdf]

S2 Appendix: Full codebook

GP, general practitioner; S, specialist; D, dentist

Theme	Subthemes/ codes	No of cases coded	Group A (physicians)		No of cases coded	Group B (dentists)	
Charac-teristics of working with people aged 80 and over			Illustrative interview passage (English translation)	Illustrative interview passage (German original)		Illustrative interview passage (English translation)	Illustrative interview passage (German original)
	Role of life and experiences	2	“This group of patients is very pragmatic, because they experienced several deprivations and losses in life.” (GP-198, §3)	„Diese Patientengruppe ist sehr pragmatisch, da sie im Leben oftmals viele Entbehrungen und Verluste erlebten.“ (GP-198, §3)	1	„Access to other emotional past, life and dental experience.” (D-451, §5)	„Zugriff auf andere emotionale Vergangenheit, Leben und zahnärztliche Erfahrung“ (D-451, §5)
	Positive attributes	8	"You get more gratitude. It's more fun." (GP-253, §4)	„Man erhält mehr Dankbarkeit. Es macht mehr Spaß.“ (GP-253, §4)	6	"Often friendly, sympathetic and above all respectful patients.“ (D-189, §9)	„Oft freundliche, sympathische und vor allem respektvolle Patienten“ (D-189, §9)
	Challenging attributes	15	"No one has a clue, but everyone has an opinion. Glasses not with me. Hearing aid not with them. Medication list not there." (S-217, §3-6)	„Keiner hat eine Ahnung, aber jeder hat eine Meinung. Brille nicht mit. Hörgerät nicht mit. Medikamentenliste nicht mit.“ (S-217, §3-6)	4	„Lack of/difficulty in adapting to innovations“ (D-189, §3)	„Fehlende/Schwerfällige Anpassungsfähigkeit an Neuerungen“ (D-189, §3)
	Complexity due to multimorbidity and polypharmacy	12	"Often there is a multi-drug situation, with more than 10 active substances." (S-226, §9)	„Häufig liegt eine multi-drug-Situation vor, mit mehr als 10 Wirkstoffen.“ (S-226, §9)	7	„There are often limitations in treatment due to pre-existing conditions. Drug interactions." (D-399, §4)	„Häufig gibt es Einschränkungen in der Behandlung durch Vorerkrankungen. Wechselwirkungen mit Medikamenten.“ (D-399, §4)

Theme	Subthemes/ codes	No of cases coded	Group A (physicians)		No of cases coded	Group B (dentists)	
	Physical and cognitive limitations	19	"It is more common that communication problems occur due to age-related hearing loss." (GP-219, §3)	„Es kommt eher vor, dass Kommunikationsprobleme aufgrund der Altersschwerhörigkeit vorkommen.“ (GP-219, §3)	20	"Almost every treatment is more difficult and strenuous. Limiting factors include physical limitations and associated positioning difficulties." (D-420, §5)	„Fast jede Behandlung ist schwieriger und anstrengender. Limitierende Faktoren sind u.a. körperliche Einschränkungen und damit verbundene Lagerungsschwierigkeiten.“ (D-420, §5)
	Heterogenous appearance	3	"Some are mentally fit, self-determined and demanding, others very insecure and tardy". (S-226, §4)	„Manche sind mental fit, selbstbestimmt und fordernd, andere sehr unsicher und verlangsamt.“ (S-226, §4)	3	„Wide range: from top fit to [care] degree 5.“ (D-234, §3)	„Große Bandbreite: von topfit bis [Pflege-]Grad 5.“ (D-234, §3)
	Higher need for care	0	-	-	3	"More periodontal diseases" (D-416, §4)	„Mehr parodontale Erkrankungen“ (D-416, §4)
	Perspective of people aged 80 and over regarding their health and care	7	"A big help would be more realism in patients who perceive themselves through 'rose-coloured glasses'." (GP-353, §13)	„Eine große Hilfe wäre mehr Realismus bei Patienten, die sich selbst durch die "rosa-rote Brille" wahrnehmen.“ (GP-353, §13)	6	"You are often confronted with the statement: it's not worth it at my age anymore". (D-398, §3)	„Man sieht sich oft mit der Aussage konfrontiert: das lohnt sich in meinem Alter doch nicht mehr.“ (D-398, §3)
	Paternalistic orientation	4	"Paternalistic, which is also readily accepted" (GP-353, §3).	„Paternalistisch, was auch gern angenommen wird“ (GP-353, §3)	0	-	-
	Discernment and compliance	3	"Many patients are less critical and do what they are advised". (GP-202, §6)	„Viele Patienten sind weniger kritisch und tun, was man ihnen empfiehlt.“ (GP-202, §6)	4	"Compliance is very limited". (D-384, §3)	„Die Compliance ist sehr eingeschränkt.“ (D-384, §3)
	Relationship-building and trust	4	„More trust“ (S-231, §3)	„Mehr Vertrauen“ (S-231, §3)	3	"Nevertheless, this group of patients is good to care of. Especially when you have known the people for many	„Trotzdem ist diese Patientengruppe gut zu betreuen. Gerade, wenn man die Menschen seit vielen Jahren kennt. Sie

Theme	Subthemes/ codes	No of cases coded	Group A (physicians)		No of cases coded	Group B (dentists)	
						years. They are very grateful." (D-521, §5)	sind sehr dankbar." (D-521, §5)
	Speed and time	20	"Complex cases and a slow pace (physically, but also mentally) with a greater expenditure of time" (GP-244, §4).	„Komplexe Fälle und eine geringe Geschwindigkeit (körperlich, aber auch mental) mit größerem Zeitaufwand" (GP-244, §4)	11	"In contrast to the rest of the pool of patients, it is more time-consuming and psychologically more difficult to deal with." (D-374, §3)	„Im Gegensatz zu dem Rest des Patientenpools ist es ein zeitlich aufwändigerer und psychologisch schwierigerer Umgang." (D-374, §3)
	Continuity and control	2	„Care over years, then sudden changes (cognition(mobility); good doctor-patient-relationship, long-lasting relationship of trust; support over years through different crises of life and health." (GP-518, §3)	„Betreuung über Jahre, dann oft plötzliche Veränderungen (Kognition/Mobilität); gute Arzt-Patienten-Bindung, lange dauerndes Vertrauensverhältnis; Begleitung über die Jahre durch verschiedene Lebens-/ Gesundheits-krisen" (GP-518, §3)	2	"The patients have usually been in treatment continuously for decades. There are regular check-ups and prevention. Great trust." (D-362, §3)	„Die Patienten sind in der Regel schon Jahrzehnte kontinuierlich in Behandlung. Es gibt regelmäßige Untersuchungen und Prävention. Großes Vertrauen." (D-362, §3)
	Need for support	2	"They are often lonely, need tips and help for everyday questions (e.g. how and where can I meet people or get help? Who can help me programme my television? They can't find their way around the internet, don't know how to apply for care levels, can't find telephone numbers for health insurance)." (GP-506, §3)	„Sind oft einsam, brauchen Tipps und Hilfe für Alltagsfragen (z. Bsp. wie und wo kann ich Menschen kennenlernen oder Hilfe bekommen? Wer hilft mir meinen Fernseher zu programmieren? Sie finden sich im Internet nicht zurecht, wissen nicht wie die Pflegstufen zu beantragen sind, finden Telefonnummern für die Krankenkasse nicht.)." (GP-506, §3)	1	"Prophylaxis support, as it can often no longer be carried out on its own in everyday life". (D-383, §3)	„Prophylaxeunterstützung, da sie allein häufig nicht mehr durchgeführt werden kann im Alltag." (D-383, §3)

Theme	Subthemes/ codes	No of cases coded	Group A (physicians)		No of cases coded	Group B (dentists)	
	Additional stakeholders and actors	7	"Often co-counselling of relatives" (S-232, §3)	„Häufig Mitberatung von Angehörigen“ (S-232, §3)	6	"The question quickly arises in the back of my mind: do I have to discuss all relevant issues with the patient only or with other people (relatives/carers etc.)?" (D-371, §4)	„Es stellt sich schnell die Frage im Hinterkopf: muss ich alle relevanten Fragen nur mit dem Patienten oder mit weiteren Personen (Angehörigen/ Betreuern etc.) besprechen.“ (D-371, §4)
	Communication	9	"People over 80 are clearly slower in their movements, understand less well, can't remember information from me more often, are sometimes demented." (S-372, §3)	„Über 80 Jährige sind deutlich langsamer in ihren Bewegungen, verstehen schlechter, können sich öfter die Informationen von mir nicht merken, sind manchmal auch dement.“ (S-372, §3)	10	"Hearing loss and dementia sometimes make it difficult to communicate with the patient and that makes it difficult to cooperate. But in general, the treatment is similar to the treatment of small children." (D-433, §3)	„Schwerhörigkeit und Demenz machen es manchmal schwer, mit dem Patienten zu kommunizieren und das erschwert die Mitarbeit. Aber im Prinzip ist die Behandlung ähnlich zu der Behandlung kleiner Kinder.“ (D-433, §3)
	Treatment concepts	6	"Often solving utility/everyday problems" (GP-253, §5).	„Oftmals Lösen von Versorgungs-/Alltagsproblemen“ (GP-253, §5)	11	"Age-appropriate therapy concepts, duration, sustainability, necessity, meaningfulness, quality of life" (D-451, §6)	„Altersgerechte Therapiekonzepte, Laufzeit, Nachhaltigkeit, Notwendigkeit, Sinnigkeit, Lebensqualität“ (D-451, §6)
	Structural and organizational specific features	4	"Often we have to take care of things that are more organisational, like contacting and planning care services, calling nursing homes, informing social agencies in the city, etc." (GP-506, §3)	„Oft müssen wir solche Dinge, die eher organisatorisch sind übernehmen, wie Pflegedienste kontaktieren und planen, Altenheime anrufen, Sozialdienste der Stadt informieren etc.“ (GP-506, §3)	5	"I look after a nursing home and visit it regularly. Working with the very elderly is more time-consuming, often involving queries to counsellors or relatives." (D-521, §3 - 4)	„Ich betreue ein Pflegeheim und suche dieses regelmäßig auf. Die Arbeit mit Hochbetagten ist zeitintensiver, oft verbunden mit Rückfragen an Betreuer oder Angehöhrige.“ (D-521, §3 - 4)

**Theme**	**Subthemes/ codes**	**No of cases coded**	**Group A (physicians)**		**No of cases coded**	**Group B (dentists)**	
Good healthcare in old age	Individual, person-centred view and care	15	"I believe that too much "medicine" is used in this age group. Quality of life and life expectancy in this group of people are given too little consideration. Especially in the area of specialist medicine, the colleagues are too focused on their area of expertise. The overall situation thus receives far too little attention." (S-516, §13)	„Ich glaube das insgesamt in dieser Altersgruppe ein Zuviel an "Medizin" zur Anwendung kommt. Lebensqualität und Lebenserwartung in dieser Personengruppe erfährt zu wenig Berücksichtigung. Gerade im fachärztlichen Bereich sind die Kollegen zu sehr auf Ihren Fachbereich fokussiert. Die Gesamtsituation findet so deutlich zu wenig Beachtung.“ (S-516, §13)	10	"Individual medical care and support! Do not generalise (guidelines and recommendations to which one must increasingly adhere for legal reasons make this more and more difficult)." (D-189, §12)	„Individuelle medizinische Versorgung und Betreuung! Nicht über einen Kamm scheren (Leitlinien und Empfehlungen an die man sich zunehmend aus rechtlichen Gründen halten muss machen dies immer schwieriger).“ (D-189, §12)
	Status and behaviour of patients	3	"Should be compliant" (GP-202, §10)	„Sollte compliant sein“ (GP-202, §10)	2	"One's own fitness, good guidance at all levels". (D-396, §4)	„Die eigene Fitness, gute Betreuung auf allen Ebenen.“ (D-396, §4)
	Patient-relevant outcomes	12	"Preserving self-reliance as long as possible!" (GP-201, §4)	„Erhalt der Eigenständigkeit so lange es geht!“ (GP-201, §4)	12	"For the dentistry field: restore the chewing function as easily as possible". (D-384, §4)	„Für den zahnärztlichen Bereich: die Kaufunktion so einfach wie möglich wiederherstellen.“ (D-384, §4)
	Empathy and appreciation	4	"Do not label as "old"" (GP-518, §4).	„Nicht als "alt" abstempeln“ (GP-518, §4)	4	"People-oriented, caring, adequate" (D-234, §4)	„Menschorientiert, zuwendend, adäquat“ (D-234, §4)
	Communication	6	"Clear language, explanations, comprehensible therapies, so that the patient is enabled to understand his or her illness". (GP-198, §5)	„Verständliche Sprache, Erklärungen, nachvollziehbare Therapien, so das der Patienten in die Lage versetzt wird, seine Erkrankung zu verstehen.“ (GP-198, §5)	2	"Patience, explain everything" (D-434, §4)	„Geduld, alles erklären“ (D-434, §4)

Theme	Subthemes/ codes	No of cases coded	Group A (physicians)		No of cases coded	Group B (dentists)	
	Time	11	"Sufficient time slots for "caring", questioning, examining and treating." (S-223, §4)	„Ausreichende Zeitfenster zum "Kümmern", Befragen, Untersuchen und Behandeln." (S-223, §4)	7	"Time, listening, accompanying, supporting" (D-395, §6)	„Zeit, zuhören, begleiten, unterstützen" (D-395, §6)
	Proactive care	9	"Clear and jointly prepared information on control examinations, appointments and contents of the controls" (GP-158, §6)	„Klare und gemeinsam erstellte Angaben zu Kontrolluntersuchungen, Terminen und Inhalte der Kontrollen" (GP-158, §6)	18	"The foresighted planning in health care. In other words, not a complicated denture, but one that can also be well cared for later, if necessary also by people who take over the oral care of this person. A dental prosthesis that is stable and can be extended". (D-371, §5)	„Die vorausschauende Planung in der Gesundheitsversorgung. Also kein komplizierter Zahnersatz, sondern einer, der sich auch später noch gut pflegen lässt ggf. auch von Personen, die die Mundpflege bei diesem Menschen übernehmen. Ein Zahnersatz der stabil und ausbaufähig ist." (D-371, §5)
	Access and infrastructure	13	" Low threshold, specialist care programme" (S-470, §4)	„Niedrigschwelliges, fachärztlches Versorgungsangebot" (S-470, §4)	10	"The possibility of being seen by a doctor when one may no longer be able to go to a doctor's office oneself". (D-398, §4)	„Die Möglichkeit von einem Arzt aufgesucht zu werden, wenn man vielleicht nicht mehr in der Lage ist, selbst eine Praxis aufzusuchen." (D-398, §4)
	Sufficient and well-trained staff	3	"Sufficient staff to provide care that is humane and dignified" (GP-219, §4).	„Ausreichend Personal zur Gestaltung einer menschen würdigen Pflege" (GP-219, §4)	5	"Educating staff and relatives and including oral hygiene in the standard of care." (D-374, §4)	„Aufklärung des Personals und der Angehörigen und Aufnahme der Mundhyigene in den Pflegestandard." (D-374, §4)
	Cooperation with further actors	5	"All comorbidities should be taken into account. Therefore, close cooperation of all therapists is desirable". (S-516, §4)	„Alle Komorbiditäten sollen Berücksichtigung finden. Daher ist eine enge Kooperation aller Therapeuten	5	"Continuing preventive care also in the nursing home" (D-521, §9)	„Weiterführung der Vorsorge auch im Pflegeheim" (D-521, §9)

Theme	Subthemes/ codes	No of cases coded	Group A (physicians)		No of cases coded	Group B (dentists)	
				wünschenswert.“ (S-516, §4)			
	Patients‘ environments	5	"Social connection, especially support in everyday life" (GP-244, §7).	„Soziale Anbindung, vor allem Unterstützung im Alltag“ (GP-244, §7)	2	"However, many of this age group need support for this, which is often not given". (D-427, §4)	„Viele dieser Altersgruppe brauchen aber dafür Unterstützung, die oft nicht gegeben ist.“ (D-427, §4)
Challenges in caring for people aged 80 and over	Complexity due to multimorbidty and polypharmacy	13	"Polymedication, many clinical indications from different specialities" (S-221, §5).	„Polymedikation, viele Krankheitsbilder aus unterschiedlichen Gebieten“ (S-221, §5)	7	"Daily condition, positioning, variety of medicines that interfere with blood clotting, exclude many analgesics" (D-362, §5).	„Tagesform, Lagerung, Vielzahl der Medikamente, die Blutgerinnung behindern, viele Analgetika ausschließen“ (D-362, §5)
	Influence of physical and cognitive limitations	14	"Communication restrictions resulting from limitation of sensory functions." (GP-525, §7)	„Kommunikationsbeschrän kungen, die sich durch Einschränkung der Sinnesfunktionen ergeben.“ (GP-525, §7)	15	„One can regularly not comply with ergonomics (patients can’t be tilted in the dental chair).” (D-361, §6)	„Ergonomie kann man in der Regel nicht einhalten (Patienten können nicht so im Behandlungsstuhl gekippt werden).“ (D-361, §6)
	Lack of compliance or rejection of treatment	8	„Compliance malfunction / dementia“ (GP-253, §8)	„Compliancestörung / Demenz“ (GP-253, §8)	4	"Patients' unwillingness to accept that they are overburdened with oral hygiene, especially with high-end dentures. They do not like to be helped." (D-439, §7-8)	„Die Uneinsichtigkeit der Patienten, dass sie mit der Mundhygiene überfordert sind, besonders bei hochwertigem Zahnersatz. Sie lassen sich nicht gerne helfen.“ (D-439, §7-8)
	Handling of time resources	10	"To recognise in the briefness of time what is essential for one's own specialty and not to forget human contact." (S-499, §5)	„In der Kürze der Zeit das wesentliche für das eigene Fachgebiet zu erkennen und den menschlichen Kontakt nicht zu vergessen.“ (S-499, §5)	7	"One needs more time due to existing impairments (hearing, vision compliance...)." (D-361, §5)	„Man braucht mehr Zeit durch bestehende Beeinträchtigungen (hören, sehen Compliance...).“ (D-361, §5)
	Relatives and further actors	3	"High sense of entitlement on the part of relatives who either cannot/won't accept	„Hohe Anspruchshaltung der Zugehörigen, die entweder die deutlich	10	"Lack of knowledge about oral hygiene among	„Mangelndes Wissen über Mundhygiene bei Pflegepersonal und

Theme	Subthemes/ codes	No of cases coded	Group A (physicians)		No of cases coded	Group B (dentists)	
			the much more modest goals of their elderly relatives/friends, or often unresolved conflicts that result in overprotectiveness that does not match the capabilities of the persons concerned." (GP-525, §6)	bescheideneren Ziele ihrer alten Verwandten/Freunde nicht akzeptieren können/wollen, oder oft unbearbeitete Konflikte, die in Überfürsorglichkeit münden, die den Möglichkeiten des /der Betroffenen nicht gerecht werden.“ (GP-525, §6)		caregivers and relatives". (D-374, §5)	Angehörigen.“ (D-374, §5)
	Patients’ care goals and (non-medical) further issues	10	"Requested requirement that does not originally belong to medical care" (GP-508, §6)	„Angetragene Anforderung, welche nicht originär zur medizinischen Versorgung gehören“ (GP-508, §6)	3	"Dentistry suitable for the elderly" (D-396, §5)	„Altengerechte Zahnmedizin“ (D-396, §5)
	Structural and organizational challenges	9	"See the first two points. It takes most of the time to get transports authorised to the doctor's office, to contact nursing services and to improve all this home care, I think the medical is the least of the problems." (GP-506, §5)	„Siehe die ersten beiden Punkte. Die meiste Zeit bindet es Transporte in die Praxis genehmigen zu lassen, Pflegedienste zu kontaktieren und diese ganze häusliche Versorgung zu verbessern, das Medizinische ist meines Erachtens das geringste Problem.“ (GP-506, §5)	9	"Collecting all important information (medical data, contact details of relatives/carers/etc.)" (D-371, §7).	„Sammeln aller wichtigen Informationen (medizinische Daten, Kontaktdaten Angehörige/ Betreuer/etc.)“ (D-371, §7)
	No challenges	1	"None. I now prefer to work with the older patients than with the younger ones." (S-222, §8)	„Keine. Ich arbeite mittlerweile lieber mit den Älteren Patienten als mit den Jüngeren.“ (S-222, §8)	1	„None“ (D-432, §5)	„Keine“ (D-432, §5)
Reasons for not providing the desired care	Patients‘ (health) status and abilities	7	"To interactions (additive effects) of the existing medication with my desired prescription" (S-226, §12).	„An Interaktionen (additive Effekte) der vorhandenen Medikation mit meiner gewünschten Verordnung“ (S-226, §12)	22	"Signs of ageing such as bone loss make therapies partly impossible (prosthesis without bone simply does	„Alterserscheinungen wie Knochenabbau machen Therapien zum Teil unmöglich (Prothese ohne Knochen hält einfach

**Theme**	**Subthemes/ codes**	**No of cases coded**	**Group A (physicians)**		**No of cases coded**	**Group B (dentists)**	
						not adhere, no matter what one tries)". (D-398, §7)	nicht, egal was man auch versucht).“ (D-398, §7)
	Patients‘ environments and further actors	11	"Lack of support from the environment" (GP-524; §13)	„Fehlende Unterstützung durch das Umfeld“ (GP-524; §13)	7	"The relatives' unwillingness to take care and invest money in health". (D-454, §9)	„An der fehlenden Bereitschaft der Angehörigen sich zu kümmern und Geld in die Gesundheit zu investieren“ (D-454, §9)
	Lack of compliance or discernment	14	"Lack of insight, lack of understanding, lack of compliance" (S-232, §7).	„Fehlende Einsicht, fehlendes Verständnis, fehlende Compliance“ (S-232, §7)	9	"The patient always wants only the most necessary. Then the problems arise when the general condition no longer permits sensible therapy". (D-362, §7)	„Der Patient will immer nur das Nötigste. Dann kommen die Probleme, wenn der Allgemeinzustand sinnvolle Therapie nicht mehr zulässt.“ (D-362, §7)
	Financial Reasons	2	"Rejection of prescriptions by the health insurance fund" (GP-518, §7).	„Ablehnung von Verordnungen durch die Krankenkasse“ (GP-518, §7)	16	"Too high a cost of dental prosthesis" (D-449, §7).	„Zu hoher Kostenaufwand bei Zahnersatz“ (D-449, §7)
	Expenditure/lack of time	11	"Too large a time commitment measured against the remuneration" (S-231, §7)	„Zu großer Zeitaufwand gemessen an der Vergütung“ (S-231, §7)	3	"Not enough time" (D-374, §8)	„Nicht ausreichend Zeit“ (D-374, §8)
	Lack of information exchange and cooperation with other actors and disciplines	4	"Lack of information and cooperation with other therapists" (S-516, §7).	„An fehlenden Informationen und mangelnder Kooperation mit anderen Therapeuten“ (S-516, §7)	5	"In the nursing home, lack of cooperation from professional carers. Money does matter." (D-521, §13)	„Im Pflegeheim mangelnde Kooperation von Berufsbetreuern. Geld spielt eine Rolle.“ (D-521, §13)
	Lack of (qualified) staff	5	"Lack of resources in the system (e.g. physiotherapy, occupational therapy, psychotherapy)" (GP-202, §16).	„Fehlende Ressourcen im System (z.B. Physio- Ergo- Psychotherapie)“ (GP-202, §16)	1	"Staff shortages" (D-395, §9)	„Personelle Engpässe“ (D-395, §9)

**Theme**	**Subthemes/ codes**	**No of cases coded**	**Group A (physicians)**		**No of cases coded**	**Group B (dentists)**	
	Bureaucracy	2	"See above. Medically, my patients are well cared for, but often there is no visiting service against loneliness, meals on wheels, help in case of deterioration of the physical situation, who applies for a care level, etc.? That is totally difficult for most of them." (GP-506, §7)	„Siehe vorne. Medizinisch sind meine Patienten gut versorgt, aber oft fehlt der Besuchsdienst gegen Einsamkeit, das Essen auf Rädern, Hilfe bei Verschlechterung der körperlichen Situation, wer beantragt Pflegestufe etc. Das ist total schwierig für die Meisten." (GP-506, §7)	1	"Costs and government craziness" (D-305, §6)	„Kosten und Behördenwahnsinn" (D-305, §6)
	Design of healthcare infrastructure	8	"Underprovision of age appropriate transportation and delivery systems." (S-175, §8)	„Unterversorgung an altersgerechten Transport- und Versorgungssystemen." (S-175, §8)	7	"Restricted treatment methods in outpatient practice as a dentist in outreach care. Partly in cost sharing. It is directly said that if own costs arise, we do not want treatment without being shown the options and benefits for the patient." (D-427, §7)	„Eingeschränkte Behandlungsmethoden in ambulanter Tätigkeit als Zahnarzt bei der aufsuchenden Betreuung. Teils bei der Kostenbeteiligung. Es wird direkt gesagt, wenn eigene Kosten entstehen, wünschen wir keine Behandlung, ohne sich die Möglichkeiten und den Nutzen für den Patienten aufzeigen zu lassen." (D-427, §7)
Facilitators of providing good care	None	0	-	-	1	„Nothing" (D-305, §7)	„Nichts" (D-305, §7)
	Care providers' experiences	5	"Personal experience" (GP-512, §14)	„Persönliche Erfahrung" (GP-512, §14)	3	"My long experience helps me". (D-420, §23)	„Meine lange Erfahrung hilft mir." (D-420, §23)
	Care providers' qualifications and training	7	"Well-trained employees" (GP-514, §8)	„Gut ausgebildete Mitarbeiter" (GP-514, §8)	6	"Regular training in this field" (D-371, §11)	„Regelmäßige Fortbildungen in diesem Bereich" (D-371, §11)

Theme	Subthemes/ codes	No of cases coded	Group A (physicians)		No of cases coded	Group B (dentists)	
	Care providers‘ attitudes and motivation	19	"The thought of being treated as I see fit for once". (GP-158, §11)	„Der Gedanke selber mal so behandelt zu werden, wie ich es als angemessen sehe.“ (GP-158, §11)	13	"My own motivated staff who see the care of this vulnerable patient group as an enrichment of their work." (D-427, §8)	„Meine eigenen motivierten Mitarbeiter, die die Versorgung dieser vulnerablen Patientengruppe als Bereicherung ihrer Arbeit ansehen.“ (D-427, §8)
	Encounters with patients	13	"The patients all have something to tell. Almost nobody listens to them anymore. I was allowed to meet very wonderful people and learn from them." (GP-198, §10)	„Die Patienten haben alle etwas zu erzählen. Fast niemand hört ihnen noch zu. Ich durfte ganz wunderbare Menschen kennenlernen und von ihnen lernen.“ (GP-198, §10)	4	"The gratitude of the patients" (D-454, §10)	„Die Dankbarkeit der Patienten“ (D-454, §10)
	Patients‘ (health) status	2	"Mobility of the patient" (S-499, §8)	„Mobilität des Patienten“ (S-499, §8)	2	"Patient mobility, Nursing assistant, Patient manual dexterity" (D-376, §7).	„Mobilität des Patienten, Pflegekraft, manuelle Geschicklichkeit der Patienten“ (D-376, §7)
	Interactions with further stakeholders	12	"Intensive dialogue with guardians" (GP-202, §18)	„Intensiver Austausch mit Betreuungspersonen“ (GP-202, §18)	8	"Family support and presence during decisions and treatment" (D-395, §10)	„Familiäre Unterstützung und Anwesenheit bei Entscheidungen und Behandlungen“ (D-395, §10)
	Time	4	„Enough time“ (S-499, §8)	„Ausreichend Zeit“ (S-499, §8)	1	"And time to be responsive to patients" (D-433, §8).	„Und Zeit, um auf die Patienten einzugehen“ (D-433, §8)
	Interdisciplinary cooperation	7	"Good contact with professional colleagues, therapists, assistant professions" (GP-202, §17)	„Guter Kontakt zu Fachkollegen, Therapeuten, Assistenzberufen“ (GP-202, §17)	1	"Very close contacts in a rural doctor's office. People know each other." (D-521, §16)	„Sehr enge Kontakte in einer Landpraxis. Man kennt sich.“ (D-521, §16)
	Local structures and offers	4	"If the patients allow it, I try to integrate them into a network: Home visit by me or my non-medical practice	„Sofern es die Patienten erlauben, versuche ich sie in ein Netzwerk einzubinden: Hausbesuch	1	"The question should be who! Politically, something must change, more doctors should be prepared to care	„Die Frage sollt sein wer! Politisch muss sich etwas tun, mehr Ärzte sollten dazu bereit sein Patienten

**Theme**	**Subthemes/ codes**	**No of cases coded**	**Group A (physicians)**		**No of cases coded**	**Group B (dentists)**	
			assistants, day care, offers by Caritas, walks with volunteers, etc." (GP-198, §10)	durch mich oder meine EVAS [nichtärztliche Praxisassistenz], Tagespflege, Angebote durch die Caritas, Spaziergänge mit Ehrenamtlichen etc..“ (GP-198, §10)		for patients. Reducing bureaucracy is important. So is publicising hygiene measures and the possibility of home visits." (D-374, §9)	zu versorgen. Entbürokratisierung ist wichtig. Genau so das Publizieren von Hygienemaßnahmen und der Möglichkeit der Hausbesuche.“ (D-374, §9)
	Supporting tools and programmes	4	„Medicationplan“(S-499, §8)	„Medikationsplan“ (S-499, §8)	0	-	-
Ensuring a feeling of safety	Not possible	1	„Not at all“ (GP-508, §11)	„Gar nicht“ (GP-508, §11)	0	-	-
	Familiar environment	2	"Familiar Environment" (GP-310, §9)	„Vertrautes Umfeld“ (GP-310, §9)	2	“About the trusted person/doctor/dentist Dentist" (D-415, §9)	„An der vertrauten Person/Arzt/Zahnarzt Zahnärztlicher“ (D-415, §9)
	Timely contact options	4	"Easier access to healthcare" (S-231, §9)	„Leichterer Zugang zur Versorgung“ (S-231, §9)	1	"Timely care“ (D-451, §22)	„Zeitnahe Betreuung) (D-451, §22)
	Proactive care	2	"Medical practitioner / general practitioner as easily accessible contact person, who leads and schedules care" (GP-158, §12 - 13)	„Mediziner / Hausarzt als gut erreichbarer Ansprechpartner, der die Versorgung führt und terminiert“ (GP-158, §12 - 13)	3	"Regular checks" (D-439, §15)	„Regelmäßige Kontrollen“ (D-439, §15)
	Support	5	"Supporting aid" (GP-524, §18)	„Unterstützende Hilfsmittel“ (GP-524, §18)	6	"Assistance by the team and the practitioner; disability-friendly practice) (D-384, §9).	„Hilfe durch das Team und den Behandler; behindertengerechte Praxis) (D-384, §9)
	Good communication, counselling and conversations	17	"Through conversation: understanding what the patient fears and wants." (GP-202, §20)	„Durch Gesprächsführung: Verstehen, was der Patient befürchtet und wünscht.“ (GP-202, §20)	7	“Take patients seriously, perceive them and see them. Time, attention, listening, explaining, showing.” (D-189, §24)	„Patienten ernst nehmen, wahrnehmen und ihn sehen. Zeit, Zuwendung, Zuhören, Erklären, Zeigen.“ (D-189, §24)

**Theme**	**Subthemes/ codes**	**No of cases coded**	**Group A (physicians)**		**No of cases coded**	**Group B (dentists)**	
	Attention and appreciative behaviour, relationship-building	12	"Allowance, sympathy for their worries and fears" (GP-198, §11)	„Zuwendung, Anteilnahme an ihren Sorgen und Befürchtungen" (GP-198, §11)	19	"By treating the patient as equals" (D-435, §9)	„Durch einen Umgang mit den Patienten auf Augenhöhe" (D-435, §9)
	Sufficient time	1	"Sufficient time for discussions and implementation of the measures in order to be able to take age-related limitations into account". (S-169, §9)	„Ausreichend Zeit für Gespräche und Durchführung der Maßnahmen um die altersbedingten Einschränkungen berücksichtigen zu können" (S-169, §9)	5	"Calm treatment without time stress" (D-433, §9)	„Ruhige Behandlung ohne zeitlichen Stress" (D-433, §9)
	Competence and education	2	"Good advanced vocational training " (S-216, §13)	„Gute Fortbildung" (S-216, §13)	3	"Regular further training also specifically in relation to this patient clientele" (D-371, §12).	„Regelmäßige Fortbildungen auch speziell in Bezug auf dieses Patientenklientel" (D-371, §12)
Ensuring a feeling like that of a meaningful human being	Not possible	1	"I can't guarantee" (S-232, §10)	„Kann ich nicht gewährleisten" (S-232, §10)	0	-	-
	Good communication, counselling and conversations	4	"Showing empathy through facial expression and gestures, listening first – then typing what has been heard into the PC." (S-226, §15)	„Mimisch und gestisch Empathie zeigen, erst Zuhören - dann das Gehörte in den PC tippen." (S-226, §15)	7	" One also talks to each other about more than just illness". (D-422, §10)	„Man redet miteinander auch mehr als nur über Krankheit" (D-422, §10)
	Attention and appreciative behaviour, relationship-building	25	"Appreciation of Age" (GP-244, §24)	„Würdigung des Alters" (GP-244, §24)	19	"Sincere condolences and respect" (D-451, §23)	„Aufrichtige Anteilnahme und Respekt" (D-451, §23)
	Sufficient time	9	"Enough time for Conversations"	„Ausreichend Zeit für Gespräche"	6	**„**To take time" (D-521, §20)	„Sich Zeit nehmen" (D-521, §20)

Theme	Subthemes/ codes	No of cases coded	Group A (physicians)		No of cases coded	Group B (dentists)	
			(GP-520, §22)	(GP-520, §22)			
	Engagement with individual needs and wishes, holistic view	8	"Individualise therapy, diagnostics and care". (GP-158, §14)	„Therapie, Diagnostik und Fürsorge individuell gestalten" (GP-158, §14)	9	"The patient is respected as a personality and not seen as a case" (D-362, §10).	„Der Patient wird als Persönlichkeit respektiert und nicht als Fall gesehen" (D-362, §10)
	Social integration	4	"Social Connection" (GP-244, §24)	„Soziale Anbindung" (GP-244, §24)	0	-	-
Ensuring the maintenance of control and independence	Not (always) possible	3	"Unfortunately, I cannot always guarantee this, as I sometimes have the role of the one who must also take measures to protect the patient, e.g. suggesting and advocating care- Sometimes frank words are indispensable in this." (S-196, §16)	„Das kann ich leider nicht immer gewährleisten, da ich manchmal die Rolle derjenigen habe, die zum Schutze des Patienten auch Maßnahmen ergreifen muss, z.B. Anregung und Befürwortung einer Betreuung- Manchmal sind offene Worte dabei unentbehrlich." (S-196, §16)	1	"Certainly, a dentist cannot establish autonomy. We can ensure a guarantee of care and we are only supported by our professional representatives, but not by politics." (D-374, §12)	„Sicherlich kann ein Zahnarzt keine Autonomie herstellen. Wir können eine Gewährleistung der Versorgung sicherstellen und werden dabei nur durch unsere Standesvertreter unterstützt, jedoch nicht durch die Politik." (D-374, §12)
	Engagement with individual needs and wishes, holistic view	1	"By respecting their life (end) goals." (GP-525, §14)	„Durch Respektierung ihrer Lebens(endes)ziele." (GP-525, §14)	2	"The patient's wishes are respected" (D-362, §11)	„Wünsche des Patienten werden respektiert" (D-362, §11)
	Good communication and counselling	5	"Good advice for your own decision" (GP-514, §11)	„Gute Beratung zur eigenen Entscheidung" (GP-514, §11)	3	"Assistance with treatment decisions" (D-423, §11)	„Hilfestellung bei Therapieentscheidungen" (D-423, §11)
	Appreciative behaviour	1	"By attempting empathy" (S-223, §14)	„Durch Versuch der Empathie" (S-223, §14)	3	"Respectful treatment" (D-447, §16)	„Wertschätzender Umgang" (D-447, §16)
	Enabling decision options	6	"Involvement in decision-making processes, taking	„Einbindung in Entscheidungsprozesse	6	"In treatment planning, point out treatment alternatives	„Bei der Behandlungsplanung

Theme	Subthemes/ codes	No of cases coded	Group A (physicians)		No of cases coded	Group B (dentists)	
			into account the above factors". (S-516, §11)	unter Berücksichtigung o.g. Faktoren“ (S-516, §11)		and let the patient make the decision himself" (D-416, §20).	Behandlungsalternativen aufzeigen und Entscheidung selber treffen lassen“ (D-416, §20)
	Encouragements and support without paternalism	5	"Avoiding paternalism and restriction through medical measures". (S-221, §11)	„Vermeiden von Bevormundung und Einschränkung durch medizinische Maßnahmen“ (S-221, §11)	5	"Do not patronise, but offer assistance". (D-371, §14)	„Nicht bevormunden, aber Hilfestellung anbieten“ (D-371, §14)
	Maintaining and expanding functionalities, prevention	11	"Emphasising and reinforcing the skills they are still able to do. Motivating and supporting patients." (GP-244, §25)	„Betonung und Verstärkung der Fähigkeiten, die sie noch können. Motivierung und Unterstützung der Patienten.“ (GP-244, §25)	1	"Restoration of chewing function and aesthetics where possible" (D-384, §11).	„Wiederherstellung der Kaufunktion und Ästhetik, wo es geht“ (D-384, §11)
	Structures of support and care	15	"Support for independent living at home (home emergency call, mobility preserving Support)" (GP-520, §23 - 24)	„Unterstützung beim selbständigen Leben zu Hause (Hausnotruf, mobilitätserhaltende Unterstützung)“ (GP-520, §23 - 24)	5	"Family Involvement" (D-384, §11)	„Einbindung der Familie“ (D-384, §11)
Measures to improve healthcare in old age	Prevention orientation	3	"Maintain health from a young age: Exercise, do not overeat, do not smoke, do not drink too much alcohol." H-353, §12)	„Von jungen Jahren an Gesundheit erhalten: Bewegung, keine Überernährung, nicht Rauchen, nicht zu viel Alkohol.“ H-353, §12)	4	"More paid prophylaxis" (D-376, §11)	„Mehr bezahlte Prophylaxe“ (D-376, §11)
	Patient orientation	6	"I would like to see patient-oriented medicine. Unfortunately, it's all about maximising profits. Politicians and business associations are trying hard in this regard. I have been	„Ich wünsche mir eine patientenorientierte Medizin. Leider geht es nur um Gewinnmaximierung. Die Politik und Wirtschaftsverbände geben	2	"Responding to the needs of the elderly." (D-382, §12)	„Auf Bedürfnisse der älteren Menschen eingehen.“ (D-382, §12)

Theme	Subthemes/ codes	No of cases coded	Group A (physicians)		No of cases coded	Group B (dentists)	
			active in medical policy for years. The situation of medicine is constantly changing, just not for the better."<br>(GP-198, §16 - 17)	sich diesbezüglich alle Mühe. Seit Jahren bin ich in der Medizinpolitik tätig. Die Lage der Medizin verändert sich stetig, nur nicht zum Besseren.“<br>(GP-198, §16 - 17)			
	Empathy	2	"In my position as a doctor, I feel like a fossil. None of the things I started out to do 33 years ago still hold true. The commercialisation of our profession is a disgrace. I don't see a patient, no matter what age, as an economic factor. I treat people, no age, no skin colour, no nationality, no wallet. This is laughed at, but a medicine without heart or empathy is degraded to pure technology-loving medicine. I look forward to my future as a patient with great anxiety."<br>(GP-198, §14)	„In meiner Rolle als Ärztin fühle ich mich als Fossil. Nichts von dem, weswegen ich vor 33 Jahren angetreten bin, hat noch Bestand. Die Kommerzialisierung unseres Berufsstandes ist eine Schande. Ich sehe in einem Patienten, egal welchen Alters, keinen Wirtschaftsfaktor. Ich behandle Menschen, kein Alter, keine Hautfarbe, keine Nationalität, kein Portemonnaie. Dies wird belächelt, aber eine Medizin ohne Herz oder Empathie wird zur reinen technikverliebten Medizin herabgewürdigt. Meiner Zukunft als Patientin sehe ich mit großer Sorge entgegen.“<br>(GP-198, §14)	2	"But equally important is more empathy. You have to like your fellow human beings so that you can stand up for them, I think.<br>(D-427, §12)	„Aber ebenso wichtig ist mehr Empathie. Man muss seine Mitmenschen mögen, damit man sich für Sie einsetzt.finde ich.“<br>(D-427, §12)
	Reimbursement	16	"Better rewards for speaking medicine"<br><br>(GP-202, §23)	„Bessere Honorierung der sprechenden Medizin“<br><br>(GP-202, §23)	12	"Promote mobile dentistry, which is very, very costly, time-consuming and	„Förderung der mobilen Zahnmedizin, die in der aufsuchenden Betreuung in privaten Haushalten

Theme	Subthemes/ codes	No of cases coded	Group A (physicians)		No of cases coded	Group B (dentists)	
						underpaid in outreach care in private households." (D-371, §15)	sehr, sehr aufwendig, zeitintensiv und unterbezahlt ist.“ (D-371, §15)
	More time	10	"More time for these patients, respectively better fees so that more time can be taken". (S-232, §12)	„Mehr Zeit für diese Patienten, respektiv besseres Honorar, damit mehr Zeit genommen werden kann.“ (S-232, §12)	4	"Invest more time and work and thus in fair remuneration" (D-436, §12)	„Mehr Zeit und Arbeit und damit in gerechte Entlohnung investieren“ (D-436, §12)
	Access	2	"Easier access to outpatient services" (GP-192, §23)	„Leichterer Zugang zu ambulanten Hilfen“ (GP-192, §23)	4	"Improving the care of immobile patients (outreach care)". (D-399, §13)	„Verbesserung der Versorgung von immobilen Patienten (aufsuchende Versorgung)“ (D-399, §13)
	Support structures	15	"Patient guides who take over organisational tasks and are a good and informed contact person for the older patient". (GP-158, §16)	„Patienten-Lotsen die organisatorische Aufgaben übernehmen und dem älteren Patienten ein guter und informierter Ansprechpartner sind“ (GP-158, §16)	10	"Better care in senior facilities such as dental treatment units and appropriate premises." (D-423, §12)	„Bessere Betreuung in den Senioreneinrichtungen wie zahnärztliche Behandlungseinheiten und entsprechende Räumlichkeiten.“ (D-423, §12)
	Information exchange and interdisciplinary cooperation	5	"Provide a health record containing all the patient's information such as pre-existing conditions, pre-operations, medications and findings from examinations" (S-169, §4).	„Bereitstellen einer Gesundheitsakte mit allen Informationen des Patienten wie Vorerkrankungen, Vor-OPs, Medikamente und Befunde von Untersuchungen“ (S-169, §4)	4	"Cooperation between general practitioners, mobile care services and dentists". (D-454, §14)	„Zusammenarbeit zwischen Hausärzten, mobilen Pflegediensten und Zahnärzten“ (D-454, §14)
	Simplification	0	-	-	2	"Reduction of bureaucracy" (D-434, §12)	„Abbau der Bürokratie“ (D-434, §12)
	Qualification and training	3	"Further education in geriatrics" (S-163, §13)	„Fortbildungen Geriatrie“ (S-163, §13)	7	"Timely admission to university education. To	„Rechtzeitige Aufnahme in die universitären

Theme	Subthemes/ codes	No of cases coded	Group A (physicians)		No of cases coded	Group B (dentists)	
						strengthen the realisation of how quickly we ourselves belong to this age group" (D-427, §12)	Ausbildungen. Die Erkenntnis zu stärken, wie schnell wir selbst dieser Altersgruppe angehören“ (D-427, §12)
	More (qualified) staff	5	"More qualified staff" (S-372, §12)	„Mehr qualifiziertes Personal“ (S-372, §12)	3	"Staff increase " (D-395, §14)	„Personelle Aufrüstung“ (D-395, §14)
	Consideration of non-medical dimensions	8	"I think the biggest problem is the lack of and confusing assistance services and that patients are left so alone with this. There are also no contact persons for me to inform that a lonely old person needs a visit or is desperate because he cannot programme TV programmes. Everyone is overwhelmed with applications for assistance.” (GP-506, §14)	„Ich glaube das größte Problem sind die fehlenden und unübersichtlichen Hilfeleistungen und dass Patienten damit so alleine gelassen werden. Es gibt auch für mich keine Ansprechpartner, die ich informieren kann, dass ein einsamer alter Mensch einen Besuch braucht, oder verzweifelt, weil er Fernsehprogramme nicht programmieren kann. Mit Beantragung von Hilfsleistungen sind alle überfordert.“ (GP-506, §14)	1	"More engagement of the relatives" (D-415, §12)	„Mehr Einsatz der Angehörigen“ (D-415, §12)
	Strengthening the relevance of oral health	0	-	-	14	"Educating caregivers about the importance of oral hygiene." (D-399, §13)	„Aufklärung der Pflegenden über Bedeutung der Mundhygiene.“ (D-399, §13)

Theme	Subthemes/ codes	No of cases coded	Group A (physicians)		No of cases coded	Group B (dentists)	
Other	Upcoming developments and challenges	5	"I have no hope for improvement. Too many very old people meet too few carers and the demands of individuals demotivate." (GP-508, §16 - 17)	„Ich habe keine Hoffnung auf Verbesserung. Zu viele Hochbetagte treffen auf zu wenig Kümmerer und die Ansprüche Einzelner demotivieren.“ (GP-508, §16 - 17)	5	"Not only medicine, but society as a whole must adapt to increasingly older people and not send them to the siding. Old people also have their needs. They should be treated respectfully." (D-382, §13 - 15)	„Nicht nur die Medizin, sondern die gesamte Gesellschaft muss sich auf immer älter werdende Menschen einstellen und nicht aufs Abstellgleis schicken. Auch alte Menschen haben ihre Bedürfnisse. Sie sollten respektvoll behandelt werden.“ (D-382, §13 - 15)
