# Supplementary material for: Providing ambulatory healthcare for people aged 80 and over: Views and perspectives of physicians and dentists from a qualitative survey
Source: PLoS One. 2022 Aug 15;17(8):e0272866. doi: 10.1371/journal.pone.0272866 (PMC9377615; doi:10.1371/journal.pone.0272866)
Supplement: S3 Appendix — (PDF) [file pone.0272866.s003.pdf]

S3 Appendix: Full coded material from the open items

	Specialists	General practitioners	Dentists
**Eigenschaften der älteren Patient:innen**			
Rolle von Leben und Erfahrungen	Ältere Patienten sind oft reflektierter, mit mehr Lebenserfahrung, realistischer und viel öfter bereit, etwas für die eigene Genesung oder Lebensqualität zu tun und zu investieren<br><br>S-196: 4 - 4 (0)	Diese Patientengruppe ist sehr pragmatisch, da sie im Leben oftmals viele Entbehrungen und Verluste erlebten.<br><br>GP-198: 3 - 3 (0)	Zugriff auf andere emotionale Vergangenheit, Leben und zahnärztliche Erfahrung<br><br>D-451: 5 - 5 (0)
Positive Eigenschaften	ich werde weniger oft angelogen!<br>Ältere Patienten sind oft reflektierter, mit mehr Lebenserfahrung, realistischer und viel öfter bereit, etwas für die eigene Genesung oder Lebensqualität zu tun und zu investieren<br><br>S-196: 3 - 4 (0)<br><br>oft hohe Arztbindung<br><br>S-221: 3 - 3 (0)<br><br>freundlich<br><br>S-221: 3 - 3 (0)	Diese Patientengruppe ist sehr pragmatisch, da sie im Leben oftmals viele Entbehrungen und Verluste erlebten.<br><br>GP-198: 3 - 3 (0)<br><br>Viele Patienten sind dankbarer<br><br>GP-202: 5 - 5 (0)<br><br>man erhält mehr Dankbarkeit, es macht mehr Spaß<br><br>GP-253: 4 - 4 (0)<br><br>Diese Altersgruppe ist überwiegend gelassener<br><br>GP-271: 3 - 3 (0)<br><br>sehr schönes Arbeiten mit den "Alten"<br><br>GP-518: 3 - 3 (0)<br><br>Dennoch ist für mich geriatrische Medizin ein ganz besonderes Fach, das ich gerne erfülle.<br><br>GP-525: 4 - 4 (0)	-oft freundliche, sympatische und vor allem respektvolle Patienten<br><br>D-189: 9 - 9 (0)<br><br>-nehmen Termine wahr und sind pünktlich<br><br>D-189: 10 - 10 (0)<br><br>-schätzen die Hilfe wert<br><br>D-189: 11 - 11 (0)<br><br>in der Regel sehr dankbare Patienten<br><br>D-361: 3 - 3 (0)<br><br>Die ü80 jährigen sind eine sehr ersterbenswerte Patientengruppe (Solvent, Pünktlich Höflich)<br><br>D-367: 4 - 4 (0)<br><br>teilweise sehr höflich, aber auch direkt,<br>D-396: 3 - 3 (0)<br><br>aufgeklärt gesundheitsbewußt selbstständig<br><br>D-415: 3 - 3 (0)<br><br>Trotzdem ist diese Patientengruppe gut zu betreuen, gerade, wenn man die Menschen seit vielen Jahren kennt. Sie sind sehr dankbar.<br><br>D-521: 5 - 5 (0)

	Specialists	General practitioners	Dentists
Herausfordernde Eigenschaften	oft schlechter vorbereitet zu den Terminen<br><br>S-163: 3 - 3 (0)<br><br>keiner hat ne ahnung aber jeder hat eine meinung......<br>brille nicht mit<br>hörgerät nicht mit<br>medikamentenliste nicht mit............<br>S-217: 3 - 6 (0)<br><br>in Teilen verlangsamt, behäbiger<br>S-221: 3 - 3 (0)<br><br>Einige scheinen mir ängstlich und sehr unsicher zu sein.<br>S-222: 3 - 3 (0)<br><br>Eininige wenige haben viel Gesprächsbedarf und freuen sich, wennn ich einfach zuhöre.<br><br>S-222: 3 - 3 (0)<br><br>Manche sind mental fit, selbstbestimmt und fordernd, andere sehr unsicher und verlangsamt.<br><br>S-226: 4 - 4 (0)<br><br>Redebedarf - mehr Zeit<br>S-322: 3 - 3 (0)<br><br>generell mehr Gesprächsbedarf,<br>S-325: 3 - 3 (0)<br><br>Andererseits haben sie oft gute Fluchtreflexe und zucken öfters zurück z.B. bei Gehörgangsreinigungen, sie scheinen auch empfindlicher zu sein.<br>S-372: 3 - 3 (0)<br><br>gebrechlicher<br>S-470: 3 - 3 (0)	viele soziale und allltagsassoziierte Probleme, die die medizinische Versorgung errschweren.<br><br>GP-192: 3 - 3 (0)<br><br>schlechte soziale Anbindung/Versorgung mit vielen organisatorischen Problemen, worunter auch die Patienten leiden.<br><br>GP-244: 5 - 5 (0)<br><br>ältere Patienten haben einen höheren Redebedarf oft mit weniger Anspruch auf medizinische Versorgung und Verbesserungen, sondern mehr Psychohygiene und Erhalt des status idem, Bestätigung, dass alles gut so ist, sich auch nicht mehr unbedingt verbessern läßt ("das ist halt das Alter")<br><br>GP-245: 3 - 3 (0)<br><br>wegen Einsamkeit oft besondere Kommunikationswünsche<br><br>GP-520: 6 - 6 (0)<br><br>sind oft einsam, brauchen tips und Hilfe für Alltagsfragen (wie und wo kann ich Menschen kennenlernen oder Hilfe bekommen? wer hlft mir meinen Fernseher zu programmieren? Finden sich im Internet nicht zurecht, wissen nicht wie Pflegstufen zu beantragen sind, finden Telefonnummern für die kranaknekasse nicht...)<br><br>GP-506: 3 - 3 (0)<br><br>Ängstlichkeit<br><br>GP-524: 5 - 5 (0)	-Fehlende/Schwerfällige Anpassungsfähigkeit an Neuerungen<br><br>D-189: 3 - 3 (0)<br><br>Terminabsagen<br><br>D-411: 6 - 6 (0)<br><br>Redebedarf<br><br>D-434: 3 - 3 (0)<br><br>Sensibler<br><br>D-454: 3 - 3 (0)
**Gesundheitlicher Zustand und Behandlungsbedarf**			

	Specialists	General practitioners	Dentists
Komplexität durch Multimorbidität und Polypharmazie	Multi morbide<br>Poly Pharmazie<br><br>S-216: 3 - 4 (0)<br><br>keiner hat ne ahnung aber jeder hat eine meinung......<br>brille nicht mit<br>hörgerät nicht mit<br>medikamentenliste nicht mit............<br><br>S-217: 3 - 6 (0)<br><br>Häufig liegt eine mutlti-drug-situation vor, mit mehr als 10 Wirkstoffen.<br><br>S-226: 9 - 9 (0)<br><br>Polypharmazie<br><br>S-470: 3 - 3 (0)<br><br>Kontrolluntersuchungen schwieriger, da oft begelitperson erfoderlcih/mobilitätseinschränkung, Schwerhörigkeit, Vergeßlichkeit<br><br>S-470: 3 - 3 (0)<br><br>Aber auch besondere Herausforderungen: Polypharmakotherapie, allg. Verlangsamung, Pflege- und Betreuungsbedürtigkeit<br><br>S-499: 3 - 3 (0)	Multimorbidität<br><br>GP-201: 3 - 3 (0)<br><br>Viele Krankheiten mit vielen Medikamenten, was die Patienten überfordert.<br><br>GP-244: 3 - 3 (0)<br><br>Komplexe Fälle und geringe Geschwindigkeit (körperlich aber auch mental) mit größerer Zeitaufwand<br><br>GP-244: 4 - 4 (0)<br><br>häufiger multimorbide<br><br>GP-310: 3 - 3 (0)<br><br>im Verlauf dann oft sehr zeitintensiv durch hohen Betreuungsbedarf, Medikation, Pflegedienste, Physiotherapeuten, Pflegestufen; palliative Begleitung, Kommunikation mit Angehörigen.<br><br>GP-518: 3 - 3 (0)<br><br>andere Verstoffwechselung von Medikamenten<br><br>GP-520: 3 - 3 (0)<br><br>Geriatrische Pat. sind anspruchsvoll, geriatrische Medizin ist MITNICHTEN ein Herunterbrechen der Therapie auf den alten Menschen.<br><br>GP-525: 4 - 4 (0)<br><br>die Gruppe der über 80 jährigen ist aufgrund der Multimorbidität und der reduzierten Aufffassungsgabe sehr beratungs- und zuwendungsintensiv<br><br>GP-158: 3 - 3 (0)	-Viele Grunderkrankungen bei fehlendem Verständnis für medizinischen Zusammenhang mit Zahnmed.<br><br>D-189: 6 - 6 (0)<br><br>viele Medikamenteneinahmen mit entsprechenden Behandlungseinschränkungen<br><br>D-396: 3 - 3 (0)<br><br>Häufig Einschränkungen in der Behandlung durch Vorerkrankungen. Wechselwirkungen mit Medikamenten.<br><br>D-399: 4 - 4 (0)<br><br>Viele Medikamenten<br><br>D-411: 3 - 3 (0)<br><br>multimorbide Patienten<br><br>D-416: 3 - 3 (0)<br><br>multimorbide<br><br>D-422: 3 - 3 (0)<br><br>Multimedikation erfordert besondere Aufmerksamkeit.<br><br>D-423: 3 - 3 (0)
Körperliche und kognitive Einschränkungen	Einschränkungen bei Hör- und Sehvermögen und Mobilität<br><br>S-169: 3 - 3 (0)	manchmal zusätzlich Demenz<br><br>GP-201: 3 - 3 (0)	-Atrophie der Gewebe sodass Nerven oberflächlich liegen und Schmerzen verursachen<br><br>D-189: 4 - 4 (0)

Specialists	General practitioners	Dentists
Die Pat. sind nicht so mobil und benörigen mehr Zeit,<br><br>S-175: 3 - 3 (0)<br><br>ihre Auffassungsgabe ist manchmal schlechter und auch ihr Gehörsinn und ihr Sehvermögen.<br><br>S-175: 3 - 3 (0)<br><br>Die Patientinnen und Patienten (bitte nicht Patient*innen, das ist für beide Geschlechter diskriminierend) sind aufgrund zunehmender geistiger und körperlicher Immobilität langsamer als andere und benötigen daher mehr Zeit und Hilfe.<br><br>S-223: 3 - 3 (0)<br><br>Eine häufig schlechtes Gehör erschwert die Kommuniktion recht deutlich.<br><br>S-226: 5 - 5 (0)<br><br>Demente Patienten sind zumeist in Begleitung ihrer Angehörigen oder Pflegenden.<br><br>S-226: 6 - 6 (0)<br><br>Ein metabolisches Syndrom macht einfach bewegungsarm und unfit.<br><br>S-226: 8 - 8 (0)<br><br>eingeschränkte Mobilität,<br><br>S-232: 3 - 3 (0)<br><br>Einschränkung kognitiver Fähigkeiten<br><br>S-232: 3 - 3 (0)<br><br>meist schwerhörige Patienten , ich benötige mehr Zeit für Erläuterungen ,	Bei Demenzpatienten ist mehr Gedulgd und Einfühlungsvermögen erforderlich<br><br>GP-202: 4 - 4 (0)<br><br>Es kommt eher vor, dass Kommunikationsprobleme aufgrund der Altersschwerhörigkeit vorkommen.<br><br>GP-219: 3 - 3 (0)<br><br>Einschränkungen der Wahrnehmung und Bewegung<br><br>GP-275: 3 - 3 (0)<br><br>Betreuung über Jahre, dann oft plötzliche Veränderungen (Kognition/Mobilität); gute Arzt-Patienten-Bindung, lange dauerndes Vertrauensverhältnis; Begleitung über die Jahre duch verschidene Lebens-/Gesundheitskrisen;<br><br>GP-518: 3 - 3 (0)<br><br>oft eingeschränkte Leistung der Sinnesorgane<br><br>GP-520: 4 - 4 (0)<br><br>hoher Anteil an dementen Patienten<br><br>GP-520: 7 - 7 (0)<br><br>Neben budgetären Besonderheiten (wegen der fehlenden oder eingeschränkten Mobilität muss hausärztliche Medizin oft auch hochpreisige Medikamente verordnen, die sonst durch ein Facharztbudget gedeckt würden) spielt der Faktor Zeit in der Praxis eine große Rolle.<br><br>GP-525: 4 - 4 (0)<br><br>die Gruppe der über 80 jährigen ist aufgrund der Multimorbidität und der reduzierten Aufffassungsgabe sehr beratungs- und zuwendungsintensiv<br><br>GP-158: 3 - 3 (0)<br><br>oft schwerhörig, seh- und gehbehindert	-Mangelhafte Fingerfertigkeit<br><br>D-189: 5 - 5 (0)<br><br>-Schwierige Lagerung d. Patienten durch Steifheit, Schwindel oÄ<br><br>D-189: 7 - 7 (0)<br><br>-Können oft nicht mehr so lange am Stück behandelt werden<br><br>D-189: 8 - 8 (0)<br><br>große Bandbreite , von topfit bis Grad5.<br><br>D-234: 3 - 3 (0)<br><br>wir behandeln sehr viele Menschen mit Pflegegrad, die Versorgungen unterscheiden sich meistens sehr von der Versorgung jüngerer Pat.<br><br>D-361: 3 - 3 (0)<br><br>Die Arbeit mit den über 80 jährigen trägt zur Entschleunigung unserer Praxisabläufe bei. Denn automatisch (müssen) wir uns dem Tempo dieses Patientenkollektivs anpassen. Gibt es Einschränkungen: Mobilität, kognitiv, Hören?, Sehen?<br><br>D-371: 3 - 3 (0)<br><br>Die Einschätzung des Patienten in Bezug auf seine Allgemeingesundheit, allgemeinen Fähigkeiten (z.B. Aktivitäten des täglichen Lebens, manuelle und kognitive Geschicklichkeit, Lebensform) spielt auch in in die zahnmedizinischer Therapieplanung mit hinein.<br><br>D-371: 4 - 4 (0)<br><br>mehr Zeit investieren wegen bestehender Defizite (schlechteres Sehen, hören verminderte Compliance)<br><br>D-383: 4 - 4 (0)<br><br>Eingeschränkte Mobilität<br><br>D-395: 3 - 3 (0)<br><br>Verlangsamte Prozesse im Handeln und Denken

Specialists	General practitioners	Dentists
S-325: 3 - 3 (0)<br><br>Über 80 Jährige sind deutlich langsamer in ihren Bewegungen, verstehen schlechter, können sich öfters die Informationen von mir nicht merken, sind manchmal auch dement.<br><br>S-372: 3 - 3 (0)<br><br>Man muss langsamer und deutlicher mit ihnen sprechen, auch wenn sie nicht hochgradig schwerhörig sind. Tests (z.B. Audiogramme) gehen deutlich erschwerter und langsamer.<br><br>S-372: 3 - 3 (0)<br><br>gebrechlicher<br><br>S-470: 3 - 3 (0)<br><br>Häufung besonderer Krankheitsbilder wie Demenz, Parkinson, Schlaganfälle, NPH, multifaktorielle Gangstörungen.<br><br>S-499: 3 - 3 (0)	GP-512: 3 - 3 (0)<br><br>- eingeschränkte Mobilität<br><br>GP-524: 3 - 3 (0)<br><br>- z.T. langsameres Verständnis komplexerer Zusammenhänbge<br><br>GP-524: 4 - 4 (0)	D-395: 4 - 4 (0)<br><br>Verlust der Sinneswahrnehmungen<br><br>D-395: 5 - 5 (0)<br><br>gebrechlich<br><br>D-396: 3 - 3 (0)<br><br>geminderte Wahrnehmung, physisch und psychisch,<br><br>D-396: 3 - 3 (0)<br><br>Untersuchungen sind aufgrund eingeschränkter Mobilität der Patienten oftmals körperlich belastender.<br><br>D-398: 3 - 3 (0)<br><br>Verschlechterung des Allgemeinzustandes<br><br>D-411: 6 - 6 (0)<br><br>Demenzerkrankung<br><br>D-416: 6 - 6 (0)<br><br>teilweise aufgrund physischer Einschränkung, schwierigere Behandlungssituation<br><br>D-416: 7 - 7 (0)<br><br>Fast jede Behandlung ist schwieriger und anstrengender.<br>Limitierende Faktoren sind u.a. körperliche Einschränkungen und damit verbundene Lagerungsschwierigkeiten.<br><br>D-420: 5 - 6 (0)<br><br>Viele ältere brauchen auch Chirurgie, das ist kein einfacher Job in der Durchführung.<br>Kleinteiliger arbeiten, Prinzip der Zumutbarkeit gewährleisten.<br><br>D-420: 9 - 10 (0)

Specialists	General practitioners	Dentists
		z.T dement;<br><br>D-422: 3 - 3 (0)<br><br>körperliche Einschränkungen; manuelle Defizite<br><br>D-422: 3 - 3 (0)<br><br>Problem dabei sind oft die Demenzkranken; die sehr ängstlich auf die Behandlung reagieren weil Sie nicht einordnen können was passiert und die Kommunikation erschwert ist<br><br>D-427: 3 - 3 (0)<br><br>Schwerhörigkeit und Demenz machen es manchmal schwer, mit dem Pat. zu kommunizieren und das erschwert die Mitarbeit. Aber im Prinzip ist die Behandlung ähnlich zu der Behandlung kleiner Kinder.<br><br>D-433: 3 - 3 (0)<br><br>Bei den Dementzpatienten geht es nicht immer reibungslos und wir benötigen sehr viel Zeit für die Behandlungen, was leider nicht honoriert wird.<br><br>D-439: 3 - 3 (0)<br><br>ältere Menschen können oft nicht mehr so gut hören<br><br>D-447: 3 - 3 (0)<br><br>der Anteil von Menschen mit Demenz ist in dieser Altersgruppe höher.<br><br>D-447: 4 - 4 (0)<br><br>Schwierig in der alltäglichen Behandlungssituation ist nicht der alte, sondern der demente Mensch.<br><br>D-447: 5 - 5 (0)<br><br>Langsame Auffassungsgabe, erschwertes Hören, erschwertes Verständnis, eingeschränkte Bewegungen<br><br>D-449: 3 - 3 (0)<br><br>Beachtung altersspezifische mentale , kognitive Leistungen

	Specialists	General practitioners	Dentists
			D-451: 4 - 4 (0)<br><br>Lagerungsschwierigkeiten<br><br>D-454: 5 - 5 (0)<br><br>können nicht lange sitzen, habeb häufig Rückenproblene<br><br>D-479: 3 - 3 (0)<br><br>können nnicht flach gelagert werden (Herz-Kreislau-Probleme)<br><br>D-479: 4 - 4 (0)
Heterogenes Auftreten & Zustand, ähneln auch jüngeren Patienten	In ihrem Auftreten ähneln sie auch jungen Patienten, je nach grundätzlicher Persönlichkeit.<br>S-226: 3 - 3 (0)<br><br>Manche sind mental fit, selbstbestimmt und fordernd, andere sehr unsicher und verlangsamt.<br>S-226: 4 - 4 (0)<br><br>Ein einheitliches Bild vermag ich da Nicht zu Zeichen weil in deiser Altersgruppe der AZ, die geistige Flexibilität, psychische Verfassung doch sehr heterogen im vergleich zu anderen Alterskohorten ist.<br>S-516: 3 - 3 (0)	keine wesentlicher Mehraufwand zu Jüngeren und meist problemlos<br><br>GP-514: 3 - 3 (0)	große Bandbreite , von topfit bis Grad5.<br><br>D-234: 3 - 3 (0)<br><br>Man muss unterscheiden zwischen Chronologischen und Physiologischen Alter,<br><br>D-367: 3 - 3 (0)<br><br>keine Unterschiede<br><br>D-432: 3 - 3 (0)
Erhöhter Behandlungs-bedarf			mehr paraodontale Erkrankung<br><br>D-416: 4 - 4 (0)<br><br>mehr Wurzelkaries<br><br>D-416: 5 - 5 (0)<br><br>Viele ältere brauchen auch Chirurgie, das ist kein einfacher Job in der Durchführung.<br>Kleinteiliger arbeiten, Prinzip der Zumutbarkeit gewährleisten.<br><br>D-420: 9 - 10 (0)<br><br>Die Zahnstaten und die Mundgesundheit in den Pflegeheimen die ich kenne lassen sehr (!) zu wünschen übrig.

	Specialists	General practitioners	Dentists
			D-420: 30 - 30 (0)<br><br>Prohylaktische u.knservierende Behandlungen viele ZE Reparaturen wenige ZE Neuanfertigungen<br><br>D-436: 3 - 3 (0)<br><br>Insgesamt sehen wir , das die Mehrzahl der Ü80 jährigen eigene Zähne hat.<br><br>D-439: 5 - 5 (0)
Wahrgen. Perspektive d. ü80 auf Gesundheits-zustand & Behandlung	Ihre Sicht in diagnostische Maßnahmen ist oft kritisch.<br><br>S-175: 4 - 4 (0)<br><br>Falscher Anspruch an Medizin und Gesundheitssystem<br><br>S-232: 5 - 5 (0)	Erkrankungen werden hingenommen, oftmals keine Diskussionen über Ursachen oder Veränderung: Ach, ich hab doch mein Leben gelebt...<br><br>GP-198: 3 - 3 (0)<br><br>oft fehlendes Krankheitsbewusstsein,<br><br>GP-201: 3 - 3 (0)<br><br>Manche falsche Vorstellung über Krankheit ist nicht mehr auszuräumen<br><br>GP-202: 13 - 13 (0)<br><br>ältere Patienten haben einen höheren Redebedarf oft mit weniger Anspruch auf medizinische Versorgung und Verbesserungen, sondern mehr Psychohygiene und Erhalt des status idem, Bestätigung, dass alles gut so ist, sich auch nicht mehr unbedingt verbessern läßt ("das ist halt das Alter")	Die Einschätzung des Patienten in Bezug auf seine Allgemeingesundheit, allgemeinen Fähigkeiten (z.B. Aktivitäten des täglichen Lebens, manuelle und kognitive Geschicklichkeit, Lebensform) spielt auch in in die zahnmedizinischer Therapieplanung mit hinein.<br><br>D-371: 4 - 4 (0)<br><br>Man sieht sich oft mit der Aussage konfrontiert: das lohnt sich in meinem Alter doch nicht mehr.<br><br>D-398: 3 - 3 (0)<br><br>Weniger Nachfragen über Hintergrund der Behandlung, häufiger Argumentation, dass sich Behandlung in ihrem Alter nicht mehr lohnt.<br><br>D-399: 3 - 3 (0)<br><br>Unsicher, ob Therapie noch lohnt<br><br>D-411: 4 - 4 (0)

	Specialists	General practitioners	Dentists
		GP-245: 3 - 3 (0)<br><br>große Hilfe wäre mehr Realismus bei Pat., die sich selbst durch die "rosa-rote Brille" wahrnehmen<br><br>GP-353: 13 - 13 (0)	Angst, die Behandlung nicht zu schaffen<br><br>D-411: 5 - 5 (0)<br><br>Patientn sagen oft, das lohnt doch nicht mehr.<br>Daher auf einfachere, medizinisch sinnvolle Basisversorgung zurückgreifen.<br><br>D-420: 7 - 8 (0)<br><br>Diese Patientengruppe glaubt oft,dass eine Behandlung nicht mehr sinnvoll ist,wegen des fortgeschrittenen Alters.<br><br>D-427: 3 - 3 (0)
Paternalistische Orientierung		Ältere Patienten brauchen Zeit, sie sind oft langsamer und sehr oft ist der Arzt bei diesem Klientel immer noch eine Respektsperson.<br>GP-198: 5 - 5 (0)<br><br>Viele Patienten sind weniger kritisch und tun, was man ihnen empfiehlt<br><br>GP-202: 6 - 6 (0)<br><br>Auch erlebe ich seit der impft Diskussion gegen Corona eher mal Misstrauen gegenüber der ärztlichen Beratung. Das Vertrauensverhältnis war vorher eher besser. Ich führe das auf die allgemeine Kommunikation in den Medien zurück. Manchmal wirkt das Argument, dass die Patienten früher doch auf meinen ärztlichen Rat vertraut haben, doch überzeugend.<br><br>GP-219: 3 - 3 (0)<br><br>paternalistisch, was auch gern angenommen wird<br><br>GP-353: 3 - 3 (0)	

	Specialists	General practitioners	Dentists
Einsicht und Compliance		Viele Patienten sind weniger kritisch und tun, was man ihnen empfiehlt<br><br>GP-202: 6 - 6 (0)<br><br>Auch erlebe ich seit der impft Diskussion gegen Corona eher mal Misstrauen gegenüber der ärztlichen Beratung. Das Vertrauensverhältnis war vorher eher besser. Ich führe das auf die allgemeine Kommunikation in den Medien zurück. Manchmal wirkt das Argument, dass die Patienten früher doch auf meinen ärztlichen Rat vertraut haben, doch überzeugend.<br><br>GP-219: 3 - 3 (0)<br><br>häufiger beratungsresistent<br>schlechtere Compliance<br><br>GP-512: 5 - 6 (0)	-Viele Grunderkrankungen bei fehlendem Verständnis für medizinischen Zusammenhang mit Zahnmed.<br><br>D-189: 6 - 6 (0)<br><br>mehr Zeit investieren wegen bestehender Defizite (schlechteres Sehen, hören verminderte Compliance)<br><br>D-383: 4 - 4 (0)<br><br>Compliance sehr eingeschränkt;<br><br>D-384: 3 - 3 (0)<br><br>gute Compliance<br><br>D-434: 3 - 3 (0)
**Interaktion und Behandlung**			
Beziehungs-aufbau und Vertrauen	mehr Vertrauen<br><br>S-231: 3 - 3 (0)	Die Patienten freuen sich über persönliche Zuwendung, ein persönliches Gespräch abseits der Erkrankungen; sei es durch mich oder meine Mitarbeiterinnen.<br><br>GP-198: 3 - 3 (0)<br><br>Bei Demenzpatienten ist mehr Gedulgd und Einfühlungsvermögen erforderlich<br><br>GP-202: 4 - 4 (0)<br><br>Betreuung über Jahre, dann oft plötzliche Veränderungen (Kognition/Mobilität); gute Arzt-Patienten-Bindung, lange dauerndes Vertrauensverhältnis; Begleitung über die Jahre duch verschidene Lebens-/Gesundheitskrisen;<br><br>GP-518: 3 - 3 (0)	Die Patienten sind in derRegel schon Jahrzehnte kontinuierlich in Behandlung, regelmäßige Untersuchungen und Prävetion; großes Vertrauen<br><br>D-362: 3 - 3 (0)<br><br>gutes miteinander<br><br>D-397: 3 - 3 (0)<br><br>Trotzdem ist diese Patientengruppe gut zu betreuen, gerade, wenn man die Menschen seit vielen Jahren kennt. Sie sind sehr dankbar.<br><br>D-521: 5 - 5 (0)
Geschwindigkeit und Zeit	benötigen oft mehr Zeit,<br><br>S-163: 3 - 3 (0)<br><br>Die Pat. sind nicht so mobil und benörigen mehr Zeit,	Alles viel langwieriger , umständlicher.<br><br>GP-192: 3 - 3 (0)	Die Arbeit mit den über 80 jährigen trägt zur Entschleunigung unserer Praxisabläufe bei. Denn automatisch (müssen) wir uns dem Tempo dieses Patientenkollektivs anpassen. Gibt es Einschränkungen: Mobilität, kognitiv, Hören?, Sehen?<br><br>D-371: 3 - 3 (0)

**Specialists**	**General practitioners**	**Dentists**
S-175: 3 - 3 (0)<br><br>in Teilen verlangsamt, behäbiger<br><br>S-221: 3 - 3 (0)<br><br>Die Patientinnen und Patienten (bitte nicht Patient*innen, das ist für beide Geschlechter diskriminierend) sind aufgrund zunehmender geistiger und körperlicher Immobilität langsamer als andere und benötigen daher mehr Zeit und Hilfe.<br><br>S-223: 3 - 3 (0)<br><br>mehr Zeitaufwand,<br><br>S-231: 3 - 3 (0)<br><br>Langsam, umständlicher<br><br>S-232: 3 - 3 (0)<br><br>mehr nachfragen, mehr Zeit für Beratung.<br><br>S-232: 3 - 3 (0)<br><br>Redebedarf - mehr Zeit<br><br>S-322: 3 - 3 (0)<br><br>meist schwerhörige Patienten , ich benötige mehr Zeit für Erläuterungen ,<br><br>S-325: 3 - 3 (0)<br><br>Über 80 Jährige sind deutlich langsamer in ihren Bewegungen, verstehen schlechter, können sich öfters die Informationen von mir nicht merken, sind manchmal auch dement.<br><br>S-372: 3 - 3 (0)	Ältere Patienten brauchen Zeit, sie sind oft langsamer und sehr oft ist der Arzt bei diesem Klientel immer noch eine Respektsperson.<br><br>GP-198: 5 - 5 (0)<br><br>Manche Erklärung dauert länger, Untersuchungen dauern länger, weil das An- und Ausziehen manchmal länger dauert<br><br>GP-202: 3 - 3 (0)<br><br>Komplexe Fälle und geringe Geschwindigkeit (körperlich aber auch mental) mit größerer Zeitaufwand<br><br>GP-244: 4 - 4 (0)<br><br>zeitaufwendig<br><br>GP-253: 3 - 3 (0)<br><br>und durch oft durch handycaps langsamer<br><br>GP-271: 3 - 3 (0)<br><br>im Verlauf dann oft sehr zeitintensiv durch hohen Betreuungsbedarf, Medikation, Pflegedienste, Physiotherapeuten, Pflegestufen; palliative Begleitung, Kommunikation mit Angehörigen.<br><br>GP-518: 3 - 3 (0)<br><br>oft langsamere Kommunikation<br><br>GP-520: 5 - 5 (0)<br><br>Neben budgetären Besonderheiten (wegen der fehlenden oder eingeschränkten Mobilität muss hausärztliche Medizin oft auch hochpreisige Medikamente verordnen, die sonst durch ein Facharztbudget gedeckt würden) spielt der Faktor Zeit in der Praxis eine große Rolle.<br><br>GP-525: 4 - 4 (0)	Erfordert viel Geduld, differenzierte Lagerung, viele Pausen und viel Rücksicht.<br><br>D-374: 3 - 3 (0)<br><br>Im Gegensatz zu Rest des Patientenpools zeitlich aufwändiger und psychologisch schwierigerer Umgang.<br><br>D-374: 3 - 3 (0)<br><br>mehr Zeit investieren wegen bestehender Defizite (schlechteres Sehen, hören verminderte Compliance)<br><br>D-383: 4 - 4 (0)<br><br>Man benötigt häufig mehr Zeit um Sachverhalte zu erklären.<br><br>D-398: 3 - 3 (0)<br><br>Man braucht mehr Zeit.<br><br>D-420: 3 - 3 (0)<br><br>Mehr Geduld bei der Behandlung.<br><br>D-423: 3 - 3 (0)<br><br>Ruhe ausstrahlen, viel Zeit,<br><br>D-434: 3 - 3 (0)<br><br>Wir benötigen mehr Zeit und Aufklärung<br><br>D-435: 3 - 3 (0)<br><br>Bei den Dementzpatienten geht es nicht immer reibungslos und wir benötigen sehr viel Zeit für die Behandlungen, was leider nicht honoriert wird.<br><br>D-439: 3 - 3 (0)<br><br>Zeitintensiver<br><br>D-454: 4 - 4 (0)

	Specialists	General practitioners	Dentists
	Man muss langsamer und deutlicher mit ihnen sprechen, auch wenn sie nicht hochgradig schwerhörig sind. Tests (z.B. Audiogramme) gehen deutlich erschwerter und langsamer.<br><br>S-372: 3 - 3 (0)<br><br>Zeitaufwendiger,<br><br>S-470: 3 - 3 (0)<br><br>längere Anamnesegespräche, längerer Untersuchnungszeiten,<br><br>S-470: 3 - 3 (0)	die Gruppe der über 80 jährigen ist aufgrund der Multimorbidität und der reduzierten Aufffassungsgabe sehr beratungs- und zuwendungsintensiv<br><br>GP-158: 3 - 3 (0)	Ich betreue ein Pflegeheim und suche dies regelmäßig auf.<br>Die Arbeit mit Hochbetagten ist zeitintensiver, oft verbunden mit Rückfragen an Betreuer oder Angehöhrige.<br><br>D-521: 3 - 4 (0)
Kontinuität und Kontrollen		Bei sehr schweren Krankheiten wird immer<br>der Wunsch nach Begleitung durch die Hausärztin geäußert.<br><br>GP-198: 3 - 4 (0)<br><br>Betreuung über Jahre, dann oft plötzliche Veränderungen (Kognition/Mobilität); gute Arzt-Patienten-Bindung, lange dauerndes Vertrauensverhältnis; Begleitung über die Jahre duch verschidene Lebens-/Gesundheitskrisen;<br><br>GP-518: 3 - 3 (0)	Die Patienten sind in derRegel schon Jahrzehnte kontinuierlich in Behandlung, regelmäßige Untersuchungen und Prävetion; großes Vertrauen<br><br>D-362: 3 - 3 (0)<br><br>Der Erfolg spricht aber für sich, durch die regelmäßige Kontrolle und Behandlung<br>in den Heimen, hat sich Anzahl der Füllungen bei den Patienten deutlich reduziert.<br><br>D-439: 3 - 4 (0)
Unterstützungs-bedarf	Hilfe bei An-und Ausziehen<br><br>S-470: 3 - 3 (0)	sind oft einsam, brauchen tips und Hilfe für Alltagsfragen (wie und wo kann ich Menschen kennenlernen oder Hilfe bekommen? wer hlft mir meinen Fernseher zu programmieren? Finden sich im Internet nicht zurecht, wissen nicht wie Pflegstufen zu beantragen sind, finden Telefonnummern für die kranaknekasse nicht...)<br><br>GP-506: 3 - 3 (0)<br><br>oft müssen wir solche Dinge, die eher oragnisatorisch sind übernehmen, wie Pfegedienste kontaktieren und planen, Altenheime anrufen, Sozialdienste der Stadt informieren etc.<br><br>GP-506: 3 - 3 (0)	Prophylaxeunterstützung, da sie allein häufig nicht mehr durchgeführt werden kann im Alltag.<br><br>D-383: 3 - 3 (0)

	Specialists	General practitioners	Dentists
Zusätzliche Stakeholder und Akteure	mehr Begleitpersonen<br><br>S-163: 3 - 3 (0)<br><br>Viele kommen in Begleitung, mit d. Wunsch die Selbstständigekit und Beweglichkeit mit Hilfe von Krankengymnastik aufrecht zu erhalten.<br><br>S-222: 3 - 3 (0)<br><br>Demente Patienten sind zumeist in Begleitung ihrer Angehörigen oder Pflegenden.<br><br>S-226: 6 - 6 (0)<br><br>Häufig Mitberatung von Angehörigen<br><br>S-232: 3 - 3 (0)<br><br>Kontrolluntersuchungen schwieriger, da oft begelitperson erfoderlcih/mobilitätseinschränkung, Schwerhörigkeit, Vergeßlichkeit<br><br>S-470: 3 - 3 (0)	Mehr Erklärung, soziale Versorgung, Angehörigenkontakte erforderlich<br><br>GP-192: 4 - 4 (0)<br><br>im Verlauf dann oft sehr zeitintensiv durch hohen Betreuungsbedarf, Medikation, Pflegedienste, Physiotherapeuten, Pflegestufen; palliative Begleitung, Kommunikation mit Angehörigen.<br><br>GP-518: 3 - 3 (0)	Es stellt sich schnell die Frage im Hinterkopf: muss ich alle relevanten Fragen nur mit dem Patienten oder mit weiteren Personen (Angehörigen/ Betreuern etc.) besprechen.<br><br>D-371: 4 - 4 (0)<br><br>Utopische Anforderungen der Angehörigen<br><br>D-374: 3 - 3 (0)<br><br>Absprache mit Pflegepersonal gut.<br><br>D-374: 3 - 3 (0)<br><br>Begleitpersonen / Betreuer / Heim in Behandlung einbinden<br><br>D-384: 3 - 3 (0)<br><br>Aufklärung der Angehörigen<br><br>D-407: 3 - 3 (0)<br><br>Sehr hilfreich ist die Anwesenheit einer Bezugsperson (Angehörige oder Pflegekräfte)<br><br>D-427: 3 - 3 (0)<br><br>Ich betreue ein Pflegeheim und suche dies regelmäßig auf.<br>Die Arbeit mit Hochbetagten ist zeitintensiver, oft verbunden mit Rückfragen an Betreuer oder Angehöhrige.<br><br>D-521: 3 - 4 (0)

	Specialists	General practitioners	Dentists
Kommunikation	ihre Auffassungsgabe ist manchmal schlechter und auch ihr Gehörsinn und ihr Sehvermögen.<br><br>S-175: 3 - 3 (0)<br><br>Eininige wenige haben viel Gesprächsbedarf und freuen sich, wennn ich einfach zuhöre.<br><br>S-222: 3 - 3 (0)<br><br>mehr nachfragen, mehr Zeit für Beratung.<br><br>S-232: 3 - 3 (0)<br><br>meist schwerhörige Patienten , ich benötige mehr Zeit für Erläuterungen ,<br><br>S-325: 3 - 3 (0)<br><br>Über 80 Jährige sind deutlich langsamer in ihren Bewegungen, verstehen schlechter, können sich öfters die Informationen von mir nicht merken, sind manchmal auch dement.<br><br>S-372: 3 - 3 (0)<br><br>Man muss langsamer und deutlicher mit ihnen sprechen, auch wenn sie nicht hochgradig schwerhörig sind. Tests (z.B. Audiogramme) gehen deutlich erschwerter und langsamer.<br><br>S-372: 3 - 3 (0)	Die Patienten freuen sich über persönliche Zuwendung, ein persönliches Gespräch abseits der Erkrankungen; sei es durch mich oder meine Mitarbeiterinnen.<br><br>GP-198: 3 - 3 (0)<br><br>Bei Demenzpatienten ist mehr Gedulgd und Einfühlungsvermögen erforderlich<br><br>GP-202: 4 - 4 (0)<br><br>oft langsamere Kommunikation<br><br>GP-520: 5 - 5 (0)<br><br>wegen Einsamkeit oft besondere Kommunikationswünsche<br><br>GP-520: 6 - 6 (0)<br><br>schwerer, Sachverhalter darzulegen<br><br>GP-512: 4 - 4 (0)	Sehr viel Aufklärungs- und Recherchebedarf nötig vor während und nach Behandlungen.<br>D-234: 3 - 3 (0)<br><br>Im Gegensatz zu Rest des Patientenpools zeitlich aufwändiger und psychologisch schwierigerer Umgang.<br>D-374: 3 - 3 (0)<br><br>Mangelnde und nur sehr schwere Absprache mit HA zu Medikation, häufig demenzielle Veränderungen machen Aufklärung schwer.<br>D-374: 3 - 3 (0)<br><br>Man benötigt häufig mehr Zeit um Sachverhalte zu erklären.<br>D-398: 3 - 3 (0)<br><br>Erklärungen in einfachen Worten auf den Punkt gebracht sind ratsam.<br>D-420: 4 - 4 (0)<br><br>Intensivere Aufklärung über Mundhygiene.<br>D-423: 3 - 3 (0)<br><br>Schwerhörigkeit und Demenz machen es manchmal schwer, mit dem Pat. zu kommunizieren und das erschwert die Mitarbeit. Aber im Prinzip ist die Behandlung ähnlich zu der Behandlung kleiner Kinder.<br>D-433: 3 - 3 (0)<br><br>Langsam und deutlich reden,<br>D-434: 3 - 3 (0)<br><br>simple Erklärungen<br>D-434: 3 - 3 (0)<br><br>Wir benötigen mehr Zeit und Aufklärung<br>D-435: 3 - 3 (0)<br><br>erschwertes Verständnis,<br>D-449: 3 - 3 (0)<br><br>altersspezifische Kommunikation<br>D-451: 3 - 3 (0)

	Specialists	General practitioners	Dentists
Behandlungs-konzepte	Individuell auf sie eingehen<br><br>S-469: 3 - 3 (0)	Mehr Erklärung, soziale Versorgung, Angehörigenkontakte erforderlich<br><br>GP-192: 4 - 4 (0)<br><br>oftmals Lösen von Versorgungs-/Alltagsproblemen<br><br>GP-253: 5 - 5 (0)<br><br>Vielfach Sozialanwalt und Versorgungsstrukturierer und weniger Medizin<br><br>GP-508: 3 - 3 (0)<br><br>im Verlauf dann oft sehr zeitintensiv durch hohen Betreuungsbedarf, Medikation, Pflegedienste, Physiotherapeuten, Pflegestufen; palliative Begleitung, Kommunikation mit Angehörigen.<br><br>GP-518: 3 - 3 (0)<br><br>Geriatrische Pat. sind anspruchsvoll, geriatrische Medizin ist MITNICHTEN ein Herunterbrechen der Therapie auf den alten Menschen.<br><br>GP-525: 4 - 4 (0)<br><br>Geriatrie ist mehr als Medikamente verordnen, die den Mediplan ausufern lassen. Es ist ein sehr individualisierter Zweig der Medizin, der den alten Menschen mit seinen individuellen Gebrechen/Einschränkungen auffängt und bestehen lässt.<br><br>GP-525: 16 - 16 (0)	Brauchen aber in der Versorgung häufig inidividuelle Behandlungen, und es muss auf die inidivuelle Situation mehr eingegangen werden<br><br>D-367: 5 - 5 (0)<br><br>Die Einschätzung des Patienten in Bezug auf seine Allgemeingesundheit, allgemeinen Fähigkeiten (z.B. Aktivitäten des täglichen Lebens, manuelle und kognitive Geschicklichkeit, Lebensform) spielt auch in in die zahnmedizinischer Therapieplanung mit hinein.<br><br>D-371: 4 - 4 (0)<br><br>seniorengerechte Therapie, Minimaltherapie (Altenheim)<br><br>D-376: 3 - 3 (0)<br><br>Schmerzfreiheit steht an erster Stelle<br><br>D-383: 3 - 3 (0)<br><br>keine komplizierte Prothetik - einfaches Handling ist hier erste Wahl; konservierend - chirurgisch so wenig wie möglich und soviel wie nötig, abhängig vom Pflegegrad und der geistigen und körperlichen Fitness;<br><br>D-384: 3 - 3 (0)<br><br>nur im allernötigsten Fall Überweisung an MGK - Chirurgie - möglichst alles vor Ort behandeln;<br><br>D-384: 3 - 3 (0)<br><br>keine langen Termine;<br><br>D-384: 3 - 3 (0)<br><br>umfangreichere Betreuung und Behandlungsbegleitung,<br><br>D-407: 3 - 3 (0)<br><br>Fast jede Behandlung ist schwieriger und anstrengender.<br>Limitierende Faktoren sind u.a. körperliche Einschränkungen und damit verbundene Lagerungsschwierigkeiten.<br><br>D-420: 5 - 6 (0)

	Specialists	General practitioners	Dentists
			Patientn sagen oft, das lohnt doch nicht mehr.<br>Daher auf einfachere, medizinisch sinnvolle Basisversorgung zurückgreifen.<br><br>D-420: 7 - 8 (0)<br><br>Viele ältere brauchen auch Chirurgie, das ist kein einfacher Job in der Durchführung.<br>Kleinteiliger arbeiten, Prinzip der Zumutbarkeit gewährleisten.<br><br>D-420: 9 - 10 (0)<br><br>Aber bei Schmerzen sind Sie sehr dankbar für eine schnelle Behandlung.<br><br>D-427: 3 - 3 (0)<br><br>Prohylaktische u.knservierende Behandlungen viele ZE Reparaturen wenige ZE Neuanfertigungen<br><br>D-436: 3 - 3 (0)<br><br>Der Erfolg spricht aber für sich, durch die regelmäßige Kontrolle und Behandlung<br>in den Heimen, hat sich Anzahl der Füllungen bei den Patienten deutlich reduziert.<br><br>D-439: 3 - 4 (0)<br><br>altersgerechte Therapiekonzepte, Laufzeit, Nachhaltigkeit, Notwendigkeit, Sinnigkeit, Lebensqualität<br><br>D-451: 6 - 6 (0)
Strukturelle und organisatorische Besonderheiten		schlechte soziale Anbindung/Versorgung mit vielen organisatorischen Problemen, worunter auch die Patienten leiden.<br><br>GP-244: 5 - 5 (0)	Pflege hat defintiv zu wenig Zeit für Mundhygiene. Es gibt keine vorsorgliche Anpassung der demografischen Veränderung und dem damit verbundenen Mehraufwand für die Ärzte. Equipment ist sehr kostenintensiv. Dies sollte gedeckelt werden!<br><br>D-374: 3 - 3 (0)

	Specialists	General practitioners	Dentists
		Vielfach Sozialanwalt und Versorgungsstrukturierer und weniger Medizin<br><br>GP-508: 3 - 3 (0)<br><br>Aufgrund des Standorts meiner Praxis und der Hartleibigkeit der Stadt, in der ich niedergelassen bin, findet meine geriatrische Versorgung zum größten Teil in Heimen statt. Ich kann leider keine Barrierefreiheit ermöglichen, was mich sehr traurig macht.<br><br>GP-525: 3 - 3 (0)<br><br>Neben budgetären Besonderheiten (wegen der fehlenden oder eingeschränkten Mobilität muss hausärztliche Medizin oft auch hochpreisige Medikamente verordnen, die sonst durch ein Facharztbudget gedeckt würden) spielt der Faktor Zeit in der Praxis eine große Rolle.<br><br>GP-525: 4 - 4 (0)<br><br>sind oft einsam, brauchen tips und Hilfe für Alltagsfragen (wie und wo kann ich Menschen kennenlernen oder Hilfe bekommen? wer hlft mir meinen Fernseher zu programmieren? Finden sich im Internet nicht zurecht, wissen nicht wie Pflegstufen zu beantragen sind, finden Telefonnummern für die kranaknekasse nicht...)<br><br>GP-506: 3 - 3 (0)<br><br>oft müssen wir solche Dinge, die eher oragnisatorisch sind übernehmen, wie Pfegedienste kontaktieren und planen, Altenheime anrufen, Sozialdienste der Stadt informieren etc.<br><br>GP-506: 3 - 3 (0)	häufig Rücksprachen mit Hausärzten.<br><br>D-396: 3 - 3 (0)<br><br>Konsil mit HA<br><br>D-407: 3 - 3 (0)<br><br>Organisation von Labordiensten<br><br>D-407: 3 - 3 (0)<br><br>Wir arbeiten in 5 Altenheimen. Die Patienten sind überwiegend glücklich , das sie in den Heimen behandelt werden und nicht in die Praxis gebracht werden müssen .<br><br>D-439: 3 - 3 (0)<br><br>Ich betreue ein Pflegeheim und suche dies regelmäßig auf.<br>Die Arbeit mit Hochbetagten ist zeitintensiver, oft verbunden mit Rückfragen an Betreuer oder Angehöhrige.<br><br>D-521: 3 - 4 (0)
Individuelle, personen-zentrierte Sichtweise und Behandlung	symptomorient, rational das was nötig ist ohne Maximalismus oder erhobenen Zeigefinger<br><br>S-175: 5 - 5 (0)<br><br>von der stationären Geriatire wünsche ich mir mehr Menschlichkeit, mehr Individualität und eine bessere Angehärigenarbeit<br><br>S-196: 7 - 7 (0)	Anpassung der Medikation an Alter, Nierenfunktion, Machbarkeit,<br><br>GP-192: 5 - 5 (0)<br><br>Modifikation der Therapieziele<br><br>GP-192: 6 - 6 (0)<br><br>Keine blinde Befolgung von Leitlinien.	-Individuelle medizinische Versorgung und Betreuung! Nicht über einen Kamm scheren (Leitlinien und Enpfehlungen an die man sich zunehmend aus rechtlichen Gründen halten muss machen dies immer schwieriger)<br><br>D-189: 12 - 12 (0)<br><br>Menschorientiert, zuwendend, adäquat<br><br>D-234: 4 - 4 (0)

**Specialists**	**General practitioners**	**Dentists**
Ausreichende Zeitfenster zum "Kümmern", Befragen, Untersuchen und Behandeln.<br><br>S-223: 4 - 4 (0)<br><br>Im Vordergrund steht die geistige/mentale Gesundheit - die bestimmt zumeist auch die körperliche.<br><br>S-226: 7 - 7 (0)<br><br>"sprechende Medizin" ist bei sehr alten Menschen auch im fachärztlichen Bereich wichtig und zu unterstützen/honorieren<br><br>S-226: 18 - 18 (0)<br><br>individuell angepasste Therapiekonzepte, weniger Leitlinienmedizin<br><br>S-232: 4 - 4 (0)<br><br>Eine dem AZ und den Wünschen des Patienten angemessene Diagnostik und Therapie<br><br>S-516: 4 - 4 (0)<br><br>Alle Comorbiditäten sollen berücksichtigung findet. Daher ist eine enge Kooperatiion aller Therapeuten wünschenswert.<br><br>S-516: 4 - 4 (0)<br><br>Ich glabe das Insgesamt in dieser Altersgruppe ein Zuviel an "Medizin" zur Anwendung kommt. Lebensqualität und Lebenserwartung in dieser Personengruppe erfährt zu wenig Berücksichtigung. Gerade im fachärztliche Bereich sind die Kollegen zu sehr auf Ihren Fachbereich fokussiert die Gesamtsituation findet so deutlich zu wenig Beachtung.<br><br>S-516: 13 - 13 (0)	GP-192: 7 - 7 (0)<br><br>Individualisierte Medizin<br><br>GP-192: 8 - 8 (0)<br><br>Die Patienten sollten als Person und nicht als Krankheitsfall betrachtet werden.<br><br>GP-198: 5 - 5 (0)<br><br>Hilfestellung bei zunehmend eingeschränkter Seh– Und HörFähigkeit, bei Mentalen Einschränkungen.<br><br>GP-219: 4 - 4 (0)<br><br>zuhören und den Patient und seine Bedürfnisse in den Mittelpunkt stellen und nicht die medizinischen Leitlinien<br><br>GP-245: 4 - 4 (0)<br><br>Soviel wie nötig<br>So wenig wie möglich<br><br>GP-275: 4 - 5 (0)<br><br>Gezielte, sich auf das Notwendige beschränkende Diagnostik und rationale medikamentöse Therapie/Mut zum Downprescribing.<br><br>GP-525: 5 - 5 (0)<br><br>Akzeptanz der Wünsche und Erwartungen des alten Menschen im Hinblick auf das noch zu erreichende Lebensziel.<br><br>GP-525: 5 - 5 (0)<br><br>Berücksichtigung Co-Morbiditäten<br><br>GP-512: 7 - 7 (0)<br><br>- Berücksichtigung sozialer Situation/Versorgung	Volle Aufmerksamkeit<br><br>D-305: 3 - 3 (0)<br><br>Muss mi der Petsönlichkeit des Patienten, seinen Erwartungen undPersptiven in Übereinstimmung sein, vorausschauend planen, Respekt<br><br>D-362: 4 - 4 (0)<br><br>Gute Basispflege und Grundkonzept.<br><br>D-374: 4 - 4 (0)<br><br>Liebevoller und zeitlich angepasster Umgang, ohne dass der Arzt einen wirtschaftlichen Verlust erleidet. Rückschluss: Honorar muss angepasst werden. Dies auch bei Patienten, die nicht in einer SGV XI Einrichtung leben, sondern in häuslicher Betreuung sind.<br><br>D-374: 4 - 4 (0)<br><br>Zeit!!! Zuhören, begleiten, unterstützen<br><br>D-395: 6 - 6 (0)<br><br>Am Wohl und Zustand des Patienten ausgerichtete Therapien, individuell modifiziert<br><br>D-411: 7 - 7 (0)<br><br>mehr funktionelle Behandlung, als ästhetische Behandlung<br><br>D-416: 10 - 10 (0)<br><br>gute ganzheitliche Betreuung<br><br>D-416: 11 - 11 (0)<br><br>Eine Versorgung die den Möglichkeiten der älteren Patienten gerecht wird in Bezug auf Herstellung und der täglichen Pflege<br><br>D-435: 4 - 4 (0)<br><br>Ein wertschätzender Umgang mit alten Menschen. Dabei sollte nicht die beste zahnmedizinische Versorgung, sondern die für den Patienten beste

	**Specialists**	**General practitioners**	**Dentists**
		GP-524: 6 - 6 (0)	Versorgung im Vordergrund stehen. Denn nicht alles, was zahnmedizinisch möglich ist, ist am Ende des Lebens auch sinnvoll.<br><br>D-447: 6 - 6 (0)
Patient:innen-relevante Outcomes	Viele kommen in Begleitung, mit d. Wunsch die Selbstständigekit und Beweglichkeit mit Hilfe von Krankengymnastik aufrecht zu erhalten.<br><br>S-222: 3 - 3 (0)<br><br>Im Vordergrund steht die geistige/mentale Gesundheit - die bestimmt zumeist auch die körperliche.<br><br>S-226: 7 - 7 (0)<br><br>"sprechende Medizin" ist bei sehr alten Menschen auch im fachärztlichen Bereich wichtig und zu unterstützen/honorieren<br><br>S-226: 18 - 18 (0)	Modifikation der Therapieziele<br><br>GP-192: 6 - 6 (0)<br><br>Erhalt der Eigenständigkeit so lange es geht!<br><br>GP-201: 4 - 4 (0)<br><br>Patient sollte über seine Gesundheit bescheid wissen, seine Krankheiten und ihre Auswirkungen kennen<br><br>GP-202: 7 - 7 (0)<br><br>bestmögliche Erhaltung der Lebensqualität<br><br>GP-353: 4 - 4 (0)<br><br>Erhalt der Autonomie gewährleisten<br><br>GP-508: 4 - 4 (0)<br><br>die älteren Damen sind oft geschockt, wenn sie vom Gynäkologen weggeschickt werden mit den Worten "Was wollen S I E denn in diesem Alter noch bei mir?" Regelm. 5 Sinne-Diagnostik sehr effektiv, was z.B. beg. Herzinsuffizienz, Niernerkr., Osteorose etc. angehen; früh einsetzendes adäquates Management erhält m.E. oft lange eine hohe Lebensqualität.<br><br>GP-518: 4 - 4 (0)<br><br>Gezielte, sich auf das Notwendige beschränkende Diagnostik und rationale medikamentöse Therapie/Mut zum Downprescribing.<br><br>GP-525: 5 - 5 (0)<br><br>Akzeptanz der Wünsche und Erwartungen des alten Menschen im Hinblick auf das noch zu erreichende Lebensziel.	Oben an steht die Schmerzfreiheit. Kontrollorientierte Versorgung.<br><br>D-361: 4 - 4 (0)<br><br>Schmerzfreiheit erreichen, Bestehenden Zustand erhalten,<br><br>D-383: 5 - 5 (0)<br><br>für den zahnärztlichen Bereich: die Kaufunktion so einfach wie möglich wiederherstellen;<br><br>D-384: 4 - 4 (0)<br><br>keine hohen Belastungen;<br><br>D-384: 4 - 4 (0)<br><br>Kaufähigkeit wichtig<br><br>D-397: 4 - 4 (0)<br><br>Risiko-Nutzen Abwägung<br>" so wenig wie möglich, soviel wie nötig"<br><br>D-416: 8 - 9 (0)<br><br>mehr funktionelle Behandlung, als ästhetische Behandlung<br><br>D-416: 10 - 10 (0)<br><br>Parodontale Gesundheit ist das Wichtigste. Auch und für den allgemeinen Gesundheitszustand.<br><br>D-420: 11 - 11 (0)<br><br>Leben ohne Schmerzen;<br><br>D-422: 4 - 4 (0)<br><br>selbstbestimmtes Leben;

	Specialists	General practitioners	Dentists
		GP-525: 5 - 5 (0)<br><br>Motvation zu Eigenständigkeit,<br><br>GP-158: 4 - 4 (0)<br><br>Gesundheitskompetenz fördern als Grundlage für die Adhärenz in der notwenigen Therapie<br><br>GP-158: 5 - 5 (0)<br><br>Vermeidung Polymedikation<br>Verbesserung Sturzprävention<br><br>GP-512: 8 - 9 (0)<br><br>- Erhaltung der Eigenständigkeit<br><br>GP-524: 7 - 7 (0)<br><br>- Beachtung Ernährung/Mobilität<br><br>GP-524: 8 - 8 (0)	D-422: 4 - 4 (0)<br><br>Prävention vor Zahnverlust, hochwertige Prothetik für eine gute Kaufunktion.<br><br>D-423: 4 - 4 (0)<br><br>Die Schulung in den Pflegeheimen sollte vermitteln, dass ein gesundes Gebiß sehr viel Lebensqualität bedeutet. Schmerzfreiheit und gut essen zu können, wirkt sich auch auf die allgemeine Gesundheit und Grundstimmung aus. Weniger Entzündungen schützen auch andere Organsystheme.<br><br>D-427: 4 - 4 (0)<br><br>Wichtig ist die halbjährliche Kontrolle, da die Motorik nachlässt und die Mundhygiene nicht mehr ausreichend gewährleistet ist.<br><br>D-439: 6 - 6 (0)<br><br>Ein wertschätzender Umgang mit alten Menschen. Dabei sollte nicht die beste zahnmedizinische Versorgung, sondern die für den Patienten beste Versorgung im Vordergrund stehen. Denn nicht alles, was zahnmedizinisch möglich ist, ist am Ende des Lebens auch sinnvoll.<br><br>D-447: 6 - 6 (0)<br><br>Sicherung der Lebensqualität. Kaufunktion<br>Essen ist der Sex des Alters<br>D-451: 7 - 8 (0)
Zustand und Verhalten von Patient:innen		Patient sollte über seine Gesundheit bescheid wissen, seine Krankheiten und ihre Auswirkungen kennen<br><br>GP-202: 7 - 7 (0)<br><br>sollte compliant sein<br><br>GP-202: 10 - 10 (0)<br><br>Realistische Erwartungen.<br><br>GP-244: 6 - 6 (0)<br><br>Gesundheitskompetenz fördern als Grundlage für die Adhärenz in der notwenigen Therapie	Verständnis<br><br>D-367: 7 - 7 (0)<br><br>Eigene Fitness, gute Betreuung auf allen Ebenen.<br><br>D-396: 4 - 4 (0)

	Specialists	General practitioners	Dentists
		GP-158: 5 - 5 (0)	
Empathie und Wert-schätzung	von der stationären Geriatire wünsche ich mir mehr Menschlichkeit, mehr Individualität und eine bessere Angehärigenarbeit<br><br>S-196: 7 - 7 (0)<br><br>Empathie<br><br>S-221: 4 - 4 (0)	Empathie<br><br>GP-245: 4 - 4 (0)<br><br>Nicht als "alt" abstempeln;<br><br>GP-518: 4 - 4 (0)	Menschorientiert, zuwendend, adäquat<br><br>D-234: 4 - 4 (0)<br><br>Muss mi der Petsönlichkeit des Patienten, seinen Erwartungen undPersptiven in Übereinstimmung sein, vorausschauend planen, Respekt<br><br>D-362: 4 - 4 (0)<br><br>Liebevoller und zeitlich angepasster Umgang, ohne dass der Arzt einen wirtschaftlichen Verlust erleidet. Rückschluss: Honorar muss angepasst werden. Dies auch bei Patienten, die nicht in einer SGV XI Einrichtung leben, sondern in häuslicher Betreuung sind.<br><br>D-374: 4 - 4 (0)<br><br>Ein wertschätzender Umgang mit alten Menschen. Dabei sollte nicht die beste zahnmedizinische Versorgung, sondern die für den Patienten beste Versorgung im Vordergrund stehen. Denn nicht alles, was zahnmedizinisch möglich ist, ist am Ende des Lebens auch sinnvoll.<br><br>D-447: 6 - 6 (0)
Kommunikation	Zuhören.<br>S-222: 4 - 4 (0)<br><br>Zurückhaltung, Aufklärung,<br>S-231: 4 - 4 (0)<br><br>gute Aufklärung der Patienen , mehr Zeit ,<br>S-325: 4 - 4 (0)	Verständliche Sprache, Erklärungen, nachvollziehbare Therapien, so dass der Patienten in die Lage versetzt wird, seine Erkrankung zu verstehen.<br>GP-198: 5 - 5 (0)<br><br>Ich erlebe es immer wieder, dass Patienten mit einem Zettel in der Hand in meiner Praxis erscheinen, sei es aus dem Krankenhaus oder von Fachärzten, in völliger Unkenntnis der Dinge.<br>GP-198: 5 - 5 (0)<br><br>Es ist zu bemerken, dass nach Klärung aller Fragen, bei Wahrnehmung aller Sorgen sie sich sicher fühlen.<br>GP-198: 5 - 5 (0)<br><br>zuhören und den Patient und seine Bedürfnisse in den Mittelpunkt stellen und nicht die medizinischen Leitlinien<br>GP-245: 4 - 4 (0)<br><br>ausreichend Zeit für eine gute Beratung<br>GP-514: 4 - 4 (0)	Zeit!!! Zuhören, begleiten, unterstützen<br>D-395: 6 - 6 (0)<br><br>Geduld, alles erklären,<br>D-434: 4 - 4 (0)

	Specialists	General practitioners	Dentists
Zeit	zeitnahe, wohnortnahe, situationsadäquate Versorgung ohne Zeitdruck<br><br>S-163: 4 - 4 (0)<br><br>mehr zeit<br><br>S-217: 7 - 7 (0)<br><br>Ausreichende Zeitfenster zum "Kümmern", Befragen, Untersuchen und Behandeln.<br><br>S-223: 4 - 4 (0)<br><br>sich Zeit nehmen,<br><br>S-231: 4 - 4 (0)<br><br>Zeitbedarf<br><br>S-322: 4 - 4 (0)<br><br>gute Aufklärung der Patienen , mehr Zeit ,<br><br>S-325: 4 - 4 (0)	Zeit<br><br>GP-245: 4 - 4 (0)<br><br>Man muss sich als Arzt mehr Zeit nehmen.<br><br>GP-253: 6 - 6 (0)<br><br>ausreichend Zeit für eine gute Beratung<br><br>GP-514: 4 - 4 (0)	Zeit<br><br>D-189: 14 - 14 (0)<br><br>Zeit<br><br>D-367: 8 - 8 (0)<br><br>Liebevoller und zeitlich angepasster Umgang, ohne dass der Arzt einen wirtschaftlichen Verlust erleidet. Rückschluss: Honorar muss angepasst werden. Dies auch bei Patienten, die nicht in einer SGV XI Einrichtung leben, sondern in häuslicher Betreuung sind.<br><br>D-374: 4 - 4 (0)<br><br>Zeit!!! Zuhören, begleiten, unterstützen<br><br>D-395: 6 - 6 (0)<br><br>die Einsicht der zeit vor dem älter werden....<br><br>D-415: 4 - 4 (0)<br><br>Geduld, alles erklären,

	Specialists	General practitioners	Dentists
	Wichtig ist eine höhere Dichte an Ärzten, v.a. Hausärtzen, und diese sollten auch mehr Zeit haben, gründliche Diagnostiken durchzuführen.<br><br>S-372: 4 - 4 (0)<br><br>Zeitfaktor<br><br>S-469: 4 - 4 (0)		D-434: 4 - 4 (0)<br><br>gutes Zeitmanagement, man braucht mehr zeit und Geduld<br><br>D-521: 7 - 7 (0)
Voraus-schauende Versorgung	Verlässlichkeit<br><br>S-216: 5 - 5 (0)<br><br>Regelmäßige ärztliche Kontrollen, keine unnötigen Untersuchungen!!<br><br>S-222: 6 - 6 (0)<br><br>regelmäßige Kontrolluntersuchung<br><br>S-325: 4 - 4 (0)<br><br>Ich glabe das Insgesamt in dieser Altersgruppe ein Zuviel an "Medizin" zur Anwendung kommt. Lebensqualität und Lebenserwartung in dieser Personengruppe erfährt zu wenig Berücksichtigung. Gerade im fachärztliche Bereich sind die Kollegen zu sehr auf Ihren Fachbereich fokussiert die Gesamtsituation findet so deutlich zu wenig Beachtung.<br><br>S-516: 13 - 13 (0)	Erhalt der Eigenständigkeit so lange es geht!<br><br>GP-201: 4 - 4 (0)<br><br>Bei der Behandlung altersgerechte Zeitsplanung von allen mitbeteiligen(Versicherung, Pflegepersonal, Ärzte etc.)<br><br>GP-244: 8 - 8 (0)<br><br>Leichter Kontrolle der Therapie- Einnahme/ -Erfolg , Folgen bzw. Nebenwirkung etc.<br><br>GP-244: 9 - 9 (0)<br><br>regelmäßige Kontrolluntersuchungen und kostenlose Versorgung , Bewegungsangebote<br><br>GP-271: 4 - 4 (0)<br><br>die Hochbetagten lieben ihre "Vorsorgeuntersuchungen" (Koloskopie mal ausgenommen)<br><br>GP-518: 4 - 4 (0)<br><br>die älteren Damen sind oft geschockt, wenn sie vom Gynäkologen weggeschickt werden mit den Worten "Was wollen S I E denn in diesem Alter noch bei mir?" Regelm. 5 Sinne-Diagnostik sehr effektiv, was z.B. beg. Herzinsuffizienz, Niernerkr., Osteorose etc. angehen; früh	Prophylaxe und Kontrollen regelmäßig um die Mundhygiene zu unterstützen<br><br>D-361: 4 - 4 (0)<br><br>Oben an steht die Schmerzfreiheit. Kontrollorientierte Versorgung.<br><br>D-361: 4 - 4 (0)<br><br>Muss mi der Petsönlichkeit des Patienten, seinen Erwartungen undPersptiven in Übereinstimmung sein, vorausschauend planen, Respekt<br><br>D-362: 4 - 4 (0)<br><br>Die vorausschauende Planung in der Gesundheitsversorgung. Also kein komplizierter Zahnersatz, sondern einer, der sich auch später noch gut pflegen lässt ggf. auch von Personen, die die Mundpflege bei diesem Menschen übernehmen. Ein Zahnersatz der stabil und ausbaufähig ist.<br><br>D-371: 5 - 5 (0)<br><br>Verbesserte Mundhygiene, da dort viele Erkrankungen verstärkt werden.<br><br>D-374: 4 - 4 (0)<br><br>Aufklärung des Personals und der Angehörigen und Aufnahme der Mundhyigene in den Pflegestandard.<br><br>D-374: 4 - 4 (0)<br><br>Zahnpflege

Specialists	General practitioners	Dentists
	einsetzendes adäquates Management erhält m.E. oft lange eine hohe Lebensqualität.<br><br>GP-518: 4 - 4 (0)<br><br>klare und gemeinsam erstellte Angaben zu Kontrolluntersuchungen -Termine und Inhalte der Kontrollen<br><br>GP-158: 6 - 6 (0)	D-376: 4 - 4 (0)<br><br>in der Mundhygiene unterstützen<br><br>D-383: 5 - 5 (0)<br><br>regelmäßige Kontrolltermine mind. alle 6 Monate<br><br>D-384: 4 - 4 (0)<br><br>Regelmäßige Kontrolle der Gesundheitsverhältnisse.<br><br>D-399: 5 - 5 (0)<br><br>Unterstützung bei der Mundhygiene falls notwendig.<br><br>D-399: 5 - 5 (0)<br><br>Parodontale Gesundheit ist das Wichtigste. Auch und für den allgemeinen Gesundheitszustand.<br>Dazu bedarf es eines grossen Umschwungs, besonders in Pflegeeinrichtungen.<br>Es müssen neue Konzepte her, auch baulicher Art (eigener Behandlungsraum ZMK bei Neubau oder Renovierung !)<br><br>D-420: 11 - 13 (0)<br><br>Schulungen der Mitarbeiter. Regelmässige Prophylaxe-Intervalle mindesten alle vier Monate. Übernahme der Kosten durch GKV.<br><br>D-420: 14 - 14 (0)<br><br>Prävention vor Zahnverlust, hochwertige Prothetik für eine gute Kaufunktion.<br><br>D-423: 4 - 4 (0)<br><br>Die rechtzeitigen,regelmäßigen Vorsorgeuntersuchungen.<br><br>D-427: 4 - 4 (0)<br><br>Für mein Fach der Zahnmedizin ist die regelmäßige Prophylaxe sehr sinnvoll. Ebenso eine gut zu pflegende Prothetik.

Specialists	General practitioners	Dentists
		D-427: 4 - 4 (0)<br><br>Zahnpflege und Prophylaxe<br><br>D-432: 4 - 4 (0)<br><br>Und auch im hohne Alter sollte eine mind. jährliche Kontrolle der Zähne statt finden.<br><br>D-433: 4 - 4 (0)<br><br>Prophylaxe/Vorsorge<br><br>D-434: 4 - 4 (0)<br><br>Prophylaktische Maßnahmen zu Erhalt der ZMK Gesundheit<br><br>D-436: 4 - 4 (0)<br><br>Wichtig ist die halbjährliche Kontrolle, da die Motorik nachlässt und die Mundhygiene nicht mehr ausreichend gewährleistet ist.<br><br>D-439: 6 - 6 (0)<br><br>Prophylaxe<br><br>D-454: 6 - 6 (0)<br><br>kontrollen und Zahnreinigungen müssten vor Ort 6 mal im Jahr gemacht werden (Pflegepersonal schafft das nicht)<br>Mundhygienezustand ist katastrophal)<br><br>D-479: 5 - 6 (0)

	Specialists	General practitioners	Dentists
Zugang und Infra-struktur	zeitnahe, wohnortnahe, situationsadäquate Versorgung ohne Zeitdruck<br><br>S-163: 4 - 4 (0)<br><br>Bereitstellen einer Gesundheitsakte mit allen Informationen des Patienten wie Vorerkrankungen, Vor-OPs, Medikamente und Befunde von Untersuchungen<br><br>S-169: 4 - 4 (0)<br><br>wünscheswert wäre Unterstützung bei Mobiliät, um überhaupt Facharztbehandlugn möglich zu machen<br><br>S-196: 5 - 5 (0)<br><br>mehr ortsnahe Angebote und auch Angebote für Menschen, die nicht pflegebedürftig und dement sind<br><br>S-196: 6 - 6 (0)<br><br>Das Angebot von altersgerechte Gruppengymnastik trocken/Wasser.<br><br>S-222: 7 - 7 (0)<br><br>Medizinische Versorgung in der Nähe.<br><br>S-223: 5 - 5 (0)<br><br>Wichtig ist eine höhere Dichte an Ärzten, v.a. Hausärtzen, und diese sollten auch mehr Zeit haben, gründliche Diagnostiken durchzuführen.<br><br>S-372: 4 - 4 (0)	Meines Erachtens braucht es eine gute ärztliche Infrastruktur, gerade auf dem Lande.<br><br>GP-198: 5 - 5 (0)<br><br>Zugang zu notwendigen Therapien haben<br><br>GP-202: 8 - 8 (0)<br><br>regelmässige Kontrolluntersuchungen wahrnehmen können<br><br>GP-202: 9 - 9 (0)<br><br>regelmäßige Kontrolluntersuchungen und kostenlose Versorgung , Bewegungsangebote<br><br>GP-271: 4 - 4 (0)<br><br>zuverlässige hausärztliche Versorgung<br><br>GP-520: 8 - 8 (0)<br><br>Zugang zu allen Leistungen des Gesundheitssystems (Geriatrie, Physiotherapie, Psychotherapie)<br><br>GP-520: 9 - 9 (0)<br><br>mehr Zeit und Geld für HAusbesuche,<br><br>GP-506: 4 - 4 (0)	-Ausreichend Geldmittel für die meist sehr armen, älteren Menschen durch soziale Systeme besonders für den unterfinanzierten Bereich der Zahnmedizin, seit der Einführung des Festzuschusssystems (Stichwort: Patient kann sich trotz einer Krankenversicherung keine Basisversorgung (zB Prothese) leisten, weil der Eigenanteil bei kleiner Rente zu hoch ist, man aber trotzdem noch zu viel Einkommem hat um durch die sozialen Systeme die volle Kostenerstattung zu erhalten.)<br><br>D-189: 13 - 13 (0)<br><br>Erreichbarkeit<br><br>D-367: 6 - 6 (0)<br><br>Die Gesundheitsversorgung darf Menschen nicht ausschließen, wenn sie pflegebedürftig werden. Es müssen also Möglichkeiten gefunden werden, dass eine kontinuierliche medizinische Betreuung bis ins hohe Alter sicher gestellt ist sowohl in der Vorsorge, als auch bei Behandlungsbedarf<br><br>D-371: 5 - 5 (0)<br><br>Flexible Lösungen und erschwingliches Equipment.<br><br>D-374: 4 - 4 (0)<br><br>Die Möglichkeit von einem Arzt aufgesucht zu werden, wenn man vielleicht nicht mehr in der Lage ist, selbst eine Praxis aufzusuchen.<br><br>D-398: 4 - 4 (0)<br><br>Aus meinem Bereich wäre es sinnvoll Patienten mit allen technischen Hilfsmitteln wie Behandlungseinheiten auch im Pflegeheim behandeln zu können<br><br>D-398: 4 - 4 (0)<br><br>Parodontale Gesundheit ist das Wichtigste. Auch und für den allgemeinen Gesundheitszustand.

	Specialists	General practitioners	Dentists
	niedrigschwellige fachärztlcihes Versorgungsangebot,<br><br>S-470: 4 - 4 (0)<br><br>soziale Hilfestellungen, Netzwerk, ambulante Hilfen<br><br>S-470: 4 - 4 (0)<br><br>lange Kontakte zu Haus- und Fachärzten, Erreichbarkeit von Apotheken, Krankengymnastik usw.<br><br>S-499: 4 - 4 (0)		Dazu bedarf es eines grossen Umschwungs, besonders in Pflegeeinrichtungen.<br>Es müssen neue Konzepte her, auch baulicher Art (eigener Behandlungsraum ZMK bei Neubau oder Renovierung !)<br><br>D-420: 11 - 13 (0)<br><br>Eine gute Versorgung muss auch ambulant erfolgen, da häufig nicht die Möglichkeit eines Besuchs in der Praxis möglich ist.<br><br>D-433: 4 - 4 (0)<br><br>Rollator/ Rollstuhlgerechte Einrichtung<br><br>D-449: 4 - 4 (0)<br><br>Sicherung der Versorgung bis ins Pflegeheim<br><br>D-451: 9 - 9 (0)<br><br>entsprechend barierefreie Räumlichkeiten<br><br>D-521: 6 - 6 (0)
Ausreichendes und gut geschultes Personal	Kompetenz<br><br>S-216: 6 - 6 (0)	Ausreichend Personal zur Gestaltung eines menschen würdigen Pflege<br><br>GP-219: 4 - 4 (0)<br><br>breite Ausbildung<br><br>GP-310: 4 - 4 (0)	und viel wichtiger geschulte ZFA<br><br>D-305: 3 - 3 (0)<br><br>Aufklärung des Personals und der Angehörigen und Aufnahme der Mundhyigene in den Pflegestandard.<br><br>D-374: 4 - 4 (0)<br><br>Schulungen der Mitarbeiter. Regelmässige Prophylaxe-Intervalle mindesten alle vier Monate. Übernahme der Kosten durch GKV.<br><br>D-420: 14 - 14 (0)<br><br>Die Schulung in den Pflegeheimen sollte vermitteln, dass ein gesundes Gebiß sehr viel Lebensqualität bedeutet. Schmerzfreiheit und gut essen zu können, wirkt sich auch auf die allgemeine Gesundheit und Grundstimmung aus. Weniger Entzündungen schützen auch andere Organsystheme.<br><br>D-427: 4 - 4 (0)<br><br>Fortbildung auch für ZÄ auf dem allgemeinmedizinischen Sektor

	Specialists	General practitioners	Dentists
			D-521: 8 - 8 (0)
Zusammenarbeit mit weiteren Akteur:innen	Ein vertrauensvolle/r Kollege/in.<br><br>S-222: 5 - 5 (0)<br><br>Wichtig ist eine höhere Dichte an Ärzten, v.a. Hausärtzen, und diese sollten auch mehr Zeit haben, gründliche Diagnostiken durchzuführen.<br><br>S-372: 4 - 4 (0)<br><br>Alle Comorbiditäten sollen berücksichtigung findet. Daher ist eine enge Kooperatiion aller Therapeuten wünschenswert.<br><br>S-516: 4 - 4 (0)	Anbindung an mobilen Pflegedienst<br><br>GP-253: 7 - 7 (0)<br><br>besser Vernetzung mit Sozialdienst der Stadt und kirlichen Diensten,die BEsuche übernehmen können, Kontaktadresse (Schüler, die zu hause Fenrseher einstellen können)<br><br>GP-506: 4 - 4 (0)	und viel wichtiger geschulte ZFA<br><br>D-305: 3 - 3 (0)<br><br>Eigene Fitness, gute Betreuung auf allen Ebenen.<br><br>D-396: 4 - 4 (0)<br><br>Insgesamt muss die Pflege mehr Zeit und Geld für die Versorgung alter Menschen haben, damit diese gut versorgt und gesund bleiben.<br><br>D-433: 4 - 4 (0)<br><br>kontrollen und Zahnreinigungen müssten vor Ort 6 mal im Jahr gemacht werden (Pflegepersonal schafft das nicht)<br>Mundhygienezustand ist katastrophal)<br><br>D-479: 5 - 6 (0)<br><br>Weiterführung der Vorsorge auch im Pflegeheim<br><br>D-521: 9 - 9 (0)
Umfeld der Patient:innen	Angehörige miteinbeziehen<br><br>S-231: 4 - 4 (0)<br><br>soziale Hilfestellungen, Netzwerk, ambulante Hilfen<br><br>S-470: 4 - 4 (0)<br><br>gute soziale Einbindung, insbesondere in Familie<br><br>S-499: 4 - 4 (0)	Soziale Anbindung vor allem Unterstützung im Alltag<br><br>GP-244: 7 - 7 (0)<br><br>- Berücksichtigung sozialer Situation/Versorgung<br><br>GP-524: 6 - 6 (0)	Einbindung der Betreuenden<br><br>D-384: 4 - 4 (0)<br><br>Viele dieser Altersgruppe braucht aber dafür Unterstützung, die oft nicht gegeben ist<br><br>D-427: 4 - 4 (0)

	Specialists	General practitioners	Dentists
Komplexität d. Multim. & Polyph., pass. Behandlung	Polymedikation, viele Krankheitsbilder aus unterschiedlichen Gebieten<br><br>S-221: 5 - 5 (0)<br><br>Multipharmazie<br><br>S-231: 5 - 5 (0)<br><br>Multimorbidität. Polymedikation.<br><br>S-232: 5 - 5 (0)<br><br>Fehlendes Patientenverstäbdnis für Situation und Erkrankung.<br><br>S-232: 5 - 5 (0)<br><br>Multiple Medikamente<br><br>S-322: 5 - 5 (0)<br><br>einnahme vieler Medikamente - Frage Nebenwirkung z.B. bei Diagnosen wie Schwindel und Tinnitus<br><br>S-325: 5 - 5 (0)<br><br>Polypharmazie<br><br>S-470: 5 - 5 (0)<br><br>Ein sinnvolles Maß an Diagnostik und Therapie zu finden.<br><br>S-516: 5 - 5 (0)	Wechselwirkungen und Interferenzen verschiedener Medikamente im betagten Organismus.<br><br>GP-201: 5 - 5 (0)<br><br>Geduld, Hilflosigkeit, da man Beschwerden oft nicht verbessern kann, der nahende Tod<br><br>GP-245: 5 - 5 (0)<br><br>Multimorbidität (insbes. kognitive Einschränkungen), Polypharmazie<br><br>GP-353: 5 - 5 (0)<br><br>Multimorbidität, Multimedikation, Multidiagnostik bei Krankenhausaufenthalten<br><br>GP-518: 5 - 5 (0)<br><br>Abnehmende Kognition wird oft überschauspielert, überwachte Medikamenteneinnahme wird abgelehnt// "Das ist was für altete Leute" eine gern gegebene Antwort auf das Angebot einer Gehhilfe//Selbstmedikation (gerne Ibuprofen oder Diclofenac) mit z.B. dramtischen Auswirkungen bei Einnahme NOAK, Niereninsuff. etc., oft echtes "Basteln + Raten" um Ursachen und Wirkungen zu erkennen// u.v.m.<br>GP-518: 5 - 5 (0)<br><br>Besonderheiten bei der Medikation: Leitlinien, die für einzelne Erkrankungen gelten, lassen sich nicht 1:1 umsetzen<br>Abgleich der empfohlenen Medikamente mit der Priscus Liste<br>möglichst nicht mehr als 5 Medikamente!<br>GP-520: 11 - 13 (0)<br><br>Da wir Eingriffe durchführen braucht es mehr Zeit und sehr gute medizinische Kenntniss zum Komplikationen zu vermeiden. Das gelingt in der Praxis meines Erachtens besser als in der Klinik.<br>GP-514: 5 - 5 (0)	Tagesform, Lagerung, Vielzahl der Medikamente die Blutgerinnung behindern, viele Anslgetika ausschließen<br><br>D-362: 5 - 5 (0)<br><br>Multimorbidität<br>Polypharmazie<br><br>D-367: 9 - 10 (0)<br><br>Multimorbidität / Polypharmazie<br><br>D-371: 8 - 8 (0)<br><br>Multimorbidität<br><br>D-374: 5 - 5 (0)<br><br>Medikamentennebenwirkung: Mundtrockenheit.<br><br>D-399: 6 - 6 (0)<br><br>eine optimale Behandlung ist nicht möglich oder sinnvoll wegen anderer Erkrankungen.<br><br>D-422: 5 - 5 (0)<br><br>Multimedikation, besondere Anamneseerhebung.<br><br>D-423: 5 - 5 (0)
Körperl. & kogn. Ein-schärnkungen	kognitive Probleme<br><br>S-163: 5 - 5 (0)	erschwerte Kommunikation<br><br>GP-192: 9 - 9 (0)	-Hör-, Seh- und Tastbermögen des Patienten oft eingeschränkt<br><br>D-189: 16 - 16 (0)

	Specialists	General practitioners	Dentists
beeinflussen Behandl. & Komm.	kognitive defizite<br><br>S-217: 9 - 9 (0)<br><br>mentale Verlangsamung, Schwerhörigkit, Demenz<br><br>S-226: 10 - 10 (0)<br><br>Fehlendes Patientenverstäbdnis für Situation und Erkrankung.<br><br>S-232: 5 - 5 (0)<br><br>Schwerhörigkeit, Immobilität,<br><br>S-470: 5 - 5 (0)	soziale und oder intellektuelle Barrieren bei Therapieumsetzung.<br><br>GP-192: 10 - 10 (0)<br><br>mühsamere Prozesse zur Klärung des Therapieziels<br><br>GP-192: 11 - 11 (0)<br><br>Ältere Patienten brauchen Zeit, sie sind oft äußert umständlich und verlieren den roten Faden im Gespräch<br><br>GP-198: 6 - 6 (0)<br><br>Demente Patienten stellen eine besondere Herausforderung dar, hier kommen oftmals die Angehörigen an ihre Grenzen.<br><br>GP-198: 7 - 7 (0)<br><br>Es kommt eher vor, dass Kommunikationsprobleme aufgrund der Altersschwerhörigkeit vorkommen.<br><br>GP-219: 3 - 3 (0)<br><br>Kommunikation (Vergesslichkeit, Komplexität der Behandlung, was die Pat. nicht verstehen)<br><br>GP-244: 10 - 10 (0)<br><br>Compliancestörung / Demenz<br><br>GP-253: 8 - 8 (0)<br><br>Kommunikation<br><br>GP-275: 7 - 7 (0)<br><br>Kommunikationsprobleme<br><br>GP-508: 5 - 5 (0)<br><br>Kommunikationsbeschränkungen, die sich durch Einschränkung der Sinnesfunktionen ergeben.	-Schwierigkeiten in der Kommunikation (Patienten nehmen weniger auf und können sich nicht mehr so gut mitteilen)<br><br>D-189: 17 - 17 (0)<br><br>Lagerung zur BEhandlung schlecht möglich,<br><br>D-234: 6 - 6 (0)<br><br>man braucht mehr Zeit durch bestehende Beeinträchtigungen (hören, sehen Compliance...)<br><br>D-361: 5 - 5 (0)<br><br>Ergonomie kann man in der regel nicht einhalten (Pat. können nicht so im Beh.-stuhl gekippt werden<br><br>D-361: 6 - 6 (0)<br><br>Tagesform, Lagerung, Vielzahl der Medikamente die Blutgerinnung behindern, viele Anslgetika ausschließen<br><br>D-362: 5 - 5 (0)<br><br>Immobilität<br><br>D-367: 11 - 11 (0)<br><br>Demenzielle Veränderungen,<br><br>D-374: 5 - 5 (0)<br><br>eingeschränkte motorische Fähigkeiten, schwierige Gespräche aufgrund von eingeschränkten kognitiven Möglichkeiten<br><br>D-398: 5 - 5 (0)<br><br>Nachlassende Fähigkeiten bei der eigenen Mundhygiene.<br><br>D-399: 6 - 6 (0)<br><br>Kommunikation<br><br>D-407: 5 - 5 (0)

	Specialists	General practitioners	Dentists
		GP-525: 7 - 7 (0)<br><br>es ist ein deutlich erhöhter Zeitaufwand, Merkfähigkeit ist reduziert<br><br>GP-158: 7 - 8 (0)	Demenz<br><br>D-416: 12 - 12 (0)<br><br>körperliche anstrengende Arbeit, aufgrund physiologischer Defizite (Rollstuhlbehandlungen)<br><br>D-416: 13 - 13 (0)<br><br>Häufigere Pausen während der Behandlung.<br><br>D-423: 5 - 5 (0)<br><br>Dazu kommt die häufige Mobilitätseinschränkung und auch die fortschreitende Demenz.<br><br>D-427: 5 - 5 (0)<br><br>schwierige Kompiance aufgrund körperlicher und geistiger Schwäche<br><br>D-433: 5 - 5 (0)<br><br>Ambulant in der Praxis: Geh- und Sehbehinderungen,<br><br>D-434: 5 - 5 (0)<br><br>Überforderung mit Bürokratie und neuen Medien<br><br>D-434: 5 - 5 (0)<br><br>Erhöhter Auffwand bei der Kommunikation,<br><br>D-449: 5 - 5 (0)<br><br>mehr Zeitaufwand bei eingeschränkter Beweglichkeit<br><br>D-449: 5 - 5 (0)
Fehlende Compliance o. Ablehnung von Unterstützung & Behandlung	sturheit<br><br>S-217: 10 - 10 (0)<br><br>Compliance<br><br>S-231: 5 - 5 (0)	soziale und oder intellektuelle Barrieren bei Therapieumsetzung.<br><br>GP-192: 10 - 10 (0)<br><br>Manche falsche Vorstellung über Krankheit ist nicht mehr auszuräumen	durch verminderte Compliance usw. höherer Zeitaufwand in der Behandlung um ans Ziel zu kommen<br><br>D-383: 6 - 6 (0)<br><br>die Behandlung wird manchmal abgelehnt.

	Specialists	General practitioners	Dentists
		GP-202: 13 - 13 (0)<br><br>Leider viele Patientin möchte auch sich nicht helfen lassen oder lehnen Aufnahme in Altenheim oder betreutes Wohnen kategorisch ab,obwohl die häusliche Versorgung sehr schlecht ist und die Patienten sehr einsam sind.<br><br>GP-244: 12 - 12 (0)<br><br>Compliancestörung / Demenz<br><br>GP-253: 8 - 8 (0)<br><br>Abnehmende Kognition wird oft überschauspielert, überwachte Medikamenteneinnahme wird abgelehnt// "Das ist was für altete Leute" eine gern gegebene Antwort auf das Angebot einer Gehhilfe//Selbstmedikation (gerne Ibuprofen oder Diclofenac) mit z.B. dramtischen Auswirkungen bei Einnahme NOAK, Niereninsuff. etc., oft echtes "Basteln + Raten" um Ursachen und Wirkungen zu erkennen// u.v.m.<br><br>GP-518: 5 - 5 (0)<br><br>-Hilfe Annehmen<br><br>GP-524: 9 - 9 (0)<br><br>- Erkennen/Wahrnehmung und Akzeptanz der Einschränkungen<br><br>GP-524: 10 - 10 (0)	D-422: 5 - 5 (0)<br><br>schwierige Kompiance aufgrund körperlicher und geistiger Schwäche<br><br>D-433: 5 - 5 (0)<br><br>Die Uneinsichtigkeit der Patienten, das sie mit der MH überfordet sind, besonders bei hochwertigem Zahnersatz.<br>Sie lassen sich nicht gerne helfen.<br><br>D-439: 7 - 8 (0)
Umgang mit zeitlichen Ressourcen	Zeitaufwand<br><br>S-231: 5 - 5 (0)<br><br>Zeitmanagement<br><br>S-470: 5 - 5 (0)<br><br>in der Kürze der Zeit das wesentliche für das eigene Fachgebiet zu erkennen und den menschlichen Kontakt nicht zu vergessen<br><br>S-499: 5 - 5 (0)	Ältere Patienten brauchen Zeit, sie sind oft äußert umständlich und verlieren den roten Faden im Gespräch<br><br>GP-198: 6 - 6 (0)<br><br>Zeitmanagement<br><br>GP-275: 6 - 6 (0)<br><br>dauern länger in der Versorgung<br><br>GP-310: 5 - 5 (0)	man braucht mehr Zeit durch bestehende Beeinträchtigungen (hören, sehen Compliance...)<br><br>D-361: 5 - 5 (0)<br><br>durch verminderte Compliance usw. höherer Zeitaufwand in der Behandlung um ans Ziel zu kommen<br><br>D-383: 6 - 6 (0)<br><br>Persönlich zuwenden, entschleunigen, Empathie zeigen, verständliche Sprache verwenden

	Specialists	General practitioners	Dentists
		vieles dauert länger: Schilderung der Beschwerden, körperliche Untersuchung<br><br>GP-520: 10 - 10 (0)<br><br>es ist ein deutlich erhöhter Zeitaufwand, Merkfähigkeit ist reduziert<br><br>GP-158: 7 - 8 (0)<br><br>insb. erhöhter Zeitbedarf<br><br>GP-512: 10 - 10 (0)<br><br>Da wir Eingriffe durchführen braucht es mehr Zeit und sehr gute medizinische Kenntniss zum Komplikationen zu vermeiden. Das gelingt in der Praxis meines Erachtens besser als in der Klinik.<br><br>GP-514: 5 - 5 (0)	D-395: 7 - 7 (0)<br><br>die Ruhe und Kraft sich auf DEREN Geschwindigkeit einzustellen<br><br>D-415: 5 - 5 (0)<br><br>Für mich sind die Besuche im Seniorenpflegeheim die größte Herausforderung.<br>Mein gesamtes Praxisteam ist in die Logistik und den Transport der mobilen Behandlungseinheit und der Materialien eingebunden. Im Pflegeheim fehlt dann oft ein kompetenter Ansprechpartner, so dass die zahnärztliche Behandlung dort sehr zeitintensiv ist.<br><br>D-447: 7 - 8 (0)<br><br>mehr Zeitaufwand bei eingeschränkter Beweglichkeit<br><br>D-449: 5 - 5 (0)<br><br>es bedarf stets der Empathie, Geduld und Aufrichtigkeit bei den Behandlungen.<br><br>D-451: 10 - 10 (0)
Angehörige und weitere Stakeholder/ Akteure	Die Einbeziehung von Kontaktpersonen & Mitentscheidern (wie Betreuer und Angehörigen) ist oft problematisch und fustrierend.<br><br>S-516: 5 - 5 (0)	Kontakt zu Betreungspersonen ist nicht immer einfach z.B. Angehörige, Pflegende, Betreuer<br><br>GP-202: 12 - 12 (0)<br><br>Hohe Anspruchshaltung der Zugehörigen, die entweder die deutlich bescheideneren Ziele ihrer alten Verwandten/Freunde nicht akzeptieren können/wollen; oft unbearbeitete Konflikte, die in Überfürsorglichkeit münden, die den Möglichekiten des /der Betroffenen nicht gerecht werden.<br><br>GP-525: 6 - 6 (0)	Behördenwahnsinn und DSGVO wenn Betreuer etc. ins Spiel kommt.<br>D-305: 4 - 4 (0)<br><br>mangeldenes Wissen über Mundhygiene bei Pflegepersonal und ANgehörigen.<br>D-374: 5 - 5 (0)<br><br>und mangelndes Kooperationsverhalten der Betreuer oder Angehörigen.<br>D-374: 6 - 6 (0)<br><br>Zusammenarbeit mit HA,<br>D-407: 5 - 5 (0)<br><br>ständige Absprache mit Vormundschaft<br>D-416: 14 - 14 (0)<br><br>Fehlende Einwilligung zur Behandlung der Betreuer.

	Specialists	General practitioners	Dentists
			D-422: 5 - 5 (0)<br><br>Wiederstand der Pflegeeinrichtungen, unsere Arbeit zu unterstützen. Eine Fortbildung ist dann nachgefragt,wenn der MdK sich ankündigt. Was man noch alles vom Pflegepersonal verlangen will.<br>D-427: 5 - 5 (0)<br><br>Die Betreuung durch Angehörige ist oft schwieriger als mit gesetzlichen Betreuern.<br>D-427: 5 - 5 (0)<br><br>In den Heimen liegt der Schwerpunkt meistens NICHT bei der Mundhygiene. Das Pflegepersonal ist dahingegen nicht gut geschult.<br>D-439: 9 - 9 (0)<br><br>Für mich sind die Besuche im Seniorenpflegeheim die größte Herausforderung.<br>Mein gesamtes Praxisteam ist in die Logistik und den Transport der mobilen Behandlungseinheit und der Materialien eingebunden. Im Pflegeheim fehlt dann oft ein kompetenter Ansprechpartner, so dass die zahnärztliche Behandlung dort sehr zeitintensiv ist.<br>D-447: 7 - 8 (0)<br>Fehlendes Engagement der Angehörigen<br>D-454: 7 - 7 (0)<br>Abklärung des Betreuungstsatus, Genehmigungen einholen<br>D-521: 10 - 10 (0)
Behandlungs-ziele & (nicht-med.) Anliegen der Patient:innen	Vermeidung von Maximalismus unter berücksichtigung der begrenzten Lebenserwartung<br><br>S-175: 6 - 6 (0)<br><br>Medizin in der letzten Lebnsphase kann herausforern, aber auch sehr erfüllend sein<br><br>S-196: 9 - 9 (0)<br><br>THema "Endlichkeit" wird oft von Hasuärzten ausgebelndet und dem Patineten sugeredet<br><br>S-196: 10 - 10 (0)<br><br>Ein sinnvolles Maß an Diagnostik und Therapie zu finden.	Einsamkeit ist ein großes Problem, weshalb einige Patienten täglich vorbeikommen, um sich jedes Medikament einzeln aufschreiben zu lassen und einen kleinen Plausch mit den Mitarbeiterinnen zu führen. In Coronazeiten manchmal ein Problem.<br><br>GP-198: 6 - 6 (0)<br><br>Manche Patienten besuchen Praxen bzw. Krankenhäuser häufiger, die medizinisches Personal fast die einzige soziale Kontakt !<br><br>GP-244: 13 - 13 (0)<br><br>Geduld, Hilflosigkeit, da man Beschwerden oft nicht verbessern kann, der nahende Tod<br><br>GP-245: 5 - 5 (0)	Altersarmut<br><br>D-367: 12 - 12 (0)<br><br>altengerechte Zahnmedizin<br><br>D-396: 5 - 5 (0)<br><br>es bedarf stets der Empathie, Geduld und Aufrichtigkeit bei den Behandlungen.<br><br>D-451: 10 - 10 (0)

	Specialists	General practitioners	Dentists
	S-516: 5 - 5 (0)	Die Vereinsamung nach Verlust der Partner oder Freunden<br><br>GP-271: 5 - 5 (0)<br><br>angetragene Anforderung, welche nicht originär zur medizinischen Versorgung gehören<br><br>GP-508: 6 - 6 (0)<br><br>Hohe Anspruchshaltung der Zugehörigen, die entweder die deutlich bescheideneren Ziele ihrer alten Verwandten/Freunde nicht akzeptieren können/wollen; oft unbearbeitete Konflikte, die in Überfürsorglichkeit münden, die den Möglichekiten des /der Betroffenen nicht gerecht werden.<br><br>GP-525: 6 - 6 (0)<br><br>siehe die ersten beiden Punkte. die meiste Zeit bindet es Transporte in die Praxis genehmigen zu lassen, Pfegedienste zu kontaktieren und diese ganze häusliche Versorgung zu verbessern, das Medizinsiche ist m.E. das geringste Problem<br>GP-506: 5 - 5 (0)	
Strukturelle und organisatorische Heraus-forderungen	Bereitstellen einer Gesundheitsakte mit allen Informationen des Patienten wie Vorerkrankungen, Vor-OPs, Medikamente und Befunde von Untersuchungen<br><br>lückenhafte Anamnese und fehlende Befunde<br><br>S-169: 4 - 5 (0)<br><br>nachlassende Mobilität hält von Aktivitöäten ab, im ländlichen Raum werden die Ressourcen und Angboete zunehmend knapper<br><br>S-196: 8 - 8 (0)<br><br>Ambulante Versorgung vor Ort<br><br>S-216: 7 - 7 (0)<br><br>Mangelnde instrumentelle Ausstattung<br><br>S-469: 5 - 5 (0)	all das , was ich auf der Seite vorher beschrieben habe zu gewährleisten<br><br>GP-202: 11 - 11 (0)<br><br>Verbesserung der häuslichen Versorgung<br><br>GP-244: 11 - 11 (0)<br><br>stationäre Versorgung oft fragwürdig ("früher" waren die Patienten nach Entlassung erst mal gut und stabil, heute fahren wir oft notfallmäßig sofort nach Entlassung einen Hausbesuch, um die verbliebenen Braunülen, EKG-Elektroden und Dauerkatheter zu entfernen; das nervt)<br><br>GP-518: 5 - 5 (0)<br><br>Fehlende/unzureichende Versorgungsstrukturen, wenn die finanziellen Mittel des alten Menschen aufgebraucht sind.<br><br>GP-525: 8 - 8 (0)	administrativer Mehraufwand sehr erheblich,<br>D-234: 5 - 5 (0)<br><br>Behördenwahnsinn und DSGVO wenn Betreuer etc. ins Spiel kommt.<br>D-305: 4 - 4 (0)<br><br>Altersarmut<br>D-367: 12 - 12 (0)<br><br>Schwierigkeiten bei der Absprache mit anderen Fachdisziplinen durch schlechte Erreichbarkeit<br>D-371: 6 - 6 (0)<br><br>Sammeln aller wichtigen Informationen (medizinische Daten, Kontaktdaten Angehörige/ Betreuer/...)<br>D-371: 7 - 7 (0)<br><br>Mangelnde und nur sehr schwere Absprache mit HA zu Medikation, häufig demenzielle Veränderungen machen Aufklärung schwer.<br>D-374: 3 - 3 (0)

	Specialists	General practitioners	Dentists
		siehe die ersten beiden Punkte. die meiste Zeit bindet es Transporte in die Praxis genehmigen zu lassen, Pfegedienste zu kontaktieren und diese ganze häusliche Versorgung zu verbessern, das Medizinsiche ist m.E. das geringste Problem<br><br>GP-506: 5 - 5 (0)	unzureichendes und sehr teures Equipiment. Teilweise sehr schweres und unhandliches Equipment.<br>D-374: 6 - 6 (0)<br><br>Bürokratisierung der Untersuchung<br>D-374: 6 - 6 (0)<br><br>Terminplanung<br>D-407: 5 - 5 (0)<br><br>Transport<br>D-407: 5 - 5 (0)<br><br>häufig Aufsuchende Tätigkeit (Besuche)<br>D-436: 5 - 5 (0)<br><br>Für mich sind die Besuche im Seniorenpflegeheim die größte Herausforderung. Mein gesamtes Praxisteam ist in die Logistik und den Transport der mobilen Behandlungseinheit und der Materialien eingebunden. Im Pflegeheim fehlt dann oft ein kompetenter Ansprechpartner, so dass die zahnärztliche Behandlung dort sehr zeitintensiv ist.<br>D-447: 7 - 8 (0)<br><br>Corona, man kommt nicht ins Heim hinein<br>D-479: 7 - 7 (0)
Keine Heraus-forderungen	keine. Ich arbeite mittlerweile lieber mit den Älteren Patienten als mit den Jüngeren!<br><br>S-222: 8 - 8 (0)		keine<br><br>D-432: 5 - 5 (0)
(Gesundheitl.) Zustand und Fähigkeiten der Patient:innen	Multi morbid<br><br>S-216: 9 - 9 (0)<br><br>an (z.B. cardialer/anästhesiolog.) Nichtoperabilität,<br><br>S-226: 12 - 12 (0)<br><br>an Interaktionen (additive Effelte) der vorhandenen Medikation mit meiner gewünschten Verordnung<br><br>S-226: 12 - 12 (0)	Behinderung durch sprachliche, private und soziale Barrieren<br><br>GP-192: 14 - 14 (0)<br><br>Grenzen der Polypharmazie, UAW der Medikation<br><br>GP-192: 15 - 15 (0)<br><br>Komplexität der Erkrankung/ Behandlung<br><br>GP-244: 15 - 15 (0)<br><br>kognitive Einschränkungen	Patient will immer nur das Nörigste, dann kommen die Probleme, wenn der Allgemeinzustand sinnvolle Therapie nicht mehr zulässt<br><br>D-362: 7 - 7 (0)<br><br>unsichere / eingeschränkte Allgemeingesundheit<br><br>D-371: 10 - 10 (0)<br><br>Patient kann nicht mehr so einfach / leicht in die Praxis kommen<br><br>D-371: 10 - 10 (0)<br><br>immer wieder kehrende mangelnde Mundhygiene.

Specialists	General practitioners	Dentists
	GP-353: 7 - 7 (0)<br><br>siehe vorne, Medizinisch sind meine Patienten gut versorgt, aber oft fehlt der Besuchsdienst gegen Einsamkeit, das Essen auf Rädern, Hilfe bei VErschelchterung der körperlichen Situation, wer beantragt Pflegestufe etc, das ist total schwierig für die Meisten<br><br>GP-506: 7 - 7 (0)<br><br>fehlende Mobilität<br><br>GP-512: 13 - 13 (0)	D-374: 8 - 8 (0)<br><br>Schlechte oder unmögliche Lagerung.<br><br>D-374: 8 - 8 (0)<br><br>Geringe Belastbarkeit<br><br>D-376: 6 - 6 (0)<br><br>gesundheitliche Situation der Pat.<br><br>D-383: 8 - 8 (0)<br><br>an der geistigen und der körperlichen Fitness<br><br>D-384: 7 - 7 (0)<br><br>teilweise Altenheimpatienten, daher immobil<br><br>D-397: 7 - 7 (0)<br><br>Alterserscheinungen wie Knochenabbau machen Therapien zum Teil unmöglich (Prothese ohne Knochen hält einfach nicht, egal was man auch versucht)<br><br>D-398: 7 - 7 (0)<br><br>Eingeschränkte Belastbarkeit.<br><br>D-399: 8 - 8 (0)<br><br>Immobilität der Patienten: Behandlung in der Praxis nicht möglich.<br><br>D-399: 8 - 8 (0)<br><br>Kompromiss Behandlung wegen des Gesundheitszustandes, der Räumlichkeiten (Altenheim).<br>Wunsch des Pat. nach weniger aufwendiger Behandlung<br><br>D-411: 10 - 11 (0)<br><br>wirtschaftliche Verhältnisse/Altersarmut/gesundheitliche Gründe<br><br>D-415: 7 - 7 (0)

Specialists	General practitioners	Dentists
		Gebrechlichkeit<br><br>D-420: 17 - 17 (0)<br><br>Schlechte Erfahrungen durch Vorbehandler.<br>Kaum oder geringe Zahnarzterfahrung.<br>Sprachbarrieren.<br>und weitere mehr...<br><br>D-420: 18 - 21 (0)<br><br>am geistigen und körperlichen Gesundheitszustand.<br><br>D-422: 7 - 7 (0)<br><br>Schwierig ist häufig die Kommunikation mit den Demenzpatienten.<br><br>D-427: 7 - 7 (0)<br><br>in ester Linie am Gesundheitszustand<br><br>D-435: 7 - 7 (0)<br><br>an der Mobilität der Pat<br><br>D-436: 7 - 7 (0)<br><br>Mangelnde Kooperation , meist durch starke Demenz.<br><br>D-439: 11 - 11 (0)<br><br>Das, was im Zahnmedizinstudium gelehrt wird, wird so in der Praxis oft relativiert. Mir ist wichtig, dass die Patienten schmerzfrei sind, essen können, mit ihrer Mundsituation zufrieden sind und der Verfall der Zähne möglichst verlangsamt wird.<br>Wenn aber Patienten bettlägerig werden, Vollpflegefälle werden, ganz auf die Hilfe von Pflegepersonal angewiesen sind, ist eine zahnmedizinische Therapie kaum mehr möglich.<br>D-447: 11 - 12 (0)<br><br>eingeschränkte Fingerfertigkeit der Patienten<br><br>D-449: 7 - 7 (0)

	Specialists	General practitioners	Dentists
			eingeschränkte Mobilität schränkt umfangreichere prothetische Möglichkeiten ein.<br>D-451: 15 - 15 (0)<br><br>trotz mobiler Dentalinheit ist nicht alles möglich Gesundheitszustand der Patienten<br>D-451: 16 - 17 (0)<br><br>Patienten können wegen Alterseinschränkungen nicht so gelagert werden, wi es sein müsste<br>D-479: 9 - 9 (0)<br><br>eingeschränkte AZ, z.B. keine Abdrücke mögich<br>D-521: 12 - 12 (0)
Umfeld der Pat./weitere Stakeholder & Akteure	Am Einsichtsverhalten der Patienten aber oft auch an ihrem persönlichen Umfeld<br><br>S-175: 8 - 8 (0)<br><br>keine Begleitperson<br><br>S-499: 7 - 7 (0)	Akzeptanz der Patienten, Akzeptanz der Angehörigen,<br><br>GP-192: 13 - 13 (0)<br><br>Behinderung durch sprachliche, private und soziale Barrieren<br><br>GP-192: 14 - 14 (0)<br><br>An der Compliance sowohl der alten Patienten als auch deren jüngeren Angehörigen<br><br>GP-219: 7 - 7 (0)<br><br>Einsamkeit und schlechte häusliche Versorgung<br><br>GP-244: 16 - 16 (0)<br><br>zu hohes und kontraproduktives Anspruchsdenken der Patienten oder/und der Angehörigen<br><br>GP-245: 7 - 7 (0)<br><br>Angehörige der Patienten und ihre Forderungen<br>GP-508: 8 - 8 (0)<br><br>instabiles Umfeld<br>GP-508: 9 - 9 (0)<br><br>mangelndes Engagement der Angehörigen<br>GP-518: 9 - 9 (0)	oder Betreuer kümmern sich nicht, oder haben andere Interessen als der Betreute.<br><br>D-234: 8 - 8 (0)<br><br>mangelnde Kooperation bei Angehörigen,<br><br>D-371: 10 - 10 (0)<br><br>keine Einwillung des Betreuers<br><br>D-374: 8 - 8 (0)<br><br>keine Mitarbeit im Heim / Familie;<br><br>D-384: 7 - 7 (0)<br><br>Fehlende Unterstützung durch Dritte.<br><br>D-420: 17 - 17 (0)<br><br>Betreuung, Sozialhilfe,<br><br>D-451: 14 - 14 (0)<br><br>An der fehlenden Bereitschaft der Angehörigen sich zu kümmern und Geld in die Gesundheit zu investieren<br><br>D-454: 9 - 9 (0)

	Specialists	General practitioners	Dentists
		Umsetzung der notwendigen Schritte im häuslichen Umfeld schwierig<br>GP-158: 10 - 10 (0)<br><br>fehlende häusliche Betreuung, die muss gegeben sein. Z.n. nach einer Sedierung<br>GP-514: 7 - 7 (0)<br><br>fehlende Unterstützung durch das Umfeld<br>GP-524: 13 - 13 (0)	
Fehlende Compliance oder Einsicht	Am Einsichtsverhalten der Patienten aber oft auch an ihrem persönlichen Umfeld<br><br>S-175: 8 - 8 (0)<br><br>Compliance<br><br>S-231: 7 - 7 (0)<br><br>Fehlende Einsicht, fehlendes Verständnis, fehlende Compliance<br><br>S-232: 7 - 7 (0)	Akzeptanz der Patienten, Akzeptanz der Angehörigen,<br>GP-192: 13 - 13 (0)<br><br>Fehlende Akzeptanz und fehlende Einsicht<br>GP-201: 7 - 7 (0)<br><br>fehlende Compliance<br>GP-202: 15 - 15 (0)<br><br>An der Compliance sowohl der alten Patienten als auch deren jüngeren Angehörigen<br>GP-219: 7 - 7 (0)<br><br>Therapieeinahme kann nicht 100% kontrollieret werden<br>GP-244: 17 - 17 (0)<br><br>Teilweise Ablehnung mancher Patienten der angebotenen bzw. vorgeschalgenen Lösungen/Hilfe<br>GP-244: 18 - 18 (0)<br><br>zu hohes und kontraproduktives Anspruchsdenken der Patienten oder/und der Angehörigen<br>GP-245: 7 - 7 (0)<br><br>Zu wenig Zeit<br>GP-253: 10 - 10 (0)<br><br>"Altersstarrsinn"<br><br>GP-518: 8 - 8 (0)<br><br>fehlende Compliance<br><br>GP-520: 17 - 17 (0)	Pateint möchte die Ergebnisse seiner Zahnbefundung nicht mehr umsetzen<br><br>D-234: 8 - 8 (0)<br><br>Patient will immer nur das Nörigste, dann kommen die Probleme, wenn der Allgemeinzustand sinnvolle Therapie nicht mehr zulässt<br><br>D-362: 7 - 7 (0)<br><br>Wunsch des Patienten<br><br>D-367: 14 - 14 (0)<br><br>Folgetermine / Vorgsorgetermine werden nicht regelmäßig wahrgenommen,<br><br>D-371: 10 - 10 (0)<br><br>keine Compliance<br><br>D-384: 7 - 7 (0)<br><br>Kompromiss Behandlung wegen des Gesundheitszustandes, der Räumlichkeiten (Altenheim).<br>Wunsch des Pat. nach weniger aufwendiger Behandlung<br><br>D-411: 10 - 11 (0)<br><br>Einsicht<br><br>D-420: 17 - 17 (0)<br><br>Mangelnde Kooperation , meist durch starke Demenz.<br><br>D-439: 11 - 11 (0)

	Specialists	General practitioners	Dentists
		mangelnde Einsicht bei Patienten, Störrigkeit<br><br>GP-512: 12 - 12 (0)<br><br>- Adhärenz<br><br>GP-524: 12 - 12 (0)	Oft ist das, was zahnmedizinisch sinnvoll ist, nicht das, was dem Patientenwunsch entspricht.<br><br>D-447: 10 - 10 (0)
Finanzielle Gründe		Zeit und angepasste Geldmittel<br><br>GP-275: 9 - 9 (0)<br><br>Ablehnung von Verordnungen durch die KK<br><br>GP-518: 7 - 7 (0)	-Ausreichend Geldmittel für die meist sehr armen, älteren Menschen durch soziale Systeme besonders für den unterfinanzierten Bereich der Zahnmedizin, seit der Einführung des Festzuschusssystems (Stichwort: Patient kann sich trotz einer Krankenversicherung keine Basisversorgung (zB Prothese) leisten, weil der Eigenanteil bei kleiner Rente zu hoch ist, man aber trotzdem noch zu viel Einkommem hat um durch die sozialen Systeme die volle Kostenerstattung zu erhalten.)<br><br>D-189: 13 - 13 (0)<br><br>Kosten und Behhördenwahnsinn<br><br>D-305: 6 - 6 (0)<br><br>finanzielle Probleme<br><br>D-361: 8 - 8 (0)<br><br>Kostenübernahme fraglich,<br><br>D-371: 10 - 10 (0)<br><br>finanzielle Situation,<br><br>D-383: 8 - 8 (0)<br><br>Krankenkassenleistung<br><br>D-396: 7 - 7 (0)<br><br>Eingeschränkte finanzielle Möglichkeiten.<br><br>D-399: 8 - 8 (0)<br><br>wirtschaftliche Verhältnisse/Altersarmut/gesundheitliche Gründe<br><br>D-415: 7 - 7 (0)

Specialists	General practitioners	Dentists
		Betreuer/ Vormund aufgrund finanziellen Situation<br><br>D-416: 16 - 16 (0)<br><br>Geld<br><br>D-420: 17 - 17 (0)<br><br>Eingeschränkte Behandlungsmethoden in ambulanter Tätigkeit als Zahnarzt bei der aufsuchenden Betreuung. Teils bei der Kostenbeteiligung. Es wird direkt gesagt, wenn eigene Kosten entstehen,wünschen wir keine Behandlung, ohne sich die Möglichkeiten und den Nutzen für den Patienten aufzeigen zu lassen .<br><br>D-427: 7 - 7 (0)<br><br>Finanzielle Gründe<br><br>D-434: 7 - 7 (0)<br><br>zu hoher Kostenaufwand bei Zahnersatz<br><br>D-449: 7 - 7 (0)<br><br>ich habe dieses Jahr ca 750 Hausbesuche gemacht.<br>Die Möglichkeiten sind bei diesen Patienten begrenz durch:<br>finanzielle Möglichkeiten<br><br>D-451: 12 - 14 (0)<br><br>Betreuung, Sozialhilfe,<br><br>D-451: 14 - 14 (0)<br><br>An der fehlenden Bereitschaft der Angehörigen sich zu kümmern und Geld in die Gesundheit zu investieren<br><br>D-454: 9 - 9 (0)<br><br>im Pflegeheim mangelnde Kooperation von Berufsbetreuern, Geld spielt eine Rolle<br><br>D-521: 13 - 13 (0)

	Specialists	General practitioners	Dentists
			eingeschränkte finanzielle Möglichkeiten<br><br>D-521: 14 - 14 (0)
Zeitaufwand/-mangel	Zeitprobleme<br><br>S-163: 7 - 7 (0)<br><br>Zeit, fehlende Befunde anderer Fachgebiete<br><br>S-221: 7 - 7 (0)<br><br>In meiner Praxis werden alle Altersgruppen behandelt. Alle brauchen Zeit und Zuwendung.<br><br>S-223: 8 - 8 (0)<br><br>zu großer Zeitaufwand gemessen an der Vergütung<br><br>S-231: 7 - 7 (0)<br><br>Zeitmangel<br><br>S-325: 7 - 7 (0)<br><br>Zeitfaktor<br><br>S-470: 7 - 7 (0)<br><br>Zeitmangel<br><br>S-499: 7 - 7 (0)	Zeitmangel<br><br>GP-198: 9 - 9 (0)<br><br>Wir haben unsere Arbeitszeiten stark reduziert und damit weniger Zeit<br><br>GP-271: 7 - 7 (0)<br><br>Zeit und angepasste Geldmittel<br><br>GP-275: 9 - 9 (0)<br><br>Ich habe einen für eine Einzelpraxis viel zu großen Anteil an Hausbesuchspatienten - und siehe bitte auch Antwort zur vorletzten Frage.<br><br>GP-525: 10 - 10 (0)	Zeit<br><br>D-189: 14 - 14 (0)<br><br>Nicht ausreichend Zeit<br><br>D-374: 8 - 8 (0)<br><br>Zeit<br><br>D-396: 7 - 7 (0)

	Specialists	General practitioners	Dentists
(Fehlender) Info-austausch & Zusammen-arbeit m. weiteren Akteuren	Zeit, fehlende Befunde anderer Fachgebiete<br><br>S-221: 7 - 7 (0)<br><br>Kommunikation mit Patient und anderen Ärzten,<br><br>S-231: 7 - 7 (0)<br><br>fehlende Unterlagen,<br><br>S-499: 7 - 7 (0)<br><br>An fehlenden Informationen und mangelnder Kooperation mit anderen Therapeuten<br><br>S-516: 7 - 7 (0)		fehlende Kommunikation<br><br>D-395: 9 - 9 (0)<br><br>Kooperation Pflegeeinrichtungen<br><br>D-396: 7 - 7 (0)<br><br>Ablehnung ohne die Wünsche der Patienten zu berücksichtigen. (Behandlungsansätze stimmen wir gerne mit den Hausärzten ab, die gerade in den Pflegeheimen die Gesundheit der Bewohner mit all ihren Beschwerden, besser kennen).<br><br>D-427: 7 - 7 (0)<br><br>Der überwiegende Teil der von uns betreuten Patienten leben in Pflegeheimen und werden durch das Pflegepersonal häufig nicht zur Mundhygiene angehalten.<br><br>D-439: 12 - 12 (0)<br><br>im Pflegeheim mangelnde Kooperation von Berufsbetreuern, Geld spielt eine Rolle<br><br>D-521: 13 - 13 (0)
Mangel an (qualifiziertem) Personal	An Personalmangel<br><br>S-372: 7 - 7 (0)<br><br>Personalmangel im Altenheim<br><br>S-469: 7 - 7 (0)	fehlende Ressourcen im System (z.B. Physio- Ergo- Psychotherapie)<br><br>GP-202: 16 - 16 (0)<br><br>Überforderung der Pflegedienstmitarbeiter<br><br>GP-518: 10 - 10 (0)<br><br>fehlende Facharztversorgung<br><br>GP-520: 15 - 15 (0)<br><br>fehlende Psychotherapieplätze<br><br>GP-520: 16 - 16 (0)	personelle Engpässe,<br><br>D-395: 9 - 9 (0)
Bürokratie	Bürokratie in Pflegeheimen<br><br>S-499: 7 - 7 (0)	siehe vorne, Medizinisch sind meine Patienten gut versorgt, aber oft fehlt der Besuchsdienst gegen Einsamkeit, das Essen auf Rädern, Hilfe bei VErschelchterung der	Kosten und Behhördenwahnsinn<br><br>D-305: 6 - 6 (0)

	Specialists	General practitioners	Dentists
		körperlichen Situation, wer beantragt Pflegestufe etc, das ist total schwierig für die Meisten<br><br>GP-506: 7 - 7 (0)	
(Infra-)Struktur der Versorgung	Unterversorgung an altersgerechten Transport- und Versorgungssytemen.<br><br>S-175: 8 - 8 (0)<br><br>speziell in meine Fachbereich: an den nicht verfügbaren Psychotherapaieplätzen<br><br>S-196: 12 - 12 (0)<br><br>Ambulant nicht machbar<br><br>S-216: 10 - 10 (0)<br><br>AM SYSTEM<br><br>S-217: 12 - 12 (0)<br><br>Gesellschaft und Krankenkassen machen sich zunehmend keine Gedanken über eine auskömmliche Finanzierung (z. B. Honorare) in der Versorgung.<br>Als Ärztin und Arzt wird man zunehmend nur ausgequetscht und ausgenutzt vom "Gesundheitswesen" (Politik, GKV, PKV).<br><br>S-223: 9 - 10 (0)	GKV<br><br>GP-310: 7 - 7 (0)<br><br>keine individuellen Gestaltungsmöglichkeiten (mehr)<br><br>GP-518: 11 - 11 (0)<br><br>Ich habe einen für eine Einzelpraxis viel zu großen Anteil an Hausbesuchspatienten - und siehe bitte auch Antwort zur vorletzten Frage.<br><br>GP-525: 10 - 10 (0)	nicht transportables Equipment,<br><br>D-374: 8 - 8 (0)<br><br>Pflegeheimeinrichtungen, Gesundheitssytem ,<br><br>D-382: 7 - 7 (0)<br><br>An das Versorgungs- und Pflegesystem,<br><br>D-395: 9 - 9 (0)<br><br>fehlende Behandlungsmöglichkeit in Pflegeeinrichtung,<br><br>D-398: 7 - 7 (0)<br><br>Kompromiss Behandlung wegen des Gesundheitszustandes, der Räumlichkeiten (Altenheim).<br>Wunsch des Pat. nach weniger aufwendiger Behandlung<br><br>D-411: 10 - 11 (0)<br><br>Eingeschränkte Behandlungsmethoden in ambulanter Tätigkeit als Zahnarzt bei der aufsuchenden Betreuung. Teils bei der Kostenbeteiligung. Es wird direkt gesagt, wenn eigene Kosten entstehen,wünschen wir keine Behandlung, ohne sich die Möglichkeiten und den Nutzen für den Patienten aufzeigen zu lassen .<br><br>D-427: 7 - 7 (0)<br><br>trotz mobiler Dentalinheit ist nicht alles möglich<br>Gesundheitszustand der Patienten<br><br>D-451: 16 - 17 (0)
keine förderlichen Faktoren			Nichts<br><br>D-305: 7 - 7 (0)
Erfahrung der Behandelnden	persönliche Erfahrung und allgemeine Studienlage	Erfahrung	Erfahrunh

	Specialists	General practitioners	Dentists
	S-169: 8 - 8 (0)<br><br>Mein langjährige Erfahrung<br><br>S-516: 8 - 8 (0)	GP-518: 14 - 14 (0)<br><br>meien Erfahrung<br><br>GP-506: 8 - 8 (0)<br><br>persönliche Erfahrung<br><br>GP-512: 14 - 14 (0)	D-362: 8 - 8 (0)<br><br>mein eigenes Alter zusammen mit meiner Berufserfahrung als Hauszahnarzt<br><br>D-384: 8 - 8 (0)<br><br>Meine lange Erfahrung hilft mir.<br><br>D-420: 23 - 23 (0)
Fortbildungen und Qualifikation der Behandelnden	persönliche Erfahrung und allgemeine Studienlage<br><br>S-169: 8 - 8 (0)<br><br>Die eigene Ausbildung und eigene Familie<br><br>S-322: 8 - 8 (0)<br><br>ausreichendes und qualifiziertes Personal<br><br>S-372: 8 - 8 (0)<br><br>Genug Personal gelegentlich<br><br>S-469: 8 - 8 (0)	Vorhandensein von Pflegepersonal mit guter Ausbildung , aber auch die Zahl des Pflegepersonal soll auch stimmen. In manchen Pflegeheimen habe ich erlebt, dass nur eine Schwester für mind 30 Patienten zuständig ist, was überhaupt nicht möglich ist !!!<br><br>GP-244: 22 - 22 (0)<br><br>In meinen Kooperationsheimen der hervorragende Teamgeist und die hohen Qualitätsniveaus; innerhalb der Praxis die Arbeitsphilosphie meines Teams.<br><br>GP-525: 11 - 11 (0)<br><br>Gut ausgebildete Mitarbeiter<br><br>GP-514: 8 - 8 (0)<br><br>und meine Fortbildungen über viele Jahres<br><br>GP-514: 8 - 8 (0)	Fortbildungen seitens der ZÄKWL, Internetrecherche<br><br>D-234: 9 - 9 (0)<br><br>regelmäßige Fortbildungen in diesem Bereich<br><br>D-371: 11 - 11 (0)<br><br>? Die Frage sollt sein wer! Politisch muss sich etwas tun, mehr Ärzte sollten dazu bereit sein Patienten zu versorgen. Entbürokratisierung ist wichtig. Genau so die Publizieren von Hygienemaßnahmen und der Möglichekit der Hausbesuche.-<br><br>D-374: 9 - 9 (0)<br><br>Persöhnliche Fortbildung<br><br>D-396: 8 - 8 (0)<br><br>improvisationstalent. Leider muss man sich aber eingestehen, dass man nicht alle zur vollsten Zufriedenheit versorgen kann<br><br>D-398: 8 - 8 (0)<br><br>gut ausgebildetes personal in meiner Praxis<br><br>D-521: 17 - 17 (0)
Haltung und Motivation der Behandelnden	mein Idealismus, meine Kreativiät<br><br>S-196: 13 - 13 (0)<br><br>selbstausbeutung<br><br>S-217: 13 - 13 (0)	Mein Team aus MFA<br><br>GP-192: 16 - 16 (0)<br><br>Ich behandele meine älteren Patienten so, wie ich mir es für meinen alten Eltern wünsche.<br><br>GP-198: 10 - 10 (0)	-Das Vertrauen, darin, dass der Patient und der Behandler am besten wissen, was richtig und gut für den Patienten ist. und dass manchmal die Empfehlungen der Fachgesellschaften nicht für jedes Individuum ideal sind und man dann auch davon abweichen darf und sollte<br>(Beispiel: Nach sachlichen Kriterien ist die Prothese des Patienten absolut unpassen, die Zähne müssten erneuert werden und sie sieht zusätzlich auch noch grauenvoll aus. Der Patient kommt aber wunderbar zurecht, kann sogar die härtesten Möhren damit knacken und ist absolut zufrieden.

Specialists	General practitioners	Dentists
Praxisteam, Angehörige, Pflegende<br><br>S-221: 8 - 8 (0)	meine Mitarbeiter<br><br>GP-245: 8 - 8 (0)	Würde man ihm jetzt eine neue Prothese erstellen nach fachlich korrekten Vorgaben, dann wäre es sehr gut möglich, dass der Patient sich damit nicht anfreunden kann, nichts mehr kauen kann und sehr unglücklich wird, weil seine Anpassungsfähigkeit im Alter einfach nicht mehr so gegeben ist, wie in jungen Jahren.<br>Also lässt man den Patienten glücklich und zufrieden mit seiner alten Prothese weiter kauen.<br><br>D-189: 20 - 23 (0)
meine Einstellung<br><br>S-222: 11 - 11 (0)	Innerer Antrieb/ Ehrgeiz,Empathie<br><br>GP-253: 11 - 11 (0)	emphatische Mitarbeiter<br><br>D-362: 8 - 8 (0)
Niemand. Doch, meine Mitarbeiterinnen soweit es möglich ist.<br><br>S-223: 11 - 11 (0)	Mitarbeiter<br><br>GP-275: 11 - 11 (0)	Ethischer Kompass<br><br>D-367: 15 - 15 (0)
Die eigene Ausbildung und eigene Familie<br><br>S-322: 8 - 8 (0)	Praxisteam und externe Mitversorger, z.B. Pat.-Familie/Pflegedienst/Heimpersonal<br><br>GP-353: 8 - 8 (0)	meine Einstelluing zum Beruf;<br><br>D-384: 8 - 8 (0)
gutes Praxisteam<br><br>S-325: 8 - 8 (0)	Empathie<br><br>GP-518: 12 - 12 (0)	ein eingespieltes Team;<br><br>D-384: 8 - 8 (0)
persönlcihes Engagement,<br><br>S-470: 8 - 8 (0)	Aushalten (z.B. bei drohenden Regressen)<br><br>GP-518: 13 - 13 (0)	Mitarbeit meiner Angestellten.<br><br>D-399: 9 - 9 (0)
Mitarbeiter<br><br>S-470: 8 - 8 (0)	gutes Praxisteam<br><br>GP-518: 15 - 15 (0)	Mein persönlicher Einsatz<br><br>D-415: 8 - 8 (0)
Pflegepersonal in stat. Einrichtungen.<br><br>S-516: 8 - 8 (0)	In meinen Kooperationsheimen der hervorragende Teamgeist und die hohen Qualitätsniveaus; innerhalb der Praxis die Arbeitsphilosphie meines Teams.<br><br>GP-525: 11 - 11 (0)	Die Motivation meiner älteren Mitarbeiter und meine eigene.<br><br>D-420: 22 - 22 (0)
	der Gedanke selber mal so behandelt zu werden, wie ich es als angemessen sehe<br><br>GP-158: 11 - 11 (0)	Gute Pflegekräfte.<br><br>D-427: 8 - 8 (0)
	meine MFA<br><br>GP-506: 8 - 8 (0)	Liebevolle Angehörige (sowohl im Pflegeheim als auch in der häußlichen Betreuung)

	Specialists	General practitioners	Dentists
			D-427: 8 - 8 (0)<br><br>Meine eigenen motivierten Mitarbeiter,die die Versorgung dieser vulnerablen Patientengruppe als Bereicherung ihrer Arbeit ansehen.<br><br>D-427: 8 - 8 (0)<br><br>Praxisteam<br><br>D-432: 8 - 8 (0)<br><br>Ein gutes Team<br><br>D-433: 8 - 8 (0)<br><br>Mein Gewissen<br><br>D-434: 8 - 8 (0)<br><br>Mein Team.<br><br>D-447: 13 - 13 (0)<br><br>Meine Grundmotivation:<br>auch Menschen am Ende ihres Lebensweges eine ausreichende zahärztliche Versorgung zu ermöglichen.<br>Wer gibt, dem wird gegeben.<br>Es ist eine wechelhafte Beziehung von Freude und Dankbarkeit.<br><br>D-451: 18 - 21 (0)
Begegnung mit den Patient:innen	Patienten selber bei Einsicht, Einsicht der Angehörigen<br><br>S-232: 8 - 8 (0)	informed consent<br>GP-192: 18 - 18 (0)<br><br>Die Patienten haben alle etwas zu erzählen, fast niemand hört ihnen noch zu. Ich durfte ganz wunderbare Menschen kennenlernen und von ihnen lernen.<br>GP-198: 10 - 10 (0)<br><br>Bisherige Erfolge! Sehe in ihnen häufig meine Eltern.<br>GP-201: 8 - 8 (0)<br><br>intensive Gespräche mit den Patienten<br>GP-202: 19 - 19 (0)<br><br>Akzeptanz der Patienten der vorgeschlagenen Lösungen	-Das Vertrauen, darin, dass der Patient und der Behandler am besten wissen, was richtig und gut für den Patienten ist. und dass manchmal die Empfehlungen der Fachgesellschaften nicht für jedes Individuum ideal sind und man dann auch davon abweichen darf und sollte<br>(Beispiel: Nach sachlichen Kriterien ist die Prothese des Patienten absolut unpassen, die Zähne müssten erneuert werden und sie sieht zusätzlich auch noch grauenvoll aus. Der Patient kommt aber wunderbar zurecht, kann sogar die härtesten Möhren damit knacken und ist absolut zufrieden.<br>Würde man ihm jetzt eine neue Prothese erstellen nach fachlich korrekten Vorgaben, dann wäre es sehr gut möglich, dass der Patient sich damit nicht anfreunden kann, nichts mehr kauen kann und sehr unglücklich wird, weil seine Anpassungsfähigkeit im Alter einfach nicht mehr so gegeben ist, wie in jungen Jahren.<br>Also lässt man den Patienten glücklich und zufrieden mit seiner alten Prothese weiter kauen.

	Specialists	General practitioners	Dentists
		GP-244: 20 - 20 (0)<br><br>das Gespräch,<br>GP-245: 8 - 8 (0)<br><br>Setting<br>GP-275: 10 - 10 (0)<br><br>große Hilfe wäre mehr Realismus bei Pat., die sich selbst durch die "rosa-rote Brille" wahrnehmen<br><br>GP-353: 13 - 13 (0)<br><br>ein stabiles Umfeld<br><br>GP-508: 10 - 10 (0)<br><br>soziales Umfeld der Patienten<br><br>GP-520: 18 - 18 (0)<br><br>und natürlich das familiäre Umffeld<br><br>GP-514: 8 - 8 (0)<br><br>- Einsicht des Pat.<br><br>GP-524: 15 - 15 (0)	D-189: 20 - 23 (0)<br><br>Einsicht der Patienten, Pflegepersonal und auch durch die Familienangehörigen.<br><br>D-439: 13 - 13 (0)<br><br>Die Dankbarkeit der Patienten<br><br>D-454: 10 - 10 (0)<br><br>gute Aufklärung<br><br>D-521: 15 - 15 (0)<br><br>Ich kenne die patienten meist schon seit 25 Jahren<br><br>D-521: 18 - 18 (0)
(Gesundheitl.) Zustand der Patient:innen	Mobilität des Pat<br><br>S-499: 8 - 8 (0)	große Hilfe wäre mehr Realismus bei Pat., die sich selbst durch die "rosa-rote Brille" wahrnehmen<br><br>GP-353: 13 - 13 (0)	Mobilität des Patienten, Pflegekraft, manuelle Geschicklichkeit der Patienten<br><br>D-376: 7 - 7 (0)<br><br>geistig und körperlich fitte ü 80 kann ich optimal behandeln<br><br>D-422: 8 - 8 (0)
Interaktion mit weiteren Stakeholdern	Begleitpersonen<br><br>S-163: 8 - 8 (0)<br><br>Kollegen, Pflegedienste, Altenheime, Angehörige<br><br>S-175: 9 - 9 (0)	Angehörige<br><br>GP-192: 17 - 17 (0)<br><br>intensiver Austausch mit Betreuungspersonen<br><br>GP-202: 18 - 18 (0)<br><br>Vorhanden Sie von Unterstützung der Familie	Gespräche mit Partnern oder "Kindern" , damit zu HAuse in Ruhe die Planungen gemeinsam besprochen werden können -. Verunsicherungen können so abgebaut werden<br><br>D-361: 9 - 9 (0)<br><br>Kommunikation mit dem Betreuer, Familienangehörige, Partner<br><br>D-383: 9 - 9 (0)

	Specialists	General practitioners	Dentists
	Praxisteam, Angehörige, Pflegende<br><br>S-221: 8 - 8 (0)<br><br>Miteinbeziehung der Angehörigen,<br><br>S-226: 13 - 13 (0)<br><br>Angehörige, Pflegepersonal,<br><br>S-231: 8 - 8 (0)<br><br>Patienten selber bei Einsicht, Einsicht der Angehörigen<br><br>S-232: 8 - 8 (0)<br><br>Begleitperson<br><br>S-499: 8 - 8 (0)	GP-244: 19 - 19 (0)<br><br>Praxisteam und externe Mitversorger, z.B. Pat.-Familie/Pflegedienst/Heimpersonal<br><br>GP-353: 8 - 8 (0)<br><br>gutes Verhältnis zu den Pflegediensten (leider schlechter werdend)<br><br>GP-518: 16 - 16 (0)	Familiäre Unterstützung und Anwesenheit bei Entscheidungen und Behandlungen<br><br>D-395: 10 - 10 (0)<br><br>Mitarbeit der Angehörigen und des Pflegepersonals von Altenheim.<br><br>D-399: 9 - 9 (0)<br><br>Verwandschaft oder Pfleger<br><br>D-436: 8 - 8 (0)<br><br>Einsicht der Patienten, Pflegepersonal und auch durch die Familienangehörigen.<br><br>D-439: 13 - 13 (0)<br><br>Begleitpersonen, die einen Bezug zu den Patienten haben<br><br>D-449: 8 - 8 (0)<br><br>die Kinder begleite ihre betagten Eltern<br>D-479: 10 - 10 (0)
Zeit	ausreichend Zeit<br><br>S-499: 8 - 8 (0)	längere Zeitfenster für Termine zur Therapieplanung<br><br>GP-192: 19 - 19 (0)<br><br>Berücksichtigung des Gesundheitssystems der hohen Zeitaufwand dieser Patienten Gruppe (während aber auch nach der Sprechstunde)<br><br>GP-244: 21 - 21 (0)<br><br>der Zeitaufwand,<br><br>GP-245: 8 - 8 (0)	und Zeit, um auf die Pat. einzugehen.<br><br>D-433: 8 - 8 (0)
Inter-disziplinäre Zusammen-arbeit	Kollegen, Pflegedienste, Altenheime, Angehörige<br><br>S-175: 9 - 9 (0)<br><br>Allgemeinmediziner	guter Kontakt zu Fachkollegen, Therapeuten, Assistenzberufen<br><br>GP-202: 17 - 17 (0)	sehr enge Kontakte in einer Landpraxis, man kennt sich<br><br>D-521: 16 - 16 (0)

	Specialists	General practitioners	Dentists
	Internisten<br><br>S-216: 11 - 12 (0)<br><br>Rücksprache mit anderen behandelnden Ärzten<br><br>S-226: 13 - 13 (0)<br><br>Pflegepersonal, ärztliche Kollegen<br><br>S-231: 8 - 8 (0)<br><br>Netzwerk<br><br>S-470: 8 - 8 (0)	DMP Programme und in normalen (nicht Corona) Zeiten eine VeraH<br><br>GP-271: 8 - 8 (0)	
Lokale Strukturen und Angebote	sletener ortsständige Stellen, manchmal die Kirchengemeinden<br><br>S-196: 13 - 13 (0)	Sofern es die Patienten erlauben, versuche ich sie in ein Netzwerk einzubinden: Hausbesuch durch mich oder meine EVAS, Tagespflege, Angebote durch die Caritas, Spaziergänge mit Ehrenamtlichen etc.<br><br>GP-198: 10 - 10 (0)<br><br>Unterstützungseinrichtungen vor Ort (z.B. ambulante Pflege, Tgaespflegeeinrichtungen, ambulanter Hospizdienst)<br><br>GP-520: 19 - 19 (0)<br><br>- gute soziale Strukturen (Pflegedienste/Angehörige)<br><br>GP-524: 14 - 14 (0)<br><br>- Praxisstruktur<br><br>GP-524: 16 - 16 (0)	? Die Frage sollt sein wer! Politisch muss sich etwas tun, mehr Ärzte sollten dazu bereit sein Patienten zu versorgen. Entbürokratisierung ist wichtig. Genau so die Publizieren von Hygienemaßnahmen und der Möglichekit der Hausbesuche.-<br><br>D-374: 9 - 9 (0)
Unterstützende Tools und Programme	Medikationsplan<br><br>S-499: 8 - 8 (0)	Hilfe bei der Kommunikation und bei der praktischen Umsetzung der VerOrdnungen<br><br>GP-219: 8 - 8 (0)	

	Specialists	General practitioners	Dentists
		DMP Programme und in normalen (nicht Corona) Zeiten eine VeraH<br><br>GP-271: 8 - 8 (0)<br><br>Unterstützung durch z. B. Priscus-Liste<br><br>GP-512: 15 - 15 (0)	
Nicht möglich		gar nicht<br><br>GP-508: 11 - 11 (0)	
Vertrautes Umfeld		Vertraute Umgebung<br>GP-244: 23 - 23 (0)<br><br>vertrautes Umfeld<br>GP-310: 9 - 9 (0)	Ein vertrautes Umfeld<br>D-395: 11 - 11 (0)<br><br>an der vertrauten Person/Arzt/Zahnarzt<br>D-415: 9 - 9 (0)
Zeitnahe Kontakt-möglichkeit	leichterer Zugang zur Versorgung<br><br>S-231: 9 - 9 (0)	Durch kurfristige Ansprechbarkeit<br><br>GP-525: 12 - 12 (0)<br><br>Mediziner / Hausarzt als gut ereichbarer Ansprechpartner, der die Versorgung führt und terminiert<br><br>GP-158: 12 - 13 (0)<br><br>- gute Erreichbarkeit bei Problemen<br><br>GP-524: 17 - 17 (0)	zeitnahe Betreuung,<br><br>D-451: 22 - 22 (0)
Vorausschauende Versorgung	Überblicken der Gesamtsituation<br><br>S-221: 9 - 9 (0)	Mediziner / Hausarzt als gut ereichbarer Ansprechpartner, der die Versorgung führt und terminiert<br><br>GP-158: 12 - 13 (0)	Regelmäßige Kontrollen<br>D-439: 15 - 15 (0)<br><br>Die Patienten werden entsprechend ihres Alteres, Behinderung und gesamter Situation Wahrgenommen<br><br>D-449: 9 - 9 (0)<br><br>gesicherte Rahmenbedingungen im Gesundheitswesen und der Versorgung.<br>D-451: 22 - 22 (0)
Hilfe und Unterstützung	zustandsadäqaute Transporte ohne Sturzgefahr und Begleitpersonen, die das sicherstellen.<br><br>S-175: 10 - 10 (0)	gute Aufklärung incl. der Angehörigen<br><br>GP-192: 20 - 20 (0)	Hilfeleistungen anbieten (Personal geschult) schon auch beim Betreten der Praxis bis zum Verlassen<br>D-371: 12 - 12 (0)

	Specialists	General practitioners	Dentists
	Hilfe bei der Medikamenteneinnahme.<br><br>S-175: 10 - 10 (0)<br><br>durch eine Verbesserung von Gehfähigkeit,Ausgleich von Defiziten, Motivation zur Nutzung von Hilfsmitteln<br><br>S-196: 14 - 14 (0)	Pflegehilfsmittel, ggfs. Reha-Maßnahmen<br><br>GP-353: 9 - 9 (0)<br><br>- unterstützende Hilfsmittel<br><br>GP-524: 18 - 18 (0)	Hilfe durch das Team und den Behandler; behindertengerechte Praxis<br>D-384: 9 - 9 (0)<br><br>Rücksichtnahme auf vorhandene Einschränkungen. Assistenz bei Verrichtungen (Ankleiden, Treppenlift...)<br>D-399: 10 - 10 (0)<br><br>Eine liebenswerte Bezugsperson<br>D-427: 9 - 9 (0)<br><br>Geh Unterstützung geben,<br>D-434: 9 - 9 (0)<br><br>bequeme altersgerechte Behandlungsstühle<br>D-479: 11 - 11 (0)
Gute Kommunikation, Beratung und Gespräche	Information, Beratung, persönliches Vertrauen<br><br>S-163: 9 - 9 (0)<br><br>ausreichend Zeit für Gespräche und Durchführung der Maßnahmen um die altersbedingten Einschränkungen berücksichtigen zu können<br><br>S-169: 9 - 9 (0)<br><br>Ruhe, Zuverlässigkeit, Gründlichkeit. Patientinnen und Patienten dort "abholen", wo sie sich geistig und körperlich befinden.<br><br>S-223: 12 - 12 (0)<br><br>ruhige, langsame Ansprache des Patienten, ins gesicht schauen, Verwendung deutscher Begriffe<br><br>S-226: 14 - 14 (0)<br><br>Gute Beratung<br><br>S-232: 9 - 9 (0)<br><br>Erklärungen<br><br>S-322: 9 - 9 (0)	gute Aufklärung incl. der Angehörigen<br><br>GP-192: 20 - 20 (0)<br><br>durch Gesprächsführung: Verstehen, was der Patient befürchtet und wünscht,<br><br>GP-202: 20 - 20 (0)<br><br>Gute und freundliche Kommunikation<br><br>GP-219: 9 - 9 (0)<br><br>Aufklärung, Empathie, Erlichkeit, Zuneigung<br><br>GP-245: 9 - 9 (0)<br><br>Aufklärung<br><br>GP-253: 12 - 12 (0)<br><br>Beratung<br><br>GP-353: 9 - 9 (0)<br><br>schriftliche Therapieanweisungen,<br>klare Kommunikation<br><br>GP-520: 20 - 21 (0)	Patienten ernst nehmen, wahrnehmen und ihn sehen. Zeit, Zuwendung, Zuhören, Erklären, Zeigen.<br><br>D-189: 24 - 24 (0)<br><br>entsorechendes framing<br><br>D-234: 10 - 10 (0)<br><br>Langsames Vorgehen, keine zeitlicher Stress, viele Erklärungen<br><br>D-374: 10 - 10 (0)<br><br>Erklären, erklären erklären<br><br>D-398: 9 - 9 (0)<br><br>aufmerksames Zuhören welche Beschwerden oder Bedürfnisse bestehen.<br><br>D-423: 9 - 9 (0)<br><br>durch einen Umgamg mit den Patienten auf Augenhöhe<br><br>D-435: 9 - 9 (0)<br><br>Besuche, Gespräche<br><br>D-436: 9 - 9 (0)

	Specialists	General practitioners	Dentists
	Wissen vermitteln und gut beraten<br><br>S-325: 9 - 9 (0)<br><br>ausreichend über die medizinischen Sachverhalte zu informieren<br><br>S-499: 9 - 9 (0)<br><br>Fundierte Erklärungen.<br><br>S-516: 9 - 9 (0)	gute Kommunikation<br><br>GP-512: 16 - 16 (0)	
Zuwendung und wertschätzendes Verhalten, Beziehungs-aufbau	Information, Beratung, persönliches Vertrauen<br><br>S-163: 9 - 9 (0)<br><br>Empathie<br><br>S-221: 9 - 9 (0)<br><br>auftretten, Verhalten bei der Anamneseerhebung<br><br>S-222: 12 - 12 (0)<br><br>Ruhe, Zuverlässigkeit, Gründlichkeit. Patientinnen und Patienten dort "abholen", wo sie sich geistig und körperlich befinden.<br><br>S-223: 12 - 12 (0)<br><br>durch einfühlsame Behandlungen<br><br>S-372: 9 - 9 (0)<br><br>Autentisches, engagiertes behandeln<br><br>S-470: 9 - 9 (0)	Zuwendung, Anteilnahme an ihren Sorgen und Befürchtungen<br><br>GP-198: 11 - 11 (0)<br><br>Vertrauen in meine Person.<br><br>GP-201: 9 - 9 (0)<br><br>durch Gesprächsführung: Verstehen, was der Patient befürchtet und wünscht,<br><br>GP-202: 20 - 20 (0)<br><br>Aufklärung, Empathie, Erlichkeit, Zuneigung<br><br>GP-245: 9 - 9 (0)<br><br>weil ich Ihnen zugewandt zuhöre<br><br>GP-271: 9 - 9 (0)<br><br>Zuwendung<br><br>GP-275: 12 - 12 (0)	Patienten ernst nehmen, wahrnehmen und ihn sehen. Zeit, Zuwendung, Zuhören, Erklären, Zeigen.<br><br>D-189: 24 - 24 (0)<br><br>Behandlertreue<br><br>D-305: 8 - 8 (0)<br><br>Verständnis zeigen , Zeit investieren<br><br>D-361: 10 - 10 (0)<br><br>langjähriges Vertrauen<br><br>D-362: 9 - 9 (0)<br><br>Emphatie<br><br>D-367: 16 - 16 (0)<br><br>berufliche Kompetenz, pers. Umgang<br><br>D-376: 8 - 8 (0)<br><br>Aufmerksamkeit geben<br><br>D-382: 9 - 9 (0)<br><br>würdevoller Umgang, Zeitinestition während der Sitzungen<br><br>D-383: 10 - 10 (0)

Specialists	General practitioners	Dentists
		freundlicher Umgang<br>D-384: 9 - 9 (0)<br>intensive Patientenbetreuung<br>D-407: 9 - 9 (0)<br>Vertrauen aufbauen<br>D-416: 18 - 18 (0)<br>Vertrauen in die Kompetenz.<br>D-420: 24 - 24 (0)<br>Freundliche Aufnahme durch Mitarbeiter.<br>D-420: 24 - 24 (0)<br>man kennt sich schon lange und der Patient vertraut mir<br>D-422: 9 - 9 (0)<br>Mitgefühl, Respekt, Nähe und Körperkontakt bei Ansprache,<br>D-433: 9 - 9 (0)<br>Zuhören<br>D-434: 9 - 9 (0)<br>, ruhig und gelassen sein<br>D-434: 9 - 9 (0)<br>durch einen Umgamg mit den Patienten auf Augenhöhe<br>D-435: 9 - 9 (0)<br>wertschätzender Umgang<br>D-447: 14 - 14 (0)

	**Specialists**	**General practitioners**	**Dentists**
			Einfühlungsvermögen<br><br>D-454: 11 - 11 (0)<br><br>gute Arzt Patienten Bindung<br><br>D-521: 19 - 19 (0)
Ausreichend Zeit	ausreichend Zeit für Gespräche und Durchführung der Maßnahmen um die altersbedingten Einschränkungen berücksichtigen zu können<br><br>S-169: 9 - 9 (0)		Patienten ernst nehmen, wahrnehmen und ihn sehen. Zeit, Zuwendung, Zuhören, Erklären, Zeigen.<br><br>D-189: 24 - 24 (0)<br><br>Verständnis zeigen , Zeit investieren<br><br>D-361: 10 - 10 (0)<br><br>Langsames Vorgehen, keine zeitlicher Stress, viele Erklärungen<br><br>D-374: 10 - 10 (0)<br><br>würdevoller Umgang, Zeitinestition während der Sitzungen<br><br>D-383: 10 - 10 (0)<br><br>ruhige Behandlung ohne zeitlichen Stress<br><br>D-433: 9 - 9 (0)
Kompetenz und Fortbildungen	Gute Fortbildung<br><br>S-216: 13 - 13 (0)	Gute Mitarbeiter<br><br>GP-514: 9 - 9 (0)	regelmäßige Fortbildungen auch speziell in Bezug auf dieses Patientenklientel<br><br>D-371: 12 - 12 (0)<br><br>berufliche Kompetenz, pers. Umgang<br><br>D-376: 8 - 8 (0)<br><br>Vertrauen in die Kompetenz.<br><br>D-420: 24 - 24 (0)

	Specialists	General practitioners	Dentists
Gute Kommunikation, Beratung und Gespräche	mimisch und gestisch Empathie zeigen, erst Zuhören - dann das Ggesgeistigerehörte in den PC tippen<br><br>S-226: 15 - 15 (0)<br><br>ausführliche beratung<br><br>S-325: 10 - 10 (0)	Durch Gespräche<br><br>GP-253: 13 - 13 (0)<br><br>ausreichend Zeit für Gespräche<br><br>GP-520: 22 - 22 (0)	altersgerechte Sprache und Aufklärung;<br><br>D-384: 10 - 10 (0)<br><br>intensive Begleitung der Behandlung, Telefonate vor/nach Behandlung, Terminvereinbarung<br><br>D-407: 10 - 10 (0)<br><br>jeden Arbeitsschritt/Behandlungsablauf auf "Augenhöhe"kommunizieren; auf die Bedürfnisse eingehen<br><br>D-416: 19 - 19 (0)<br><br>man redet miteinander auch mehr als nur über Krankheit<br><br>D-422: 10 - 10 (0)<br><br>, viel erklären<br><br>D-433: 10 - 10 (0)<br><br>Gespräche führen, die über die Behandlung hinaus gehen<br><br>D-439: 16 - 16 (0)<br><br>langsames, verständliches Sprechen<br><br>D-479: 12 - 12 (0)
Nicht möglich	kann ich nicht gewährleisten<br><br>S-232: 10 - 10 (0)		

	Specialists	General practitioners	Dentists
Zuwendung und wertschätzendes Verhalten, Beziehungs-aufbau	Wertschätzung und Verständnis<br><br>S-163: 10 - 10 (0)<br><br>durch persönlcihce Wertschätzung und ehrenamtliche Arbeit<br><br>S-196: 15 - 15 (0)<br><br>auch über die Medizin hinausgehende Beobachtung<br><br>S-221: 10 - 10 (0)<br><br>zuhören<br><br>S-222: 13 - 13 (0)<br><br>Anamnese, insbes. Sozialanamnese, Schwätzchen über Herkunft etc.<br><br>S-223: 13 - 13 (0)<br><br>mimisch und gestisch Empathie zeigen, erst Zuhören - dann das Ggesgeistigerehörte in den PC tippen<br><br>S-226: 15 - 15 (0)<br><br>Wertschätzung<br><br>S-322: 10 - 10 (0)<br><br>durch eine freundliche Begrüßung und menschliche Behandlung<br><br>S-372: 10 - 10 (0)<br><br>Zuwendung<br><br>S-469: 10 - 10 (0)<br><br>empathisch und mit Respekt behandeln<br><br>S-499: 10 - 10 (0)	Ausreden lassen. Ehrlichkeit.<br><br>GP-198: 12 - 12 (0)<br><br>durch Gespräche<br><br>GP-202: 21 - 21 (0)<br><br>Gute und freundliche Kommunikation<br><br>GP-219: 10 - 10 (0)<br><br>Würdigung des Alters,<br><br>GP-244: 24 - 24 (0)<br><br>mehr Zeit und Aufmerksamkeit für sie nehmen/ planen<br><br>GP-244: 24 - 24 (0)<br><br>Durch Gespräche<br><br>GP-253: 13 - 13 (0)<br><br>Zuwendung<br><br>GP-275: 13 - 13 (0)<br><br>Zuwendung<br><br>GP-353: 10 - 10 (0)<br><br>Zuhören trotz Zeitdruck<br><br>GP-508: 12 - 12 (0)<br><br>Gesprächsführung, auch umständlich vorgebrachte Belange erst nehmen und adäquat kommunizieren und zwar nicht "über den Kopf" hinweg<br><br>GP-518: 18 - 18 (0)<br><br>ausreichend Zeit für Gespräche<br><br>GP-520: 22 - 22 (0)	gute ZFA<br><br>D-305: 9 - 9 (0)<br><br>würdevoller Umgang<br><br>D-361: 11 - 11 (0)<br><br>Wertschätzung des Geleisteten<br><br>D-367: 17 - 17 (0)<br><br>alle Mitarbeiter müssen dieses so wahrnehmen<br><br>D-371: 13 - 13 (0)<br><br>Sollte ein Arzt die Fähigkeit der Empathie nicht beherrschen, sollte er eigentlich kein Arzt sein.<br><br>D-374: 11 - 11 (0)<br><br>Pers. Umgang, eingehen auf Probleme<br><br>D-376: 9 - 9 (0)<br><br>Zeit für Gespräche und Behandlung haben<br><br>D-382: 10 - 10 (0)<br><br>würdevoller Umgang,<br><br>D-383: 11 - 11 (0)<br><br>Respekt erweisen durch direkte Ansprache; auf die Probleme eingehen und keine rechthaberische Aktionen nur nach Lehrbuch<br><br>D-384: 10 - 10 (0)<br><br>Zu zu hören und Zeit zu nehmen<br><br>D-395: 12 - 12 (0)<br><br>selbstwertgefühl steigern<br><br>D-398: 10 - 10 (0)

	Specialists	General practitioners	Dentists
	Wertschätzende Kommunikation<br><br>S-516: 10 - 10 (0)	Durch Respektierung ihrer Person und ihrer biographischen Hintergründe<br><br>GP-525: 13 - 13 (0)<br><br>respektvoller Umgang<br><br>GP-512: 17 - 17 (0)<br><br>Zeit zum Gespräch<br><br>GP-514: 10 - 10 (0)<br><br>- Zuhören, Probleme ernst nehmen<br><br>GP-524: 19 - 19 (0)	Respektvoller Umgang<br><br>D-399: 11 - 11 (0)<br><br>Respekt<br><br>D-420: 25 - 25 (0)<br><br>freundlicher Empfang und umgang mit dem Patienten.<br><br>D-423: 10 - 10 (0)<br><br>Zuhören wie bei allen Patienten<br><br>D-432: 10 - 10 (0)<br><br>Respekt bei der Behanldung, Nähe und Körperkontakt<br><br>D-433: 10 - 10 (0)<br><br>Gespräche führen, die über die Behandlung hinaus gehen<br><br>D-439: 16 - 16 (0)<br><br>wertschätzender Umgang<br><br>D-447: 15 - 15 (0)<br><br>aufrichtige Anteilnahme und Respekt<br><br>D-451: 23 - 23 (0)
Ausreichend Zeit	Auf die Bedürfnisse eingehen, Zeit haben<br><br>S-216: 14 - 14 (0)	mehr Zeit und Aufmerksamkeit für sie nehmen/ planen<br><br>GP-244: 24 - 24 (0)<br><br>weil sie jederzeit einen Termin buchen können	Zeit für Gespräche und Behandlung haben<br><br>D-382: 10 - 10 (0)<br><br>Zu zu hören und Zeit zu nehmen

	Specialists	General practitioners	Dentists
	bessere Vergütung für die Ärzte, da mehr Zeitaufwand<br><br>S-231: 10 - 10 (0)<br><br>ausführliche beratung<br><br>S-325: 10 - 10 (0)<br><br>Zeitinvestitution, Hilfestellungen bei probleme<br><br>S-470: 10 - 10 (0)	GP-271: 10 - 10 (0)<br><br>Zuhören trotz Zeitdruck<br><br>GP-508: 12 - 12 (0)<br><br>ausreichend Zeit für Gespräche<br><br>GP-520: 22 - 22 (0)<br><br>Zeit zum Gespräch<br><br>GP-514: 10 - 10 (0)	D-395: 12 - 12 (0)<br><br>Zeit nehmen<br><br>D-434: 10 - 10 (0)<br><br>Zeit für den Patienten haben<br><br>D-449: 10 - 10 (0)<br><br>Ausreichen Zeit<br><br>D-454: 12 - 12 (0)<br><br>sich Zeit nehmen<br><br>D-521: 20 - 20 (0)
Eingehen auf indiv. Bedürfnisse & Wünsche, ganzheitl. Sichtw.	Auf die Bedürfnisse eingehen, Zeit haben<br><br>S-216: 14 - 14 (0)<br><br>Anamnese, insbes. Sozialanamnese, Schwätzchen über Herkunft etc.<br><br>S-223: 13 - 13 (0)	Berücksichtigung der speziellen Situation alter Menschen<br><br>GP-192: 21 - 21 (0)<br><br>Eingehen auf ihre Fragen.<br><br>GP-201: 10 - 10 (0)	Wenn man auf ihre Bedürfnisse eingeht und die Wünsche, die verbal oder nonverbal geäußert werden nicht übergeht.<br><br>D-189: 25 - 25 (0)<br><br>Der Patient wird als Persönlichkeit respektiert und nicht als Fall gesehen<br><br>D-362: 10 - 10 (0)

	Specialists	General practitioners	Dentists
	Zeitinvestitution, Hilfestellungen bei probleme<br><br>S-470: 10 - 10 (0)	Gesprächsführung, auch umständlich vorgebrachte Belange erst nehmen und adäquat kommunizieren und zwar nicht "über den Kopf" hinweg<br><br>GP-518: 18 - 18 (0)<br><br>Therapie, Diagnostik und Fürsorge individuell gestalten<br><br>GP-158: 14 - 14 (0)<br><br>- Zuhören, Probleme ernst nehmen<br><br>GP-524: 19 - 19 (0)	Pers. Umgang, eingehen auf Probleme<br><br>D-376: 9 - 9 (0)<br><br>Respekt erweisen durch direkte Ansprache; auf die Probleme eingehen und keine rechthaberische Aktionen nur nach Lehrbuch<br><br>D-384: 10 - 10 (0)<br><br>jeden Arbeitsschritt/Behandlungsablauf auf "Augenhöhe"kommunizieren; auf die Bedürfnisse eingehen<br><br>D-416: 19 - 19 (0)<br><br>man redet miteinander auch mehr als nur über Krankheit<br><br>D-422: 10 - 10 (0)<br><br>Direkt angesprochen werden auf Ihre Wünsche, auch wenn eine Begeitperson oder Bezugsperson anwesend ist<br><br>D-427: 10 - 10 (0)<br><br>durch Behandlungen die in Abprache mit den Patienten erarbeitet und durchgeführt werden<br><br>D-435: 10 - 10 (0)<br><br>auf die Pat.eingehen<br><br>D-436: 10 - 10 (0)
Soziale Ein-gebundenheit	mache ich sowieso immer, sehe das aber im Umfeld öfter nicht gewährleistet<br><br>S-175: 11 - 11 (0)<br><br>durch persönlcihce Wertschätzung und ehrenamtliche Arbeit<br><br>S-196: 15 - 15 (0)	Soziale Anbindung<br><br>GP-244: 24 - 24 (0)<br><br>soziale Kontakte<br><br>GP-310: 10 - 10 (0)	
Nicht (immer) möglich	das kann ich leider nicht immer gewührleisten, da ich manchmal die Rolle derjenigen habe, die zum Schutze des PAtineten auch Maßnahmen	In vielen Fällen bin ich allerdings der Überzeugung, dass Autonomie bei extrem hilfsbedürftigen alten Menschen oft im Kontext guter heimpflegerischer Versorgungf besser	? Sicherlich kann ein Zahnarzt keine Autonomie herstellen. Wir können eine Gewährleistung der Versorgung sicher stellen und werden dabei nur durch unsere Standesvertreter unterstützt, jedoch nicht durch die Politik,

	Specialists	General practitioners	Dentists
	ergreifen muss, z.B. Anregung und Befürwortung einer Betreuung, manchmal sind offene Worte dabei unentbehrlich<br><br>S-196: 16 - 16 (0)<br><br>kann ich nicht gewährleisten<br><br>S-232: 11 - 11 (0)	gelingt als daheim; ein alter Mensch, dessen einzige Ansprechpartner maximal dreimal tgl. amb. Pflegepersonen sind, ist für mich nicht automatisch autonomer.<br><br>GP-525: 14 - 14 (0)	D-374: 12 - 12 (0)
Eingehen auf indiv. Bedürfnisse & Wünsche, ganzheitl. Sichtw.		Durch Respektierung ihrer Lebens(endes)ziele.<br><br>GP-525: 14 - 14 (0)	Wünsche des Patienten Erden respektiert<br><br>D-362: 11 - 11 (0)<br><br>als Mensch wahrnehmen<br><br>D-382: 11 - 11 (0)
Gute Kommunikation und Beratung	das kann ich leider nicht immer gewührleisten, da ich manchmal die Rolle derjenigen habe, die zum Schutze des PAtineten auch Maßnahmen ergreifen muss, z.B. Anregung und Befürwortung einer Betreuung, manchmal sind offene Worte dabei unentbehrlich<br>S-196: 16 - 16 (0)<br><br>gute Beratung zu den Vorteilen von Hörgeräten<br>S-325: 11 - 11 (0)<br><br>Zeit für den Patienten<br>S-469: 11 - 11 (0)	gute Beratung zur eigenen Entscheidung<br><br>GP-514: 11 - 11 (0)<br><br>- gute Aufklärung, Unterstützung durch soziale Strukturen<br><br>GP-524: 21 - 21 (0)	Zeit nehmen und so weit wie möglich aufklären und berten<br><br>D-234: 12 - 12 (0)<br><br>Hilfestellung bei Therapieentscheidungen.<br><br>D-423: 11 - 11 (0)<br><br>Hör- und Verständnisfähigkeit muss sicher gestellt sein<br><br>D-449: 11 - 11 (0)
Wertschätzender Umgang	durch Versuch der Empathie,<br><br>S-223: 14 - 14 (0)		würdevoller Umgang<br><br>D-361: 11 - 11 (0)<br><br>respektvoller Umgang<br><br>D-383: 12 - 12 (0)<br><br>wertschätzender Umgang<br><br>D-447: 16 - 16 (0)

	Specialists	General practitioners	Dentists
Entscheidungs-möglichkeiten schaffen	Scheidungsfreiheit belassen<br><br>S-163: 11 - 11 (0)<br><br>durch eigene Entscheidungen bei der Wahl der Therapie (z.B. wenn es um eine Hörgeräteversorgung geht)<br><br>S-372: 11 - 11 (0)<br><br>Entscheidungsfreiheit belassen<br><br>S-499: 11 - 11 (0)<br><br>Einbindung in Entscheidungsprozesse unter Berücksichtigung o.g. Faktoren<br><br>S-516: 11 - 11 (0)	Entscheidungskonsens herbeiführen, wo immer möglich<br><br>GP-353: 11 - 11 (0)<br><br>gute Beratung zur eigenen Entscheidung<br><br>GP-514: 11 - 11 (0)	Alternativen anbieten. Immer dem Patienten vermitteln: Du entscheidest, ich biete an.<br><br>D-189: 26 - 26 (0)<br><br>In Entscheidungen mit einzubeziehen<br><br>D-395: 13 - 13 (0)<br><br>Entscheidung über Vorhaben eigenbestimmt.<br><br>D-399: 12 - 12 (0)<br><br>bei der Behandlungsplanung Behandlungsalternativen aufzeigen und Entscheidung selber treffen lasen<br><br>D-416: 20 - 20 (0)<br><br>Wahl der Entscheidung lassen.<br><br>D-420: 26 - 26 (0)<br><br>mit entscheiden<br><br>D-434: 11 - 11 (0)
Ermutigen und ohne Bevormundung so viel wie nötig unterstützen	Vermeiden von Bevormundung und Einschränkung durch mediz. Maßnahmen<br><br>S-221: 11 - 11 (0)<br><br>den Patienten Mut machen weiter an ihre Gesundheit zuarbeiten<br><br>S-222: 14 - 14 (0)	mögliche Hilfsmittel und anderweitige Unterstützungen ansprechen und bei Bedarf nahelegen<br><br>GP-245: 11 - 11 (0)<br><br>altersadaptierte Medikation, regelm. körperliche Ertüchtigung, gestige Beschäftigung<br><br>GP-518: 19 - 19 (0)<br><br>- Hilfsmittel/Umbauten<br><br>GP-524: 20 - 20 (0)	nicht bevormunden, aber Hilfestellung anbieten<br><br>D-371: 14 - 14 (0)<br><br>ambulante Versorgung möglich, selbstbestimmtes Handeln der Patienten<br><br>D-376: 10 - 10 (0)<br><br>soviel wie möglich eigenständig erledigen zu können ohne das Gefühl in ein Pflegeheim abgeschoben zu werden um dort bis zum ende verwahrt zu werden<br><br>D-398: 11 - 11 (0)<br><br>Ihnen die Unterstützung angeboten wird, die Sie brauchen.ohne Sie komplett zu bevormunden.

	Specialists	General practitioners	Dentists
			D-427: 11 - 11 (0)<br><br>ähnlich wie bei Kindern kann man sie erst selbst machen lassen und dann Hilfe anbieten, oder nur Hilfe geben, bei der sie wirklich gebraucht wird, alles was noch möglich ist, sollten sie selbst erledigen<br><br>D-433: 11 - 11 (0)
Fähigkeiten erhalten und ausbauen, Prävention	den Patienten Mut machen weiter an ihre Gesundheit zuarbeiten<br><br>S-222: 14 - 14 (0)<br><br>aber auch mit den möglichen Medikamenten und Hilfsmitteln.<br><br>S-223: 14 - 14 (0)<br><br>geistiges Fördern im häuslichen Umfeld, lange die Selbständigkeit lassen<br><br>S-226: 16 - 16 (0)<br><br>gute Beratung zu den Vorteilen von Hörgeräten<br><br>S-325: 11 - 11 (0)	Wenn sie ihre Krankheiten akzeptieren und ihre Medikamente einnehmen<br><br>GP-201: 11 - 11 (0)<br><br>Erhalt und Ausbau der vorhandenen Fähigkeiten<br><br>GP-202: 22 - 22 (0)<br><br>Betonung und Verstärkung der Fähigkeiten, die sie noch können. Motivierung und Unterstützung der Patienten<br><br>GP-244: 25 - 25 (0)<br><br>frühzeitige Interventionen bei Verlust von Fähigjeiten zB Mobilität nach Verletzungen<br><br>GP-508: 13 - 13 (0)	Wiederherstellung der Kaufunktion und Ästhetik, wo es geht;<br><br>D-384: 11 - 11 (0)

	Specialists	General practitioners	Dentists
		altersadaptierte Medikation, regelm. körperliche Ertüchtigung, gestige Beschäftigung<br><br>GP-518: 19 - 19 (0)<br><br>Unterstützung beim selbständigen Leben zu Hause (Hausnotruf, Mobilitätserhaltende Unterstützung)<br><br>GP-520: 23 - 24 (0)<br><br>Eigenständigkeit fördern<br><br>GP-158: 15 - 15 (0)	
Hilfs-, Angebots- und Versorgungs-strukturen	Barrierefreiheit, auch bei der Übermittlung von Informationen und ERfassen der Anamnese durch z.B. Audio-unterstützung oder große Schriften<br><br>S-169: 11 - 11 (0)<br><br>Optimierung der gegebenen Wohn- und Versorgungseinrichtungen<br><br>S-175: 12 - 12 (0)<br><br>Hilfe vor Ort in der häuslichen Umgebung<br><br>S-216: 15 - 15 (0)<br><br>gesellschaftliche veränderungen<br><br>S-217: 16 - 16 (0)<br><br>Folgetermine<br><br>S-322: 11 - 11 (0)	Ausschöpfung aller Möglichkeiten für Hilfsmittel und ambulante Unterstzützung<br><br>GP-192: 22 - 22 (0)<br><br>Netzwerkaufbau zu Hause<br><br>GP-198: 13 - 13 (0)<br><br>Menschen würdige äußere Umstände<br><br>GP-219: 11 - 11 (0)<br><br>Ambulante Pflegedienste zur Unterstützung<br><br>GP-253: 14 - 14 (0)<br><br>weil es ambulante Betreuungsangebote gibt<br><br>GP-271: 11 - 11 (0)<br><br>Personelle Hilfen<br><br>GP-275: 14 - 14 (0)	Teilnahme an der zahnmedizinischen Versorgung ermöglichen<br><br>D-367: 18 - 18 (0)<br><br>Einbindung der Familie<br><br>D-384: 11 - 11 (0)<br><br>Barrierefreiheit<br><br>D-434: 11 - 11 (0)<br><br>Besuche in deren Hausgemeinschaft<br><br>D-436: 11 - 11 (0)<br><br>Unterstützen<br><br>D-439: 17 - 17 (0)

	Specialists	General practitioners	Dentists
	Netzwerk<br><br>S-470: 11 - 11 (0)	Unterstützung durch ambulante Pflegedienste,<br><br>GP-520: 23 - 23 (0)<br><br>Unterstützung beim selbständigen Leben zu Hause (Hausnotruf, Mobilitätserhaltende Unterstützung)<br><br>GP-520: 23 - 24 (0)<br><br>Organisation (nicht-)medizinischer Hilfsmittel, Pflegedienst...<br><br>GP-512: 18 - 18 (0)<br><br>- gute Aufklärung, Unterstützung durch soziale Strukturen<br><br>GP-524: 21 - 21 (0)	
**Grundlegende Werte und Orientierung**			
Präventions-orientierung	in jungen Jahren vorsorgen, um im Alter möglichst wenig medizinische Hilfe zu benötigen<br><br>S-499: 13 - 13 (0)	im Alter erhält man die "Rechnung" für seine Lebensgewohnheiten; aus meiner Sicht geht es Patienten im Alter besser, wenn sie Jahre bis Jahrzehnte zuvor aktiv waren in Sport, sozialem Umfeld, Freunde und Familie, Kunst und Kultur, Ehrenamt und Aufgabe<br>GP-245: 13 - 13 (0)<br><br>von jungenJahren an Gesundheit erhalten: Bewegung, keine Überernährung, nicht Rauchen, nicht zu viel Alkohol<br>GP-353: 12 - 12 (0)	Mehr ProphlyaxeMaßnahmen für Senioren<br>D-367: 19 - 19 (0)<br><br>Mehr bezahlte Prophylaxe<br>D-376: 11 - 11 (0)<br><br>mehr Vorsorge Bonusprogramme<br>D-434: 12 - 12 (0)<br><br>Eine gesündere, nicht so kariogene Kost in den Pflegeheimen<br>D-447: 17 - 17 (0)
Patient:innen-orientierung	je länger diese Gruppe mit maximaler Unterstützung selbstbestimmt leben kann um so besser ist das für die gesamte Gesellschaft und um so beglückender ist es noch dazu<br><br>S-175: 14 - 14 (0)<br><br>von der stationären Geriatire wünsche ich mir mehr Menschlichkeit, mehr Individualität und eine bessere Angehärigenarbeit<br><br>S-196: 7 - 7 (0)	In meiner Rolle als Ärztin fühle ich mich als Fossil. Nichts von dem, weswegen ich vor 33 Jahren angetreten bin, hat noch Bestand. Die Kommerzialisierung unseres Berufsstandes ist eine Schande. Ich sehe in einem Patienten, egal welchen Alters, keinen Wirtschaftsfaktor. Ich behandle Menschen, kein Alter, keine Hautfarbe, keine Nationalität, kein Portemonnaie. Dies wird belächelt, aber eine Medizin ohne Herz oder Empathie wird zur reinen technikverliebten Medizin herabgewürdigt. Meiner Zukunft als Patientin sehe ich mit großer Sorge entgegen.<br>GP-198: 14 - 14 (0)	Auf Bedürfnisse der älteren Menschen eingehen.<br><br>D-382: 12 - 12 (0)<br><br>Sprechende Zahnheilkunde muss endlich bezahlt werden !!!<br><br>D-420: 28 - 28 (0)

	Specialists	General practitioners	Dentists
	selbstverantwortlichkeit fördern und fordern<br><br>S-217: 19 - 19 (0)	Hilfreich für meine tägliche Arbeit wäre, die sprechende Medizin aufzuwerten.<br>GP-198: 15 - 15 (0)<br><br>Ich wünsche mir eine patientenorientierte Medizin, leider geht es nur um Gewinnmaximierung.<br>Die Politik und Wirtschaftsverbände geben sich diesbezüglich alle Mühe. Seit Jahren bin ich in der Medizinpolitik tätig; die Lage der Medizin verändert sich stetig, nur nicht zum besseren.<br>GP-198: 16 - 17 (0)<br><br>Bessere Honorierung der sprechenden Medizin<br>GP-202: 23 - 23 (0)<br><br>Auch und gerade bei den über 80-jährigen ist oft nicht so sehr die medizinische sondern die ärztliche Kompetenz gefordert.<br><br>GP-202: 24 - 24 (0)<br><br>Geriatrie ist mehr als Medikamente verordnen, die den Mediplan ausufern lassen. Es ist ein sehr individualisierter Zweig der Medizin, der den alten Menschen mit seinen individuellen Gebrechen/Einschränkungen auffängt und bestehen lässt.<br><br>GP-525: 16 - 16 (0)	
Empathie	von der stationären Geriatire wünsche ich mir mehr Menschlichkeit, mehr Individualität und eine bessere Angehärigenarbeit<br><br>S-196: 7 - 7 (0)	In meiner Rolle als Ärztin fühle ich mich als Fossil. Nichts von dem, weswegen ich vor 33 Jahren angetreten bin, hat noch Bestand. Die Kommerzialisierung unseres Berufsstandes ist eine Schande. Ich sehe in einem Patienten, egal welchen Alters, keinen Wirtschaftsfaktor. Ich behandle Menschen, kein Alter, keine Hautfarbe, keine Nationalität, kein Portemonnaie. Dies wird belächelt, aber eine Medizin ohne Herz oder Empathie wird zur reinen technikverliebten Medizin herabgewürdigt. Meiner Zukunft als Patientin sehe ich mit großer Sorge entgegen.<br><br>GP-198: 14 - 14 (0)	Wenn jemand ein Leben lang für andere da gewesen ist, sollte man erwarten können, das diese im Alter ein wenig davon zurück bekommen....<br><br>D-415: 13 - 13 (0)<br><br>Aber ebenso wichtig ist mehr Empathie. Man muss seine Mitmenschen mögen, damit man sich für Sie einsetzt.ich findv<br><br>D-427: 12 - 12 (0)
**Organisationsstrukturen**			
Vergütung	adäquate Vergütung für den (erhöhten) Aufwand	keine Budgetierung von Physio und Ergotherapie	Förderung des mobilen Zahnmedizin, die in der aufsuchenden Betreung in privaten Haushalten sehr, sehr aufwendig, zeitintensiv und unterbezahlt ist

Specialists	General practitioners	Dentists
S-163: 12 - 12 (0)<br><br>mehr geld........<br><br>S-217: 8 - 8 (0)<br><br>mehr geld<br><br>S-217: 18 - 18 (0)<br><br>mehr zeit für die älteren Patienten nehmen und entsprechend in allen Fachrichtungen honoriert werden!!<br><br>S-222: 15 - 15 (0)<br><br>Gesellschaft und Krankenkassen machen sich zunehmend keine Gedanken über eine auskömmliche Finanzierung (z. B. Honorare) in der Versorgung.<br>Als Ärztin und Arzt wird man zunehmend nur ausgequetscht und ausgenutzt vom "Gesundheitswesen" (Politik, GKV, PKV).<br><br>S-223: 9 - 10 (0)<br><br>, auskömmliche und auch lukrative Honorierung.<br><br>S-223: 15 - 15 (0)<br><br>um alte Menschen, wenn wenig mobil und alleinstehend, länger zu Hause leben lassen zu können, sind ambulante Betreuung, pflegerisch wie ärztlich, so zu honorieren, dass es unternehemerisch machbar ist<br><br>S-226: 17 - 17 (0)<br><br>"sprechende Medizin" ist bei sehr alten Menschen auch im fachärztlichen Bereich wichtig und zu unterstützen/honorieren<br><br>S-226: 18 - 18 (0)	GP-192: 24 - 24 (0)<br><br>bessere Bezahlung von Pflegekräften<br><br>GP-192: 25 - 25 (0)<br><br>Bessere Honorierung der sprechenden Medizin<br><br>GP-202: 23 - 23 (0)<br><br>Verbesserung der Recurcen<br><br>GP-275: 15 - 15 (0)<br><br>mehr Geld<br><br>GP-310: 12 - 12 (0)<br><br>Billigmedizin tötet<br><br>GP-310: 13 - 13 (0)<br><br>mehr Zeit und Geld für HAusbesuche,<br><br>GP-506: 4 - 4 (0)<br><br>- mehr Berücksichtigung von Zeit z.B. in der Pflege/Medizin (Gespräche/Zeit mit den Menschen besser/wertender berücksichtigen)<br><br>GP-524: 23 - 23 (0)	D-371: 15 - 15 (0)<br><br>Liebevoller und zeitlich angepasster Umgang, ohne dass der Arzt einen wirtschaftlichen Verlust erleidet. Rückschluss: Honorar muss angepasst werden. Dies auch bei Patienten, die nicht in einer SGV XI Einrichtung leben, sondern in häuslicher Betreuung sind.<br><br>D-374: 4 - 4 (0)<br><br>Sonder Zulage bie demenziell veränderten Patienten. Abstufung der Pflegegrade sollte bei Abrechnung auch berücksichtigt werden.<br><br>D-374: 13 - 13 (0)<br><br>Mehr bezahlte Prophylaxe<br><br>D-376: 11 - 11 (0)<br><br>Honorieung für die Behandlung älterer Menschen anpassen.<br><br>D-382: 12 - 12 (0)<br><br>die Abrechnung der Leistungen bei diesen Patienten, vor allem der Pat. mit Pflegegrad, und bei Pat. in ambulanter Pflege muss gesondert betrachtet werden. Der Weg der Versorgung ist sehr viel aufwendiger als bei Pat. ohne Beieinträchtigungen<br><br>D-383: 14 - 14 (0)<br><br>Bessere Vergütung für zahnärztliche Leistungen! Es ist ein elementarer Unterschied ob ich einen 21-jährigen Sportstudenten operiere oder einen 91-jährigen Greis.<br><br>D-420: 27 - 27 (0)<br><br>Sprechende Zahnheilkunde muss endlich bezahlt werden !!!<br><br>D-420: 28 - 28 (0)<br><br>Die Einsicht,dass eine gute ambulante Betreuung mehr ausgebildetes Personal und Geld benötigt.<br><br>D-427: 12 - 12 (0)

Specialists	General practitioners	Dentists
bessere Vergütung für die Ärzte, da mehr Zeitaufwand<br><br>S-231: 10 - 10 (0)<br><br>Höhere und extrabudgetäre Vergütung<br><br>S-231: 12 - 12 (0)<br><br>Mehr Zeit für diese Patienten, respektive besseres Honorar, damit mehr Zeit genommen werden kann.<br><br>S-232: 12 - 12 (0)<br><br>Warum bekommen nur Hausärzte Zusatzpauschalen für Geriatische Patienten, für Polipharmazieberatung, für Demenzversorgung. Die tatsächliche Versorgung findet dann meist bei verschiedenen grundversorgenden Fachärzten statt, die den gleichen zeitlichen Aufwand haben wie der Hausarzt.<br><br>S-232: 13 - 13 (0)<br><br>Ausreichend Zeit und Vergütung<br><br>S-322: 12 - 12 (0)<br><br>Bessere Vergütung<br><br>S-469: 12 - 12 (0)<br><br>Es muss mehr Geld seitens der Krankenkassen für die Versorgung älterer Menschen bereitgestellt werden<br><br>S-469: 13 - 13 (0)<br><br>bessere Vegütung für alte/kranke Patienten,<br><br>S-470: 12 - 12 (0)		Viel mehr Zeit für die ambulante Pflege und bessere Bezahlung für diesen harten Job<br><br>D-433: 12 - 12 (0)<br><br>Eine Alterszahnmedizin benötigt deutlich mehr Einsatz und Zeitaufwand der sich in der Honorierung wiederspiegeln müsste<br><br>D-435: 12 - 12 (0)<br><br>Mehr Zeit und Arbeit und damit gerechte Entlohnung investieren,<br><br>D-436: 12 - 12 (0)<br><br>Bessere Vergütung für die Zeit, die man mehr aufwenden muss um Demenzkranke zu Behandeln.<br><br>D-439: 19 - 19 (0)<br><br>, eine bessere finanzielle Bewertung der Mundhygiene in der Pflege.<br><br>D-447: 17 - 17 (0)

	Specialists	General practitioners	Dentists
Mehr Zeit	meht zeit<br><br>S-217: 17 - 17 (0)<br><br>Mehr Zeit<br><br>S-221: 12 - 12 (0)<br><br>mehr zeit für die älteren Patienten nehmen und entsprechend in allen Fachrichtungen honoriert werden!!<br><br>S-222: 15 - 15 (0)<br><br>Mehr Zeit für diese Patienten, respektive besseres Honorar, damit mehr Zeit genommen werden kann.<br><br>S-232: 12 - 12 (0)<br><br>Ausreichend Zeit und Vergütung<br><br>S-322: 12 - 12 (0)<br><br>mehr Zeit<br><br>S-499: 12 - 12 (0)	Mehr Zeit pro Patient<br><br>GP-244: 27 - 27 (0)<br><br>Mehr Zeit / längere Sprechstunde<br><br>GP-253: 15 - 15 (0)<br><br>mehr Zeit und Geld für HAusbesuche,<br><br>GP-506: 4 - 4 (0)<br><br>- mehr Berücksichtigung von Zeit z.B. in der Pflege/Medizin (Gespräche/Zeit mit den Menschen besser/wertender berücksichtigen)<br><br>GP-524: 23 - 23 (0)	Förderung des mobilen Zahnmedizin, die in der aufsuchenden Betreung in privaten Haushalten sehr, sehr aufwendig, zeitintensiv und unterbezahlt ist<br><br>D-371: 15 - 15 (0)<br><br>Viel mehr Zeit für die ambulante Pflege und bessere Bezahlung für diesen harten Job<br><br>D-433: 12 - 12 (0)<br><br>Eine Alterszahnmedizin benötigt deutlich mehr Einsatz und Zeitaufwand der sich in der Honorierung wiederspiegeln müsste<br><br>D-435: 12 - 12 (0)<br><br>Mehr Zeit und Arbeit und damit gerechte Entlohnung investieren,<br><br>D-436: 12 - 12 (0)
Zugang	mehr Versorgung zuhause,<br><br>S-499: 12 - 12 (0)<br><br>schnellere Kontaktmöglichkeiten,<br><br>S-499: 12 - 12 (0)	leichterer Zugang zu ambulanten Hilfen<br><br>GP-192: 23 - 23 (0)<br><br>keine Budgetierung von Physio und Ergotherapie<br><br>GP-192: 24 - 24 (0)	Verbesserung der Versorgung von immobilen Patienten= aufsuchende Versorgung<br><br>D-399: 13 - 13 (0)<br><br>Festsetzung eines Zahnarztbesuches, wie z.B. bei den U-Untersuchungen der Kinder; Bezuschussung von Mini-Implantaten bei Zahnlosigkeit<br><br>D-416: 21 - 21 (0)<br><br>Barrierefreie Zugänglichkeit der entsprechenden Einrichtungen<br>D-449: 12 - 12 (0)

	Specialists	General practitioners	Dentists
			wieder mehr Hausbesuche<br>D-521: 24 - 24 (0)
Hilfs- und Angebots-strukturen	sowie Angebote zur Barrierefreiheit bei Ausklärungs- und Anamneseerfassung für die Patienten<br><br>S-169: 12 - 12 (0)<br><br>Ansprechpartner die das Nötige tatkräftig umsetzen<br><br>S-175: 13 - 13 (0)<br><br>wünscheswert wäre Unterstützung bei Mobiliät, um überhaupt Facharztbehandlugn möglich zu machen<br><br>S-196: 5 - 5 (0)<br><br>mehr ortsnahe Angebote und auch Angebote für Menschen, die nicht pflegebedürftig und dement sind<br><br>S-196: 6 - 6 (0)<br><br>niedrigschwellige Hilfsangebote<br><br>S-470: 12 - 12 (0)	leichterer Zugang zu ambulanten Hilfen<br><br>GP-192: 23 - 23 (0)<br><br>Ausbau ambulanter sozialer Hilfsdienste<br><br>GP-192: 26 - 26 (0)<br><br>Verbesserung der häuslichen Versorgung<br><br>GP-244: 28 - 28 (0)<br><br>Verbesserung der sozialen Anbindung<br><br>GP-244: 29 - 29 (0)<br><br>gutes soziales Umfeld des alternden Menschen über Familie, Freunde, Sport, Kunst/Kultur, Senioerntreffen, Ehrenamt, Aktivitäten<br><br>GP-245: 12 - 12 (0)<br><br>mehr Möglichkeiten der Tagespflege<br><br>GP-271: 12 - 12 (0)<br><br>Sozialstationsstrukturen wie zB Gemeindeschwester , Physio und Einkaufsdienste etc in Zusammenarbeit mit Arztpraxen siehe Skandinavien<br><br>GP-508: 14 - 15 (0)<br><br>Maßnahmen gegen Einsamkeit<br><br>GP-520: 25 - 25 (0)<br><br>zur Bewegung motivierende Maßnahmen<br><br>GP-520: 26 - 26 (0)<br><br>Patienten Lotsen die organisatorische Aufgaben übernehmen und dem älteren Patienten ein guter und informierter Ansprechpartner sind	Verbesserung der Versorgungsmöglichkeit für Patienten in häuslicher Pflege<br><br>D-362: 12 - 12 (0)<br><br>Mehr ProphlyaxeMaßnahmen für Senioren<br><br>D-367: 19 - 19 (0)<br><br>Pilotprojekte für Immobilie Patienten die zu Hause gefpelgt werden<br><br>D-367: 20 - 20 (0)<br><br>Förderung des mobilen Zahnmedizin, die in der aufsuchenden Betreung in privaten Haushalten sehr, sehr aufwendig, zeitintensiv und unterbezahlt ist<br><br>D-371: 15 - 15 (0)<br><br>Entbürokratisierung und Anpassung an häusliche Pflege zu Einrichtungen.<br><br>D-374: 13 - 13 (0)<br><br>Bessere Ausstattung in Pflege Heimen,<br><br>D-395: 14 - 14 (0)<br><br>Schnellere Hilfe Angebote bei der häuslichen Betreuung<br><br>D-395: 15 - 15 (0)<br><br>Möglichkeit der Begleitung der Patienten durch Pflegepersonal oder Angehörige zur Behandlung.<br><br>D-399: 13 - 13 (0)<br><br>z.B. ein Zahnarztzimmer und -Stuhl im Seniorenheim<br><br>D-422: 12 - 12 (0)<br><br>Bessere Betreuung in den Senioreneinrichtungen wie zahnärztliche Behandlungseinheiten und entsprechende Räumlichkeiten.<br><br>D-423: 12 - 12 (0)

	Specialists	General practitioners	Dentists
		GP-158: 16 - 16 (0)<br><br>mehr Zeit und Geld für HAusbesuche,<br><br>GP-506: 4 - 4 (0)<br><br>besser Vernetzung mit Sozialdienst der Stadt und kirlichen Diensten,die BEsuche übernehmen können, Kontaktadresse (Schüler, die zu hause Fenrseher einstellen können)<br><br>GP-506: 4 - 4 (0)<br><br>ich glaube das größte PRoblem sind die fehlenden und unübersichtlichen Hilfeleistungen und dass PAtienten damit so alleine gelassen werden, es gibt auch für mich keine Ansprechpartner, die ich informieren kann, dass ein einsamer alter MEnsch einen Besuch braucht, oder verzweifelt, weil er Fernsehprogramme nicht programmieren kann...mit Beantragung von Hilfsleitsungen sind alle überfordert.<br><br>GP-506: 14 - 14 (0)<br><br>stationäre Geriatrien, die auch mal auf Polymedikation, Bedürfnisse der Patienten achten, die mit den Möglichkeiten der ambulanten Medizin besser vertraut sind<br><br>GP-512: 19 - 19 (0)<br><br>das für die Menschen ohne häusliche Betreuung z.B. ein ambulanter Dienst einfach buchbar ist und über die Kasse problemlos abgerechnet werden darf.<br><br>GP-514: 12 - 12 (0)<br><br>- unbürokratischere Einleitung von Unterstützung<br><br>GP-524: 22 - 22 (0)	altersgerechte Wohnformen,<br><br>D-451: 24 - 24 (0)<br><br>Kommunikationsmöglichkeiten (z.B. WLAN im Altenheim)<br><br>D-451: 24 - 24 (0)<br><br>die zahnärztlicheVersorgung von Pflegeheimen ist in den letzten Jahren strukturell gesichert worden.<br>Die ambulante Pflege ist noch ein weites Feld .<br><br>D-451: 25 - 26 (0)<br><br>wieder mehr Hausbesuche<br><br>D-521: 24 - 24 (0)
Informations-austausch und interdisziplinäre Zusammen-arbeit	Bereitstellen einer Gesundheitsakte mit allen Informationen des Patienten wie Vorerkrankungen, Vor-OPs, Medikamente und Befunde von Untersuchungen<br><br>S-169: 4 - 4 (0)	Eine Verbesserung der kollegialen Zusammenarbeit wäre ebenfalls weiterführend, gerade in Bezug auf Krankenhausärzte. Der früher übliche kollegiale telefonische Austausch findet leider höchst selten statt.<br><br>GP-198: 15 - 15 (0)	weitere Vernetzung der Mediziner untereinander, erleichterte Kommuntikation, Transparenz bei der Beurteilung der Medzinischen Situation des Patienten,<br><br>D-371: 15 - 15 (0)

	Specialists	General practitioners	Dentists
	eine digitale Patientenakte, die bei Bedarf abrufbar ist antelle des müsamen Zusammentragens von Befunden aus unterschiedlichen Quellen<br><br>S-169: 12 - 12 (0)<br><br>Verzahnung der Behandelnden und Pflegenden<br><br>S-221: 12 - 12 (0)<br><br>Verbesserte Kommunikation aller Therapeuten<br><br>S-516: 12 - 12 (0)	stationäre Geriatrien, die auch mal auf Polymedikation, Bedürfnisse der Patienten achten, die mit den Möglichkeiten der ambulanten Medizin besser vertraut sind<br><br>GP-512: 19 - 19 (0)	bessere Kommunikation<br><br>D-395: 14 - 14 (0)<br><br>engere Zusammenarbeit HA /ZA<br><br>D-407: 12 - 12 (0)<br><br>Zusammenarbeit zwischen Hausärzten, mobilen Pflegediensten und Zahnärzten<br><br>D-454: 14 - 14 (0)
Entbüro-kratisierung, Vereinfachung			kürzere bürokratische Wege<br><br>D-383: 13 - 13 (0)<br><br>Abbau der Bürokratie<br><br>D-434: 12 - 12 (0)
Aus-, Fort- und Weiterbildung	Fortbildungen Geroatrie<br><br>S-163: 13 - 13 (0)<br><br>Wertschätzung und Motivation für den Pflegeberuf, Wertschätzung und Motivation für den Arztberuf,<br><br>S-196: 17 - 17 (0)	Weitere Forschung an der KI Und sinnvoller Einsatz auch in der Pflege<br><br>GP-219: 12 - 12 (0)	Neues Pflegegesetzt. Neue Verpflichtung der Einrichtung und der Betreuer !!!!!, sowie der Angehörigen.<br><br>D-374: 13 - 13 (0)<br><br>die Personalsituation und die Ausbildung der Pflegekräfte in der Pflegeheimen bezüglich der zahnmedizinischen Versorgung muss sich bessern!<br><br>D-384: 12 - 12 (0)

	Specialists	General practitioners	Dentists
	bitte nicht noch mehr Pseudoqualifikationen wie Geriatrische Grundversorgung oder Palliativmedizin<br>S-196: 18 - 18 (0)		Wertschätzung für die Arbeit, die unter schweren Bedingungen von Medizinern und Zahnmedizinern in Pflegeinreichtungen getätigt wird.<br><br>D-398: 12 - 12 (0)<br><br>Aufklärung der Pflegenden über Bedeutung der Mundhygiene.<br><br>D-399: 13 - 13 (0)<br><br>Rechtzeitige Aufnahme in die universitären Ausbildungen. Die Erkenntnis zu stärken ,wie schnell wir selbst dieser Altersgruppe angehören<br><br>D-427: 12 - 12 (0)<br><br>Die Einsicht,dass eine gute ambulante Betreuung mehr ausgebildetes Personal und Geld benötigt.<br><br>D-427: 12 - 12 (0)<br><br>Besser Ausbildung bei den Pflegekräften<br><br>D-439: 18 - 18 (0)<br><br>spezialisierte Ausbildung<br><br>D-521: 22 - 22 (0)
Mehr (qualifiziertes) Personal	Bessere Pflege<br><br>S-216: 16 - 16 (0)<br><br>Der sich abzeichnende Ärztemangel<br><br>S-216: 17 - 17 (0)<br><br>Mehr medizinische Personal in allen Berufsgruppen,<br><br>S-223: 15 - 15 (0)<br><br>mehr qualifiziertes Personal	Mehr medizinisches /Pflegepersonal.<br><br>GP-244: 26 - 26 (0)<br><br>Ich hätte gerne eine angestellte Ärztin, die mir die Praxisarbeit in Anteilen abnimmt, damit ich mehr Heimmedizin machen kann. Ich finde Geriatrie KLASSE!<br><br>GP-525: 15 - 15 (0)	die Personalsituation und die Ausbildung der Pflegekräfte in der Pflegeheimen bezüglich der zahnmedizinischen Versorgung muss sich bessern!<br><br>D-384: 12 - 12 (0)<br><br>personelle Aufrüstung<br><br>D-395: 14 - 14 (0)<br><br>mehr Verantwortung für med. zahnmed. Personal<br><br>D-521: 23 - 23 (0)

	Specialists	General practitioners	Dentists
	S-372: 12 - 12 (0)		
**Weiteres**			
Berücksichtigung der nicht-med. Dimensionen		Ausbau ambulanter sozialer Hilfsdienste<br><br>GP-192: 26 - 26 (0)<br><br>Hilfreich für meine tägliche Arbeit wäre, die sprechende Medizin aufzuwerten.<br><br>GP-198: 15 - 15 (0)<br><br>Auch und gerade bei den über 80-jährigen ist oft nicht so sehr die medizinische sondern die ärztliche Kompetenz gefordert.<br><br>GP-202: 24 - 24 (0)<br><br>Verbesserung der sozialen Anbindung<br><br>GP-244: 29 - 29 (0)<br><br>gutes soziales Umfeld des alternden Menschen über Familie, Freunde, Sport, Kunst/Kultur, Senioerntreffen, Ehrenamt, Aktivitäten<br><br>GP-245: 12 - 12 (0)<br><br>Sozialstationsstrukturen wie zB Gemeindeschwester , Physio und Einkaufsdienste etc in Zusammenarbeit mit Arztpraxen siehe Skandinavien<br><br>GP-508: 14 - 15 (0)<br><br>Maßnahmen gegen Einsamkeit<br><br>GP-520: 25 - 25 (0)<br><br>ich glaube das größte PRoblem sind die fehlenden und unübersichtlichen Hilfeleistungen und dass PAtienten damit so alleine gelassen werden, es gibt auch für mich keine Ansprechpartner, die ich informieren kann, dass ein einsamer alter MEnsch einen Besuch braucht, oder verzweifelt, weil er Fernsehprogramme nicht	Mehr Einsatz der Angehörigen<br><br>D-415: 12 - 12 (0)

	Specialists	General practitioners	Dentists
		programmieren kann...mit Beantragung von Hilfsleitsungen sind alle überfordert.<br><br>GP-506: 14 - 14 (0)	
Relevanz der Mund-gesundheit stärken			-Seperate Behandlungsräume in Wohnheimen mit hygienischen Konzepten, fließendem Wasser und einer einstellbaren Liegemöglichkeit für die Behandlung.<br><br>D-189: 29 - 29 (0)<br><br>Ein Raum in dem man sowohl Zähne behandeln als auch Zehennägel als auch Haare schneiden kann und der einfach zu reinigen ist!<br><br>D-189: 30 - 30 (0)<br><br>jede Einrichtung mit Hochbetagten braucht ein BEhandlungszimmer für die kooperierenden Zahnärzte und Ärzte<br><br>D-234: 13 - 13 (0)<br><br>Mehr ProphlyaxeMaßnahmen für Senioren<br><br>D-367: 19 - 19 (0)<br><br>Pilotprojekte für Immobilie Patienten die zu Hause gefpelgt werden<br><br>D-367: 20 - 20 (0)<br><br>Förderung des mobilen Zahnmedizin, die in der aufsuchenden Betreung in privaten Haushalten sehr, sehr aufwendig, zeitintensiv und unterbezahlt ist<br><br>D-371: 15 - 15 (0)<br><br>Aufklärung des Personals und der Angehörigen und Aufnahme der Mundhyigene in den Pflegestandard.<br><br>D-374: 4 - 4 (0)<br><br>Entbürokratisierung und Anpassung an häusliche Pflege zu Einrichtungen.<br><br>D-374: 13 - 13 (0)<br><br>Bessere Ausstattung in Pflege Heimen,<br><br>D-395: 14 - 14 (0)

	Specialists	General practitioners	Dentists
			Aufklärung der Pflegenden über Bedeutung der Mundhygiene.<br><br>D-399: 13 - 13 (0)<br><br>Umschwung tut Not. Es ist höchste Zeit für Veränderungen.<br>Die Zahnstaten und die Mundgesundheit in den Pflegeheimen die ich kenne lassen sehr (!) zu wünschen übrig.<br>Ebenso die Erfahrungen aus meiner 14- jährigen Konsilzahnarzt-Tätigkeit für ein 600 Betten Krankenhaus sprechen Bände.<br>Wir leben im reichsten Land Europas und sollten uns schämen so wenig ZahnMedizin für "die Alten" übrig zu haben.<br><br>D-420: 29 - 32 (0)<br><br>z.B. ein Zahnarztzimmer und -Stuhl im Seniorenheim<br><br>D-422: 12 - 12 (0)<br><br>Bessere Betreuung in den Senioreneinrichtungen wie zahnärztliche Behandlungseinheiten und entsprechende Räumlichkeiten.<br><br>D-423: 12 - 12 (0)<br><br>Die Anfänge sind in der Zahnmed.erfolgreich eingeleitet<br><br>D-436: 13 - 13 (0)<br><br>Besser Ausbildung bei den Pflegekräften<br><br>D-439: 18 - 18 (0)<br><br>, eine bessere finanzielle Bewertung der Mundhygiene in der Pflege.<br><br>D-447: 17 - 17 (0)<br><br>die zahnärztlicheVersorgung von Pflegeheimen ist in den letzten Jahren strukturell gesichert worden.<br>Die ambulante Pflege ist noch ein weites Feld .<br><br>D-451: 25 - 26 (0)
Anstehende Entwicklungen und Heraus-forderungen	Der sich abzeichnende Ärztemangel<br><br>S-216: 17 - 17 (0)	In meiner Rolle als Ärztin fühle ich mich als Fossil. Nichts von dem, weswegen ich vor 33 Jahren angetreten bin, hat noch Bestand. Die Kommerzialisierung unseres Berufsstandes ist eine Schande. Ich sehe in einem Patienten,	Pflege hat defintiv zu wenig Zeit für Mundhygiene. Es gibt keine vorsorgliche Anpassung der demografischen Veränderung und dem damit verbundenen Mehraufwand für die Ärzte. Equipment ist sehr kostenintensiv. Dies sollte gedeckelt werden!

	Specialists	General practitioners	Dentists
	es werden sich noch alle ganz furchtbar wundern, wenn die babyboomer in die jahre kommen<br>aber<br>das war ja nicht vorauszusehen :-)<br>politisches versagen auf der ganzen linie.....parteien aller coleur !<br><br>S-217: 20 - 23 (0)<br><br>Es muss mehr Geld seitens der Krankenkassen für die Versorgung älterer Menschen bereitgestellt werden<br><br>S-469: 13 - 13 (0)	egal welchen Alters, keinen Wirtschaftsfaktor. Ich behandle Menschen, kein Alter, keine Hautfarbe, keine Nationalität, kein Portemonnaie. Dies wird belächelt, aber eine Medizin ohne Herz oder Empathie wird zur reinen technikverliebten Medizin herabgewürdigt. Meiner Zukunft als Patientin sehe ich mit großer Sorge entgegen.<br><br>GP-198: 14 - 14 (0)<br><br>habe keine Hoffnung auf Verbesserung<br>Zu viele Hochbetagte treffen auf zu wenig Kümmerer und die Ansprüche Einzener demotivieren<br><br>GP-508: 16 - 17 (0)	D-374: 3 - 3 (0)<br><br>Nicht nur die Medizin, sondern die gesamte Gesellschaft muss sich auf immer älter werdende Menschen einstellen<br>und nicht aufs Abstellgleis schicken. Auch alte Menschen haben ihre Bedürfnisse. Sie sollten respektvoll behandelt<br>werden.<br><br>D-382: 13 - 15 (0)<br><br>Wichtiges Thema!<br><br>D-384: 13 - 13 (0)<br><br>Ich finde es gut,sich mit der Versorgung unserer steht's älter werdenden Mitmenschen zu befassen. Nur dann gibt es ein würdevolles und möglichst selbstbestimmtes Altern. Danke für Ihre Arbeit, dieses Thema über alle Fachrichtungen der ärztlichen und zahnärztlichen Tätigkeiten zu untersuchen um gute Behandlungsstrategien zu entwickeln und die kollegiale Zusammenarbeit zu stärken. Viel Erfolg<br><br>D-427: 13 - 13 (0)<br><br>Wir können froh sein in der richtigen Zeit in der richtigen Ecke auf der Welt leben zu dürfen.<br><br>D-451: 27 - 27 (0)
Unverständliche Fragen		die letzten drei Fragen habe ich nicht verstanden,<br><br>GP-506: 13 - 13 (0)	Haben Sie einen zahnmedizinischen Tätigkeitsschwerpunkt oder arbeiten Sie in einer Zahnmedizinischen Facharztpraxis?<br><br>Diese Frage hatte ja/nein als Auswahl<br><br>D-189: 31 - 32 (0)<br><br>Frage verstehe ich nicht!<br><br>D-432: 11 - 11 (0)
